# Supplementary figures and images for: α-/γ-Taxilin are required for centriolar subdistal appendage assembly and microtubule organization
Source: eLife. 2022 Feb 4;11:e73252. doi: 10.7554/eLife.73252 (PMC8816381; doi:10.7554/eLife.73252)

**Figure 1-figure supplement 1C**

Biotin

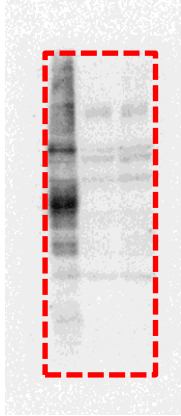

**Figure 1-figure supplement 1D**

Biotin

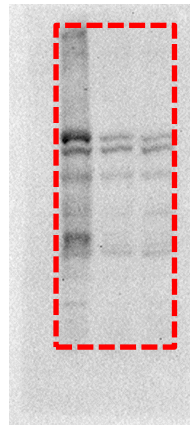

Supplement: Figure 1—figure supplement 1—source data 1. [file elife-73252-fig1-figsupp1-data1.zip › Figure 1-figure supplement 1-source data 1/labeled immuoblots for Figure 1-figure supplement 1.pdf]

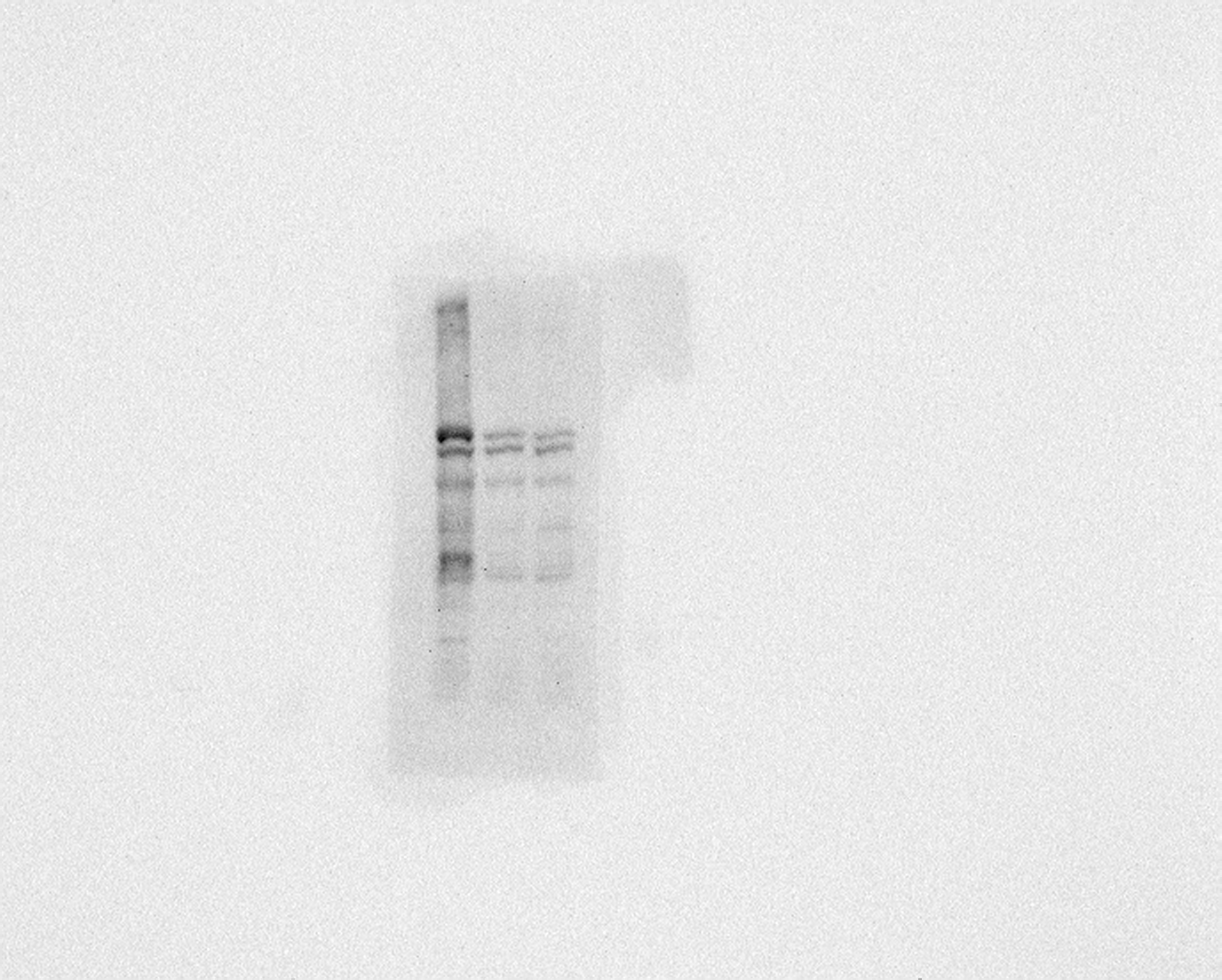

Supplement: Figure 1—figure supplement 1—source data 1. [file elife-73252-fig1-figsupp1-data1.zip › Figure 1-figure supplement 1-source data 1/Unlabeled immunoblots/ccdc120-v5-biotin-10 min-Figure 1-figure supplement 1D.Tif]

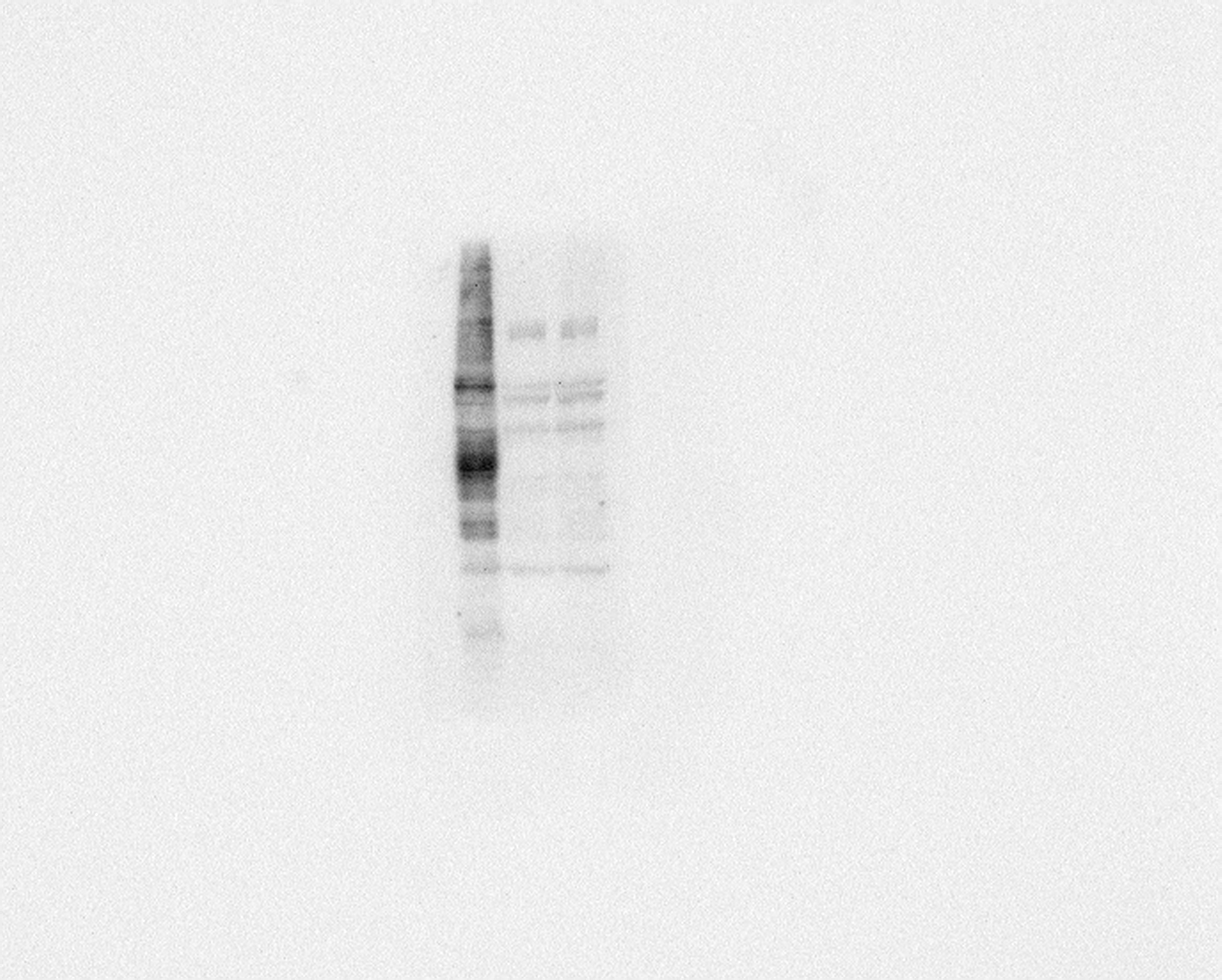

Supplement: Figure 1—figure supplement 1—source data 1. [file elife-73252-fig1-figsupp1-data1.zip › Figure 1-figure supplement 1-source data 1/Unlabeled immunoblots/CCDC68-5 ul-biotin-Figure 1-figure supplement 1C.Tif]

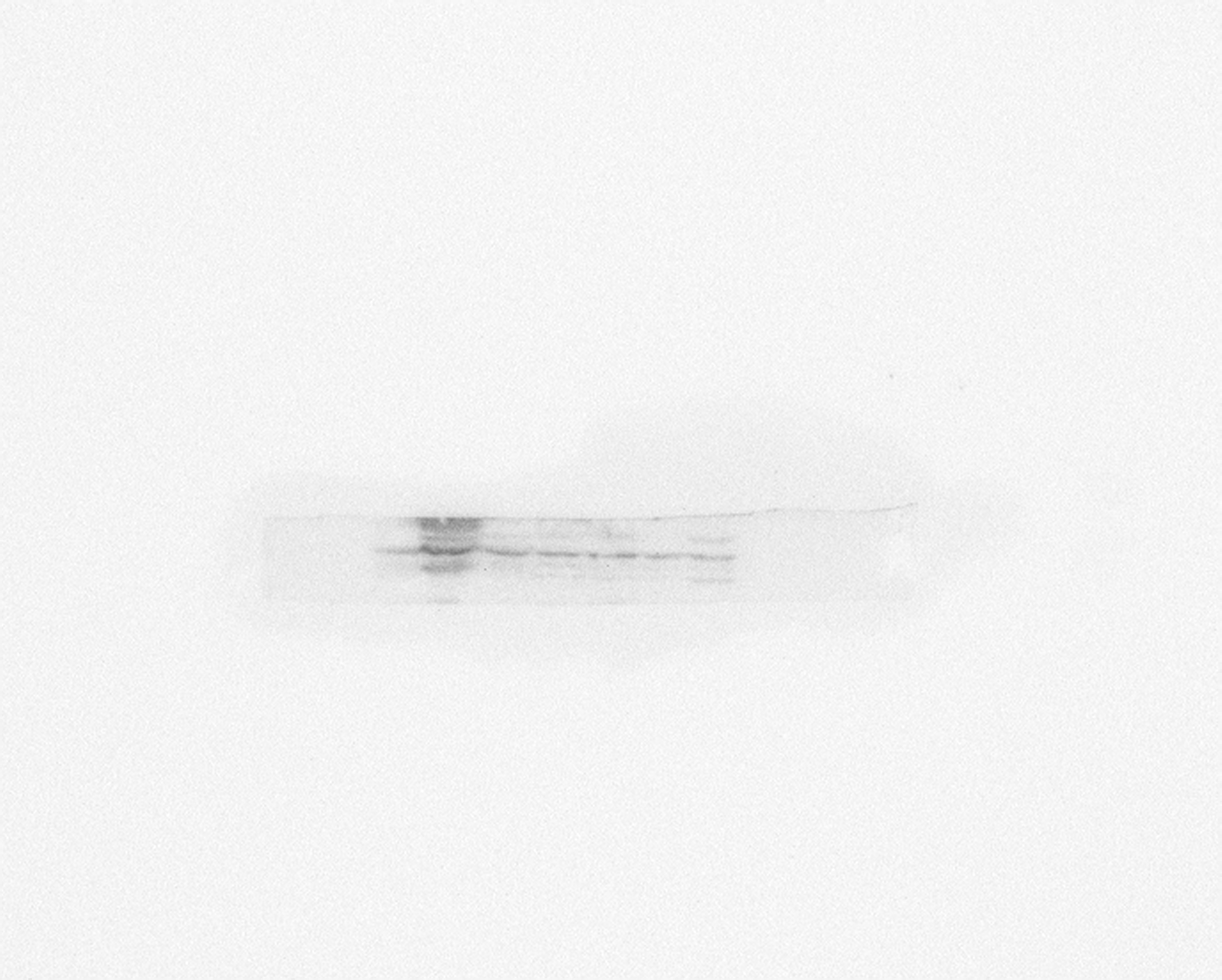

Supplement: Figure 1—figure supplement 2—source data 1. [file elife-73252-fig1-figsupp2-data1.zip › Figure 1-figure supplement 2-source data 1/Unlabeled immunoblots/GAPDH for TA2-Figure 1-figure suppment 2E.Tif]

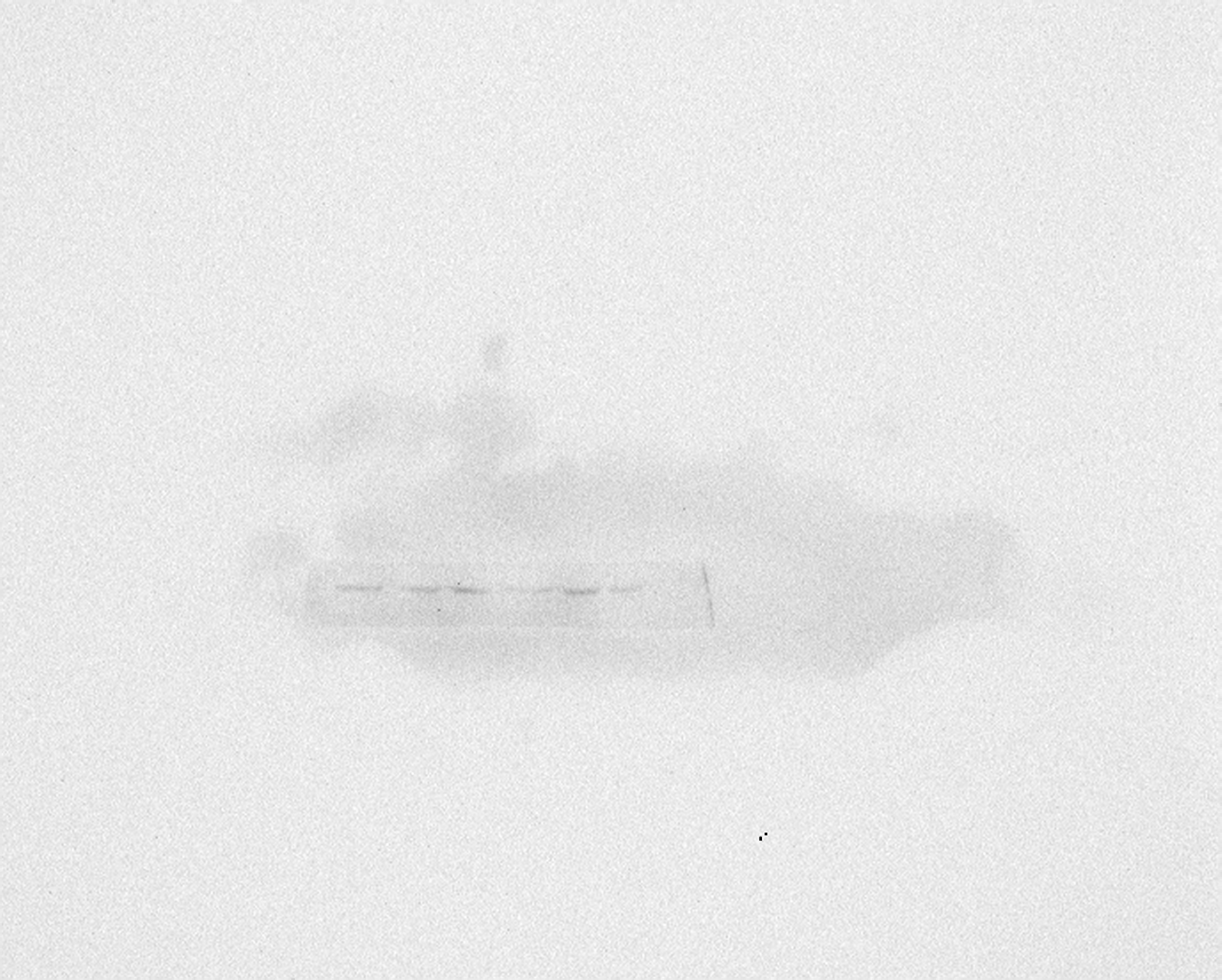

Supplement: Figure 1—figure supplement 2—source data 1. [file elife-73252-fig1-figsupp2-data1.zip › Figure 1-figure supplement 2-source data 1/Unlabeled immunoblots/GAPDH for TG-GFP-10 min-Figure 1-figure supplement 2F.Tif]

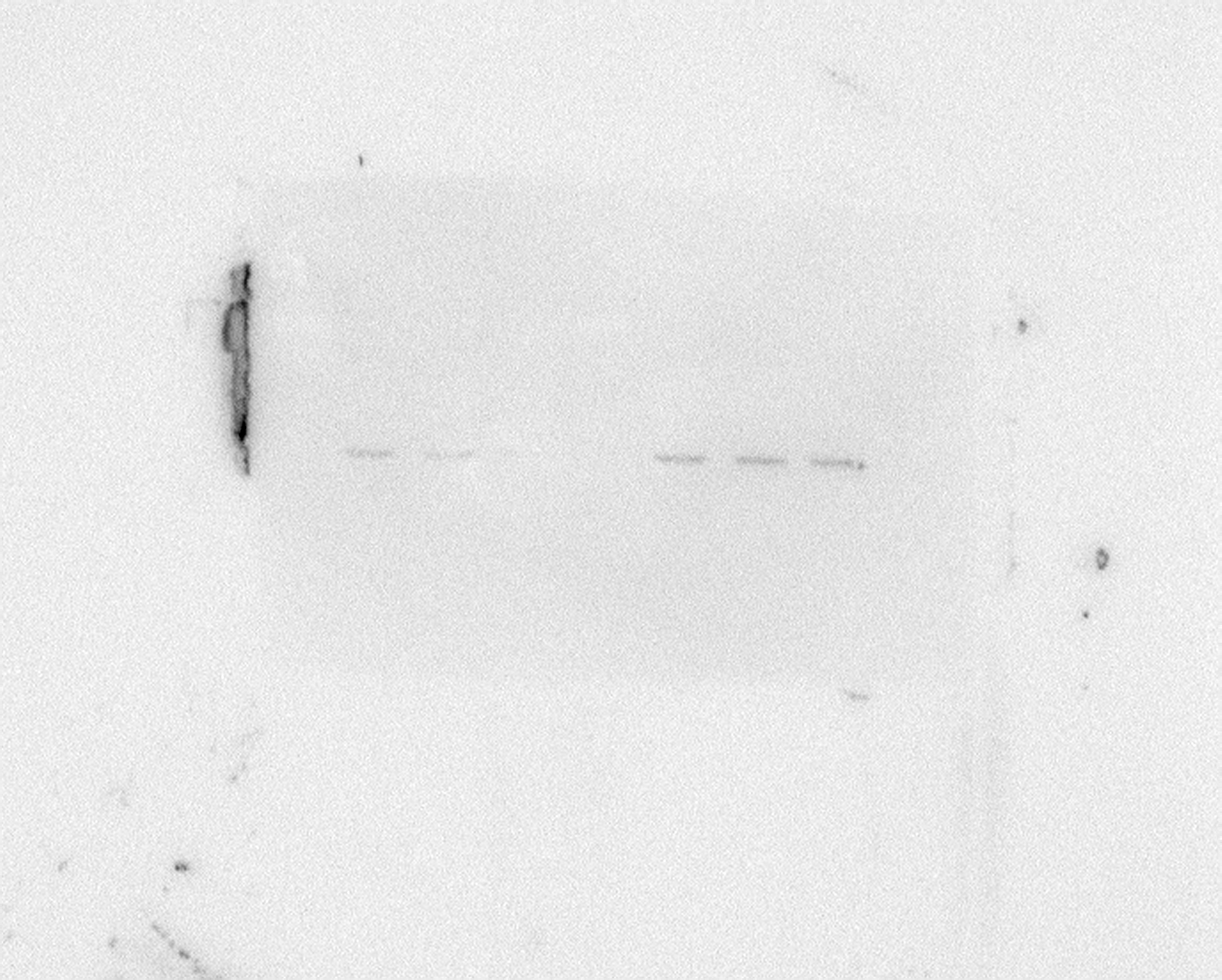

Supplement: Figure 1—figure supplement 2—source data 1. [file elife-73252-fig1-figsupp2-data1.zip › Figure 1-figure supplement 2-source data 1/Unlabeled immunoblots/GAPDH-SiRNA-HeLa-RPE1-Figure 1-figure supplement 2C.Tif]

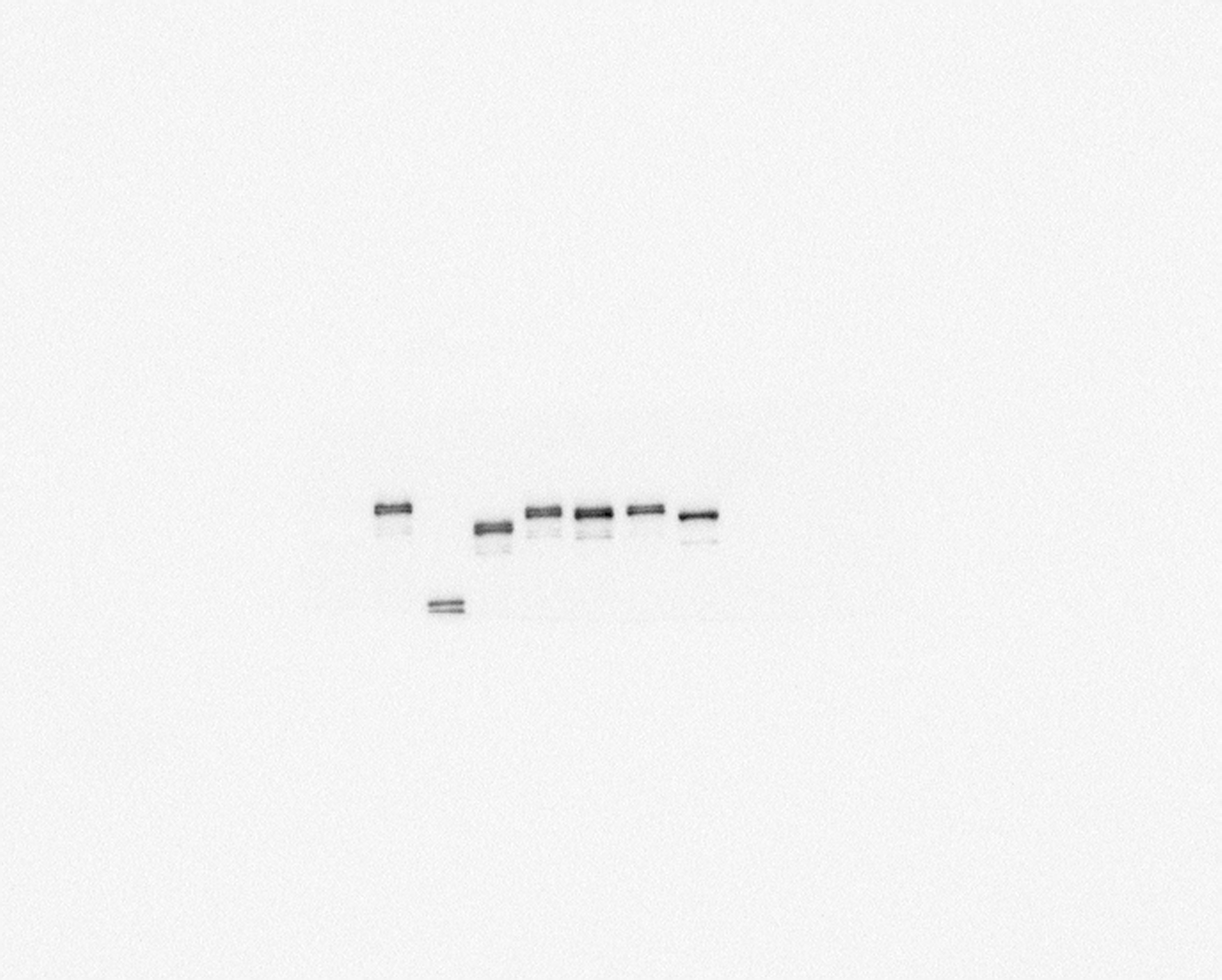

Supplement: Figure 1—figure supplement 2—source data 1. [file elife-73252-fig1-figsupp2-data1.zip › Figure 1-figure supplement 2-source data 1/Unlabeled immunoblots/TA-HA-90s-Figure 1-figure supplement 2E.Tif]

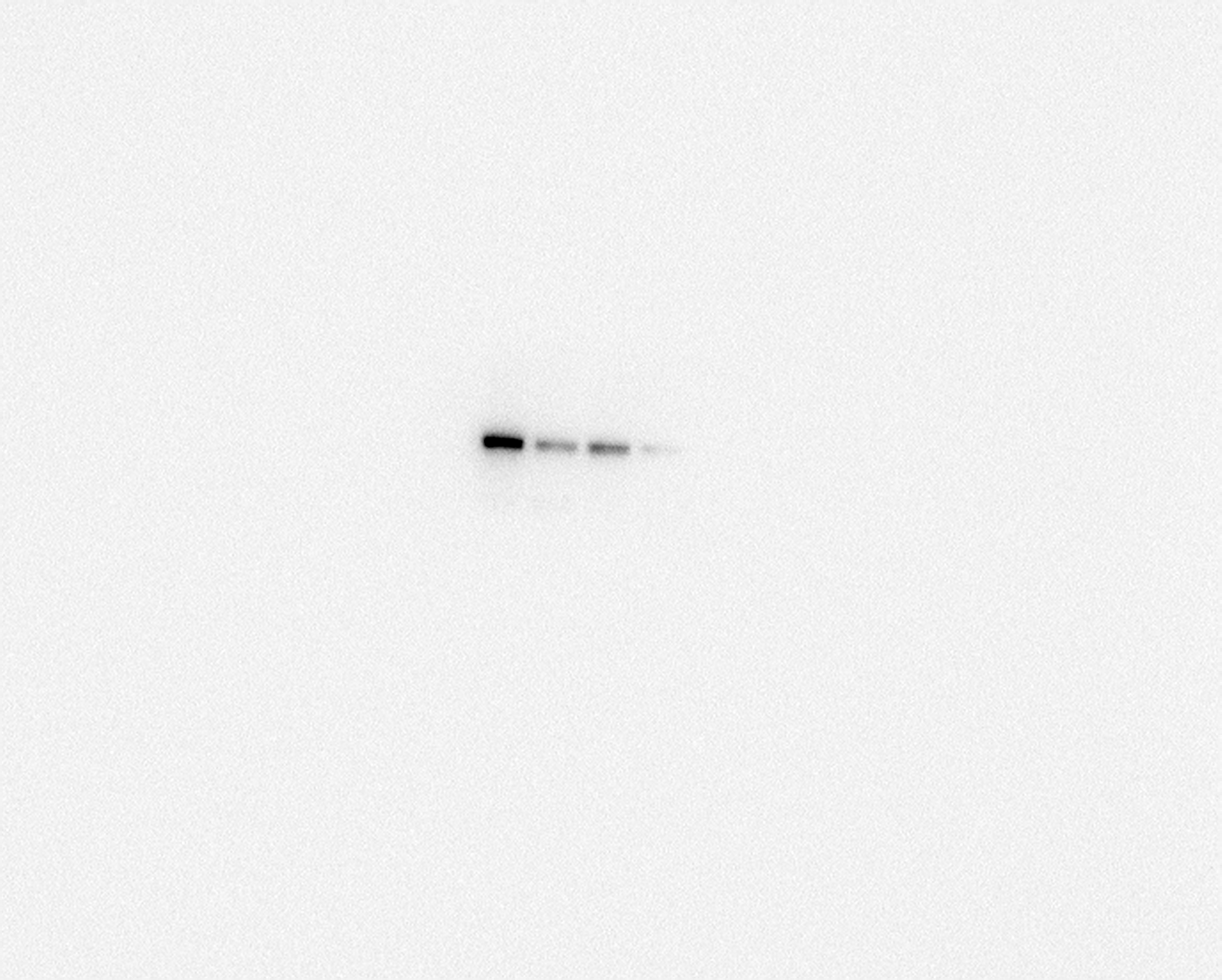

Supplement: Figure 1—figure supplement 2—source data 1. [file elife-73252-fig1-figsupp2-data1.zip › Figure 1-figure supplement 2-source data 1/Unlabeled immunoblots/TA-KD-TA-Figure 1-figure supplement 2B.Tif]

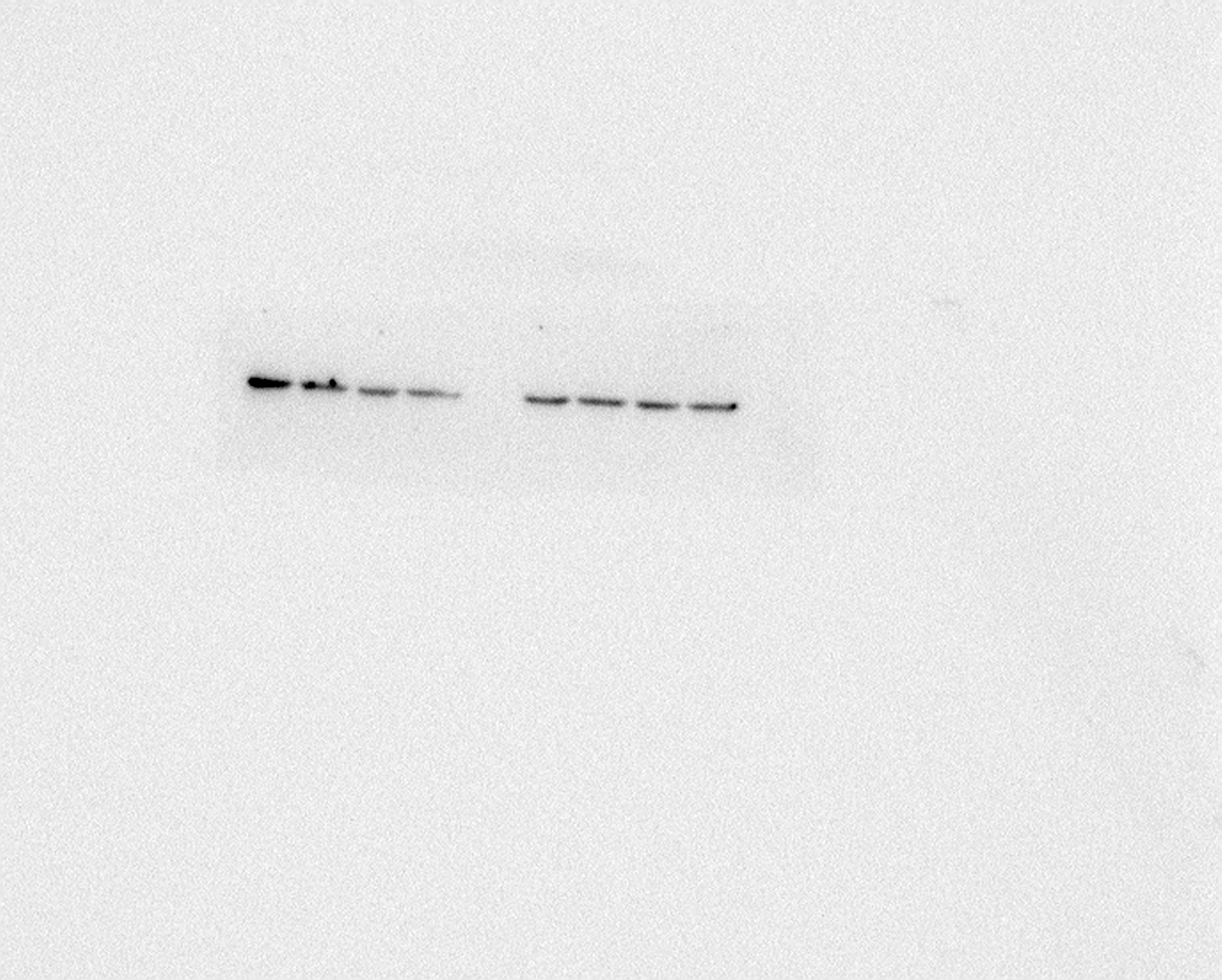

Supplement: Figure 1—figure supplement 2—source data 1. [file elife-73252-fig1-figsupp2-data1.zip › Figure 1-figure supplement 2-source data 1/Unlabeled immunoblots/TA-KD-TG3-Figure 1-figure supplement 2B.Tif]

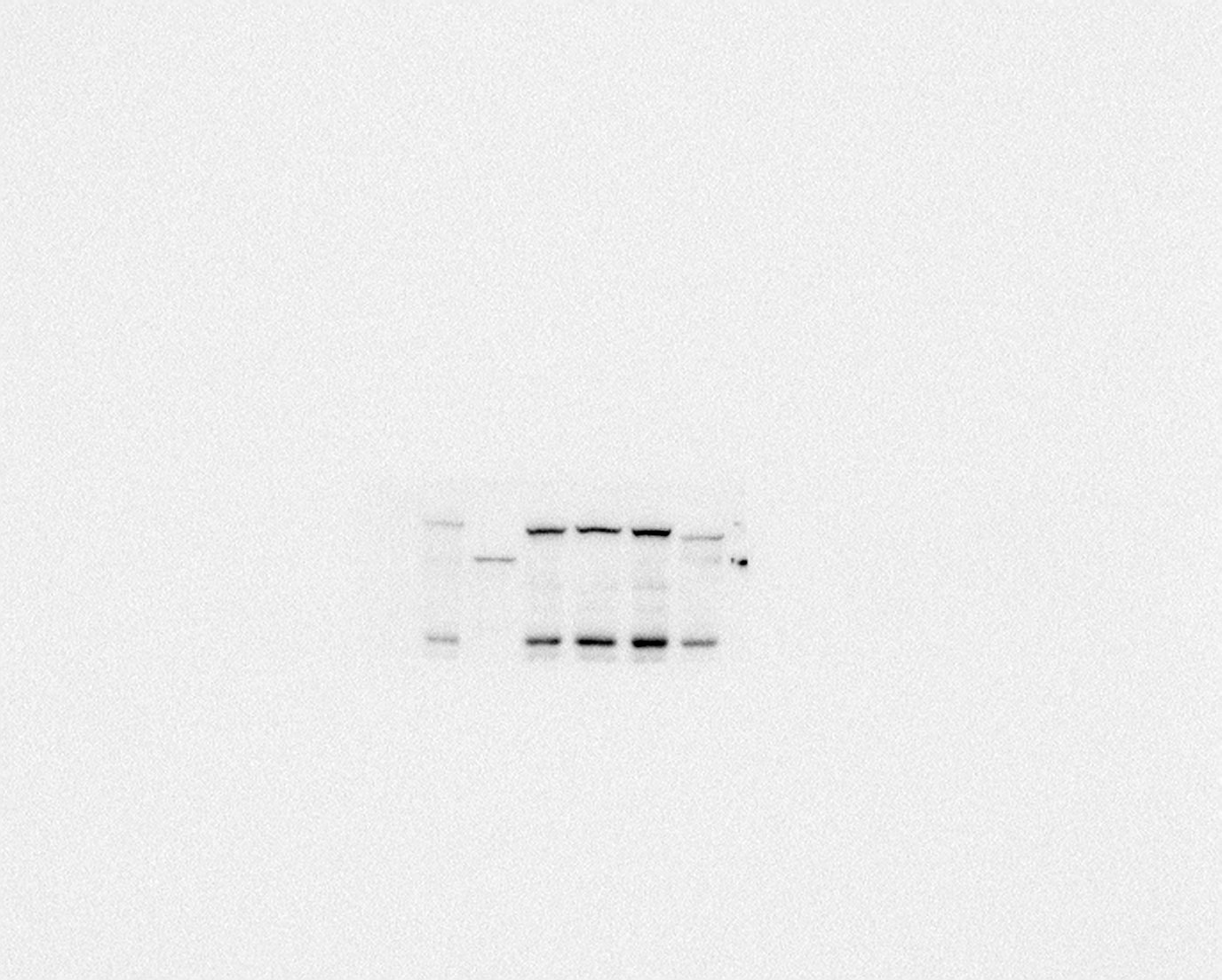

Supplement: Figure 1—figure supplement 2—source data 1. [file elife-73252-fig1-figsupp2-data1.zip › Figure 1-figure supplement 2-source data 1/Unlabeled immunoblots/TG-GFP-30s-Figure 1-figure supplement 2F.Tif]

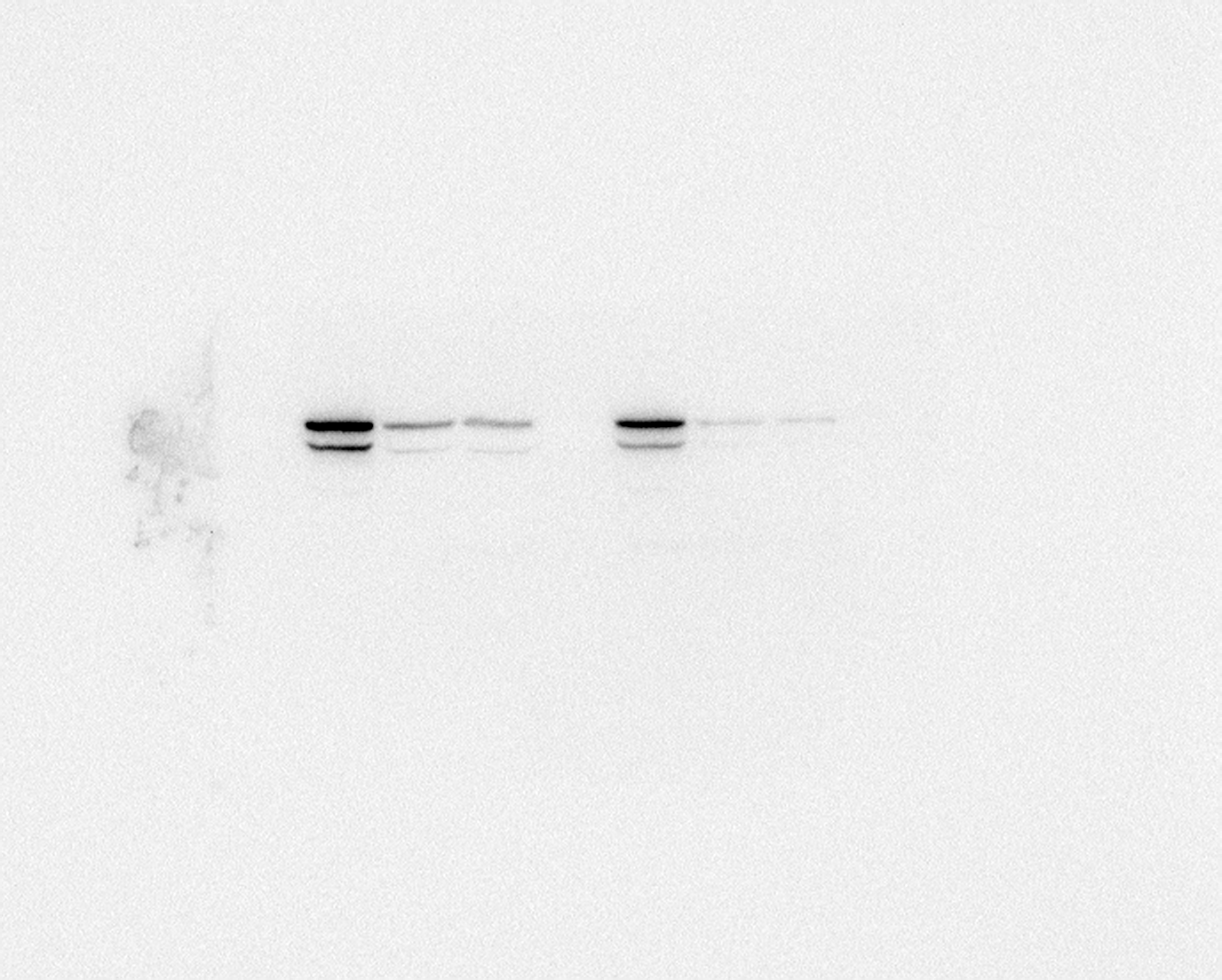

Supplement: Figure 1—figure supplement 2—source data 1. [file elife-73252-fig1-figsupp2-data1.zip › Figure 1-figure supplement 2-source data 1/Unlabeled immunoblots/TXLNG-SiRNA-HeLa-RPE1-Figure 1-figure supplement 2C.Tif]

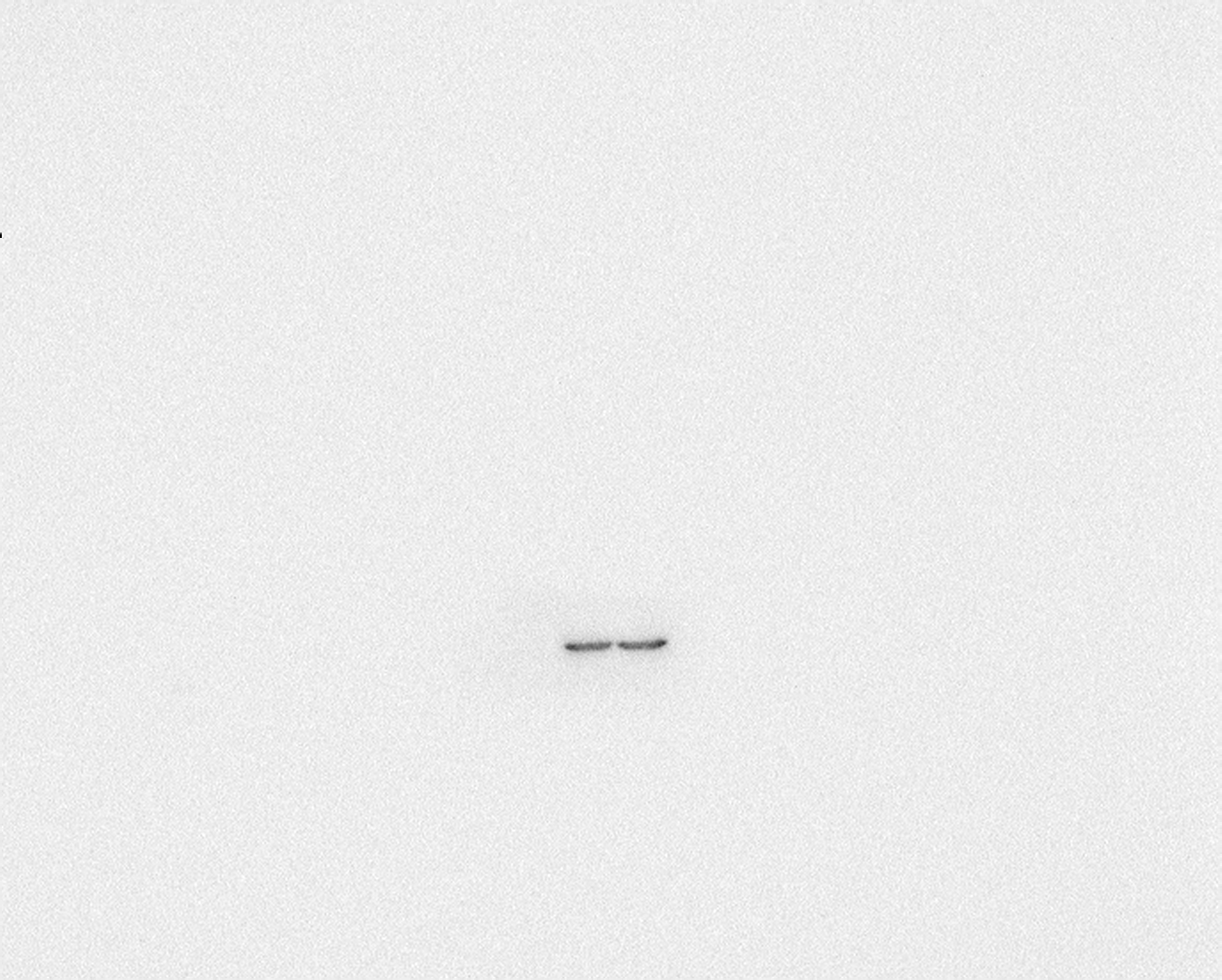

Supplement: Figure 3—source data 3. [file elife-73252-fig3-data3.zip › Figure 3-source data 3/Unlabeled immunoblots/GAPDH-10u-60s-Figure 3E.Tif]

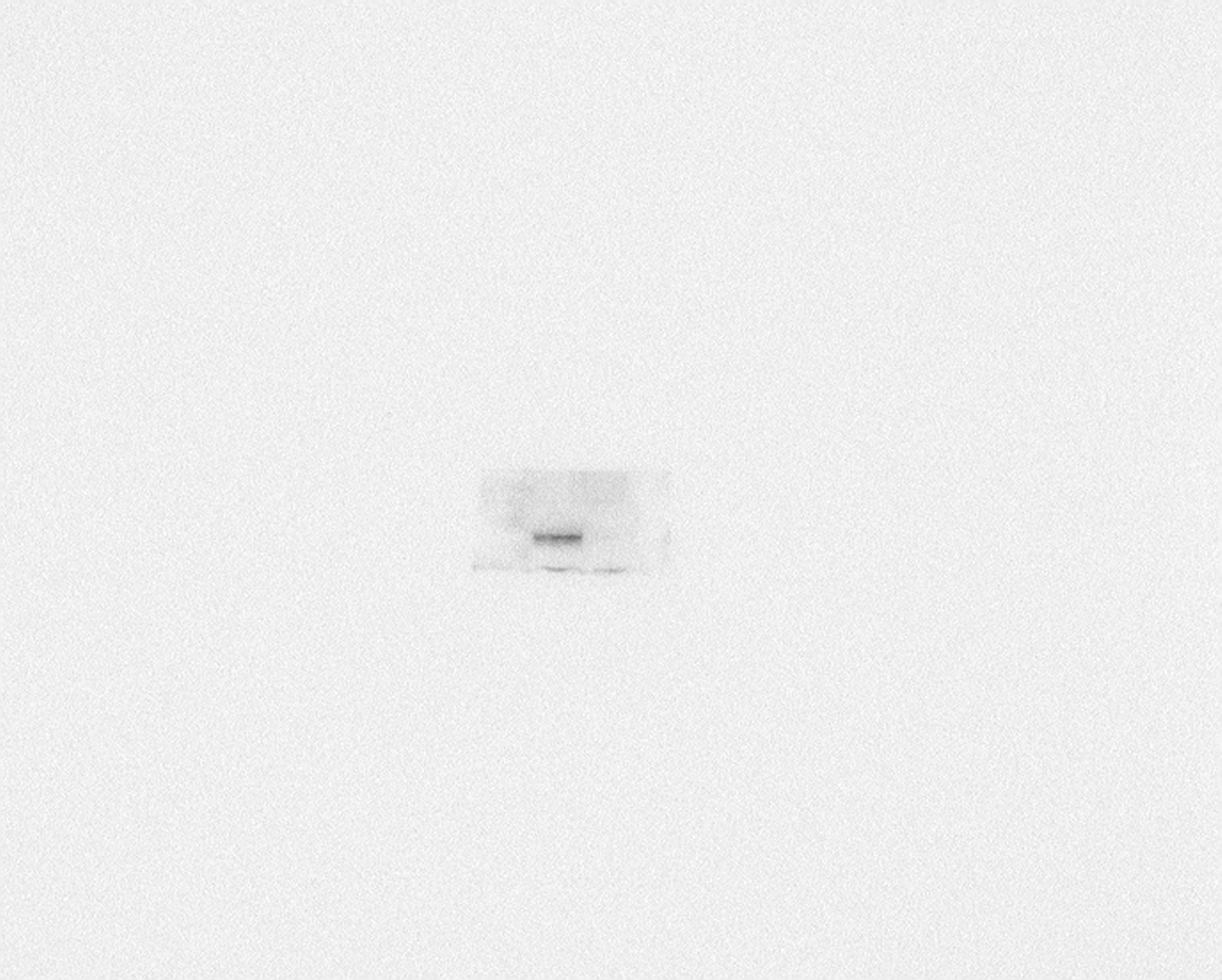

Supplement: Figure 3—source data 3. [file elife-73252-fig3-data3.zip › Figure 3-source data 3/Unlabeled immunoblots/ODF2-10u-30s-Figure 3E.Tif]

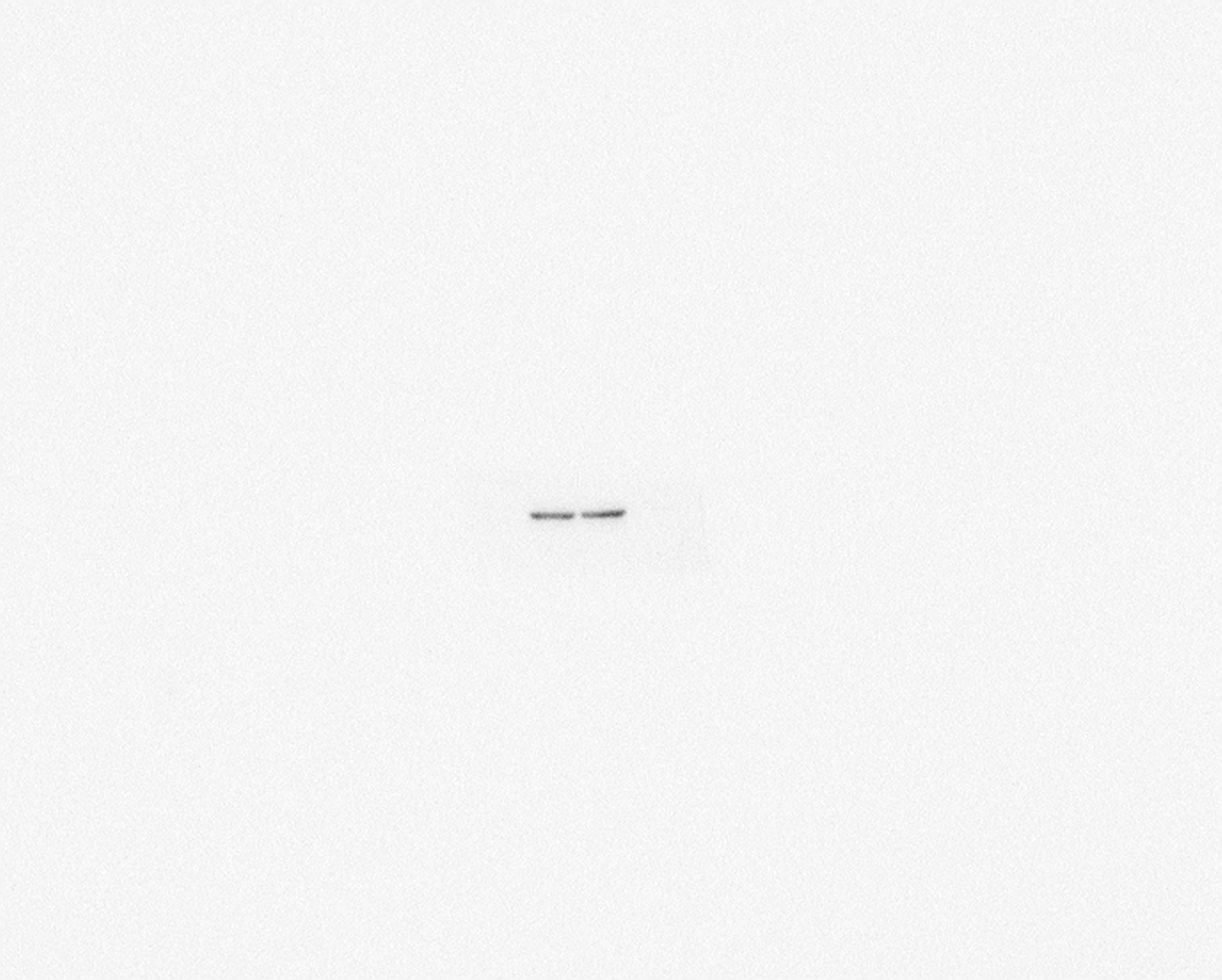

Supplement: Figure 3—source data 3. [file elife-73252-fig3-data3.zip › Figure 3-source data 3/Unlabeled immunoblots/TA-100s-Figure 3E.Tif]

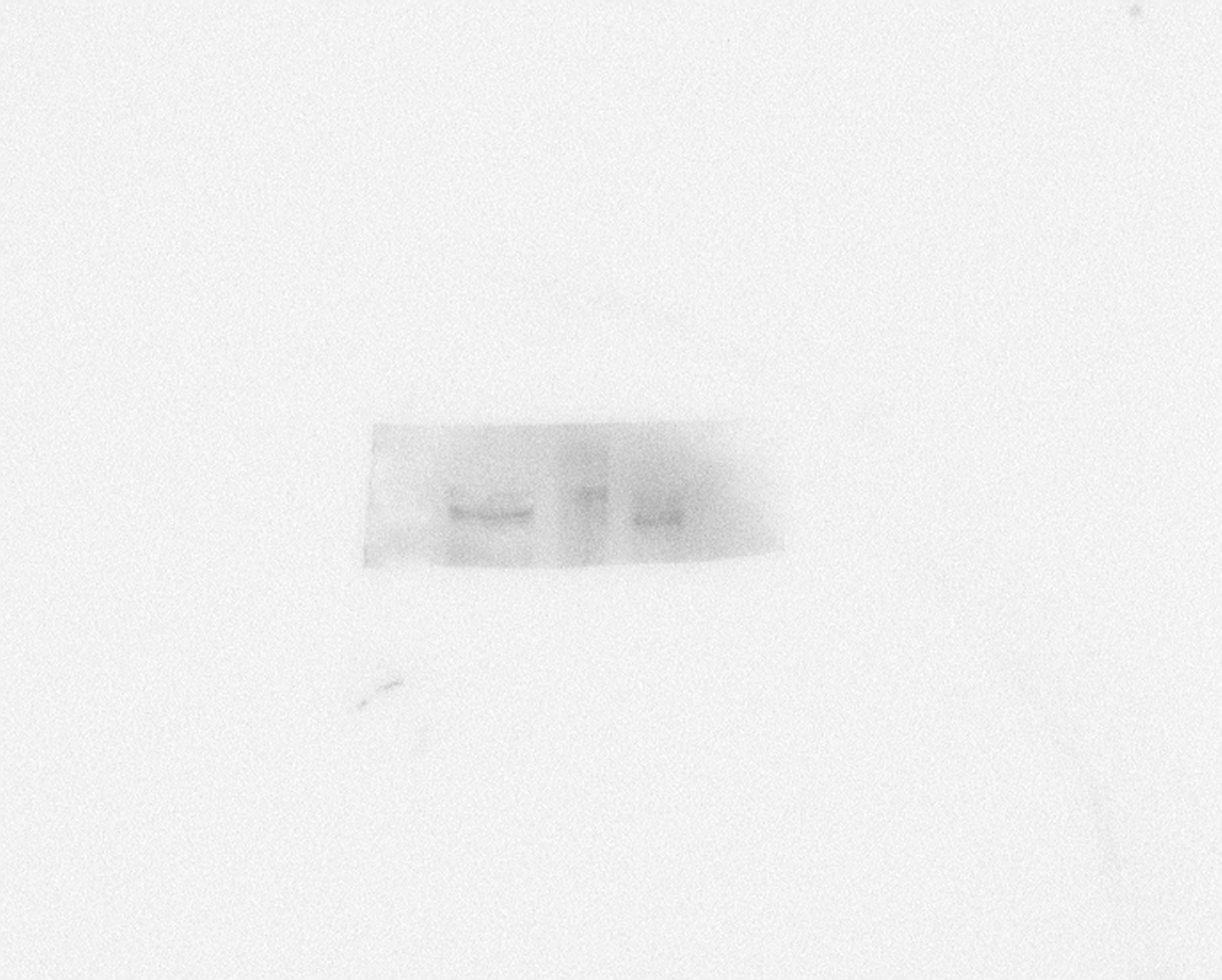

Supplement: Figure 3—source data 3. [file elife-73252-fig3-data3.zip › Figure 3-source data 3/Unlabeled immunoblots/TA-IP-odf2-RPE1-6-Figure 3A.Tif]

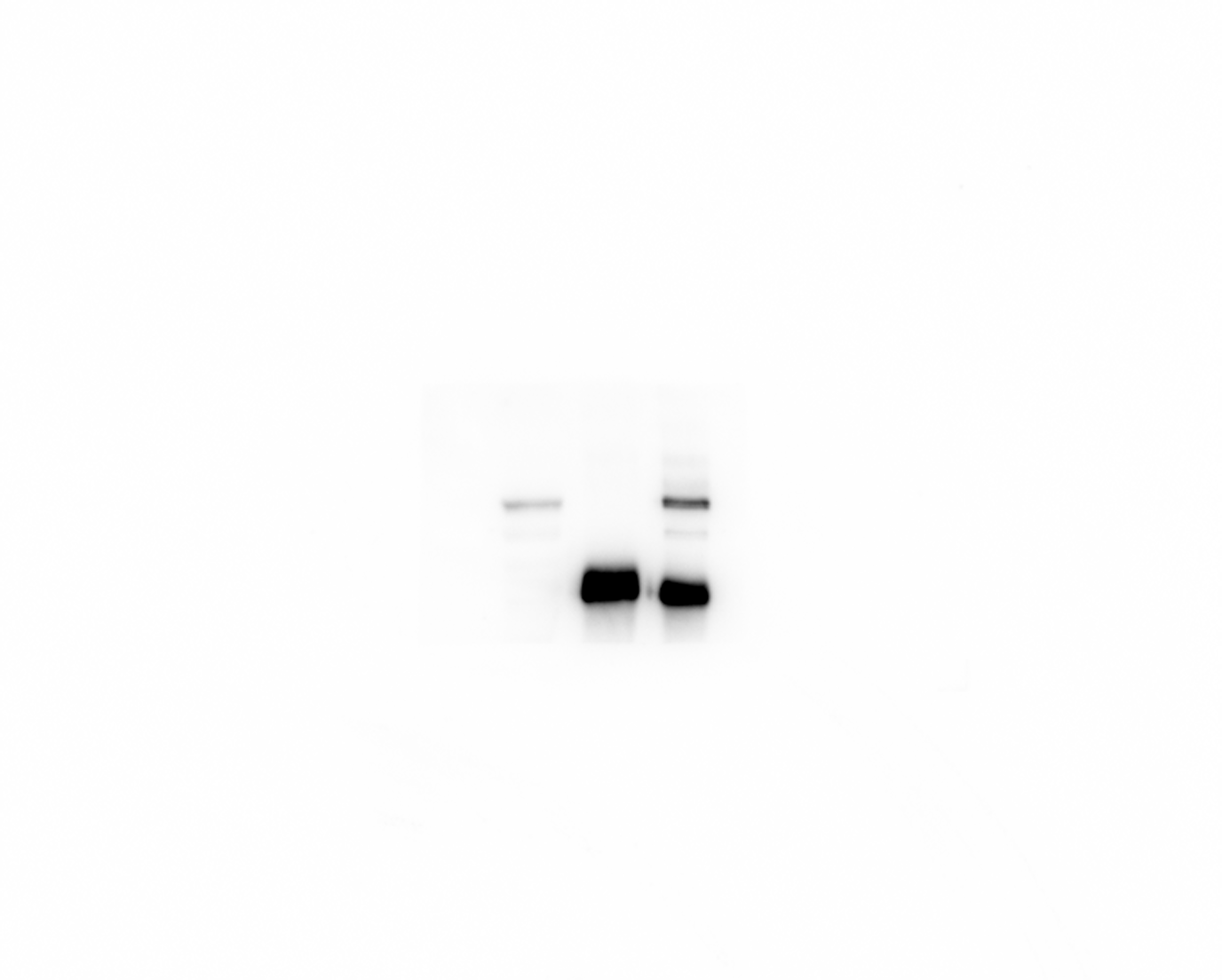

Supplement: Figure 3—source data 3. [file elife-73252-fig3-data3.zip › Figure 3-source data 3/Unlabeled immunoblots/TA-IP-TA-RPE1-Figure 3A.Tif]

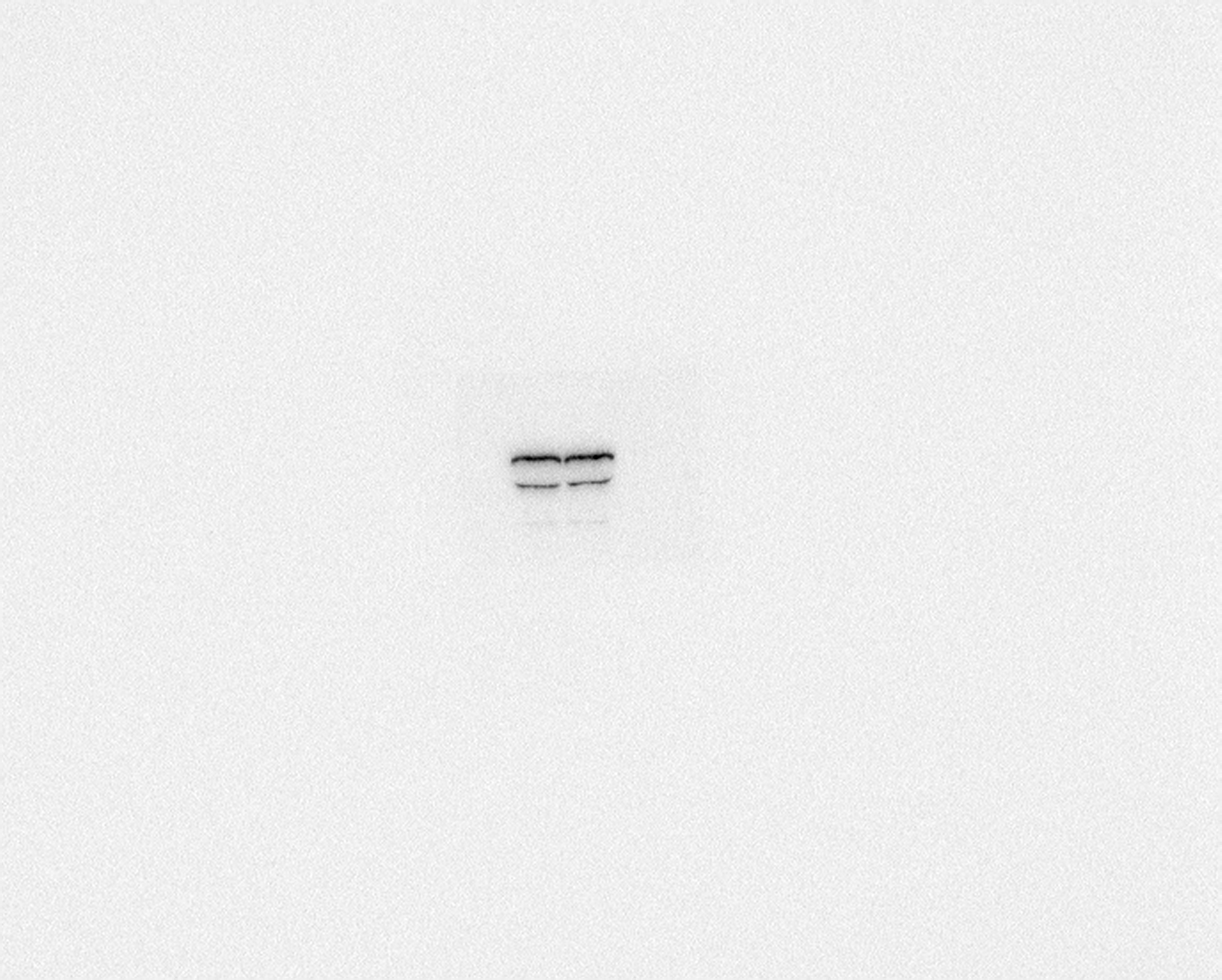

Supplement: Figure 3—source data 3. [file elife-73252-fig3-data3.zip › Figure 3-source data 3/Unlabeled immunoblots/TG-30s-Figure 3E.Tif]

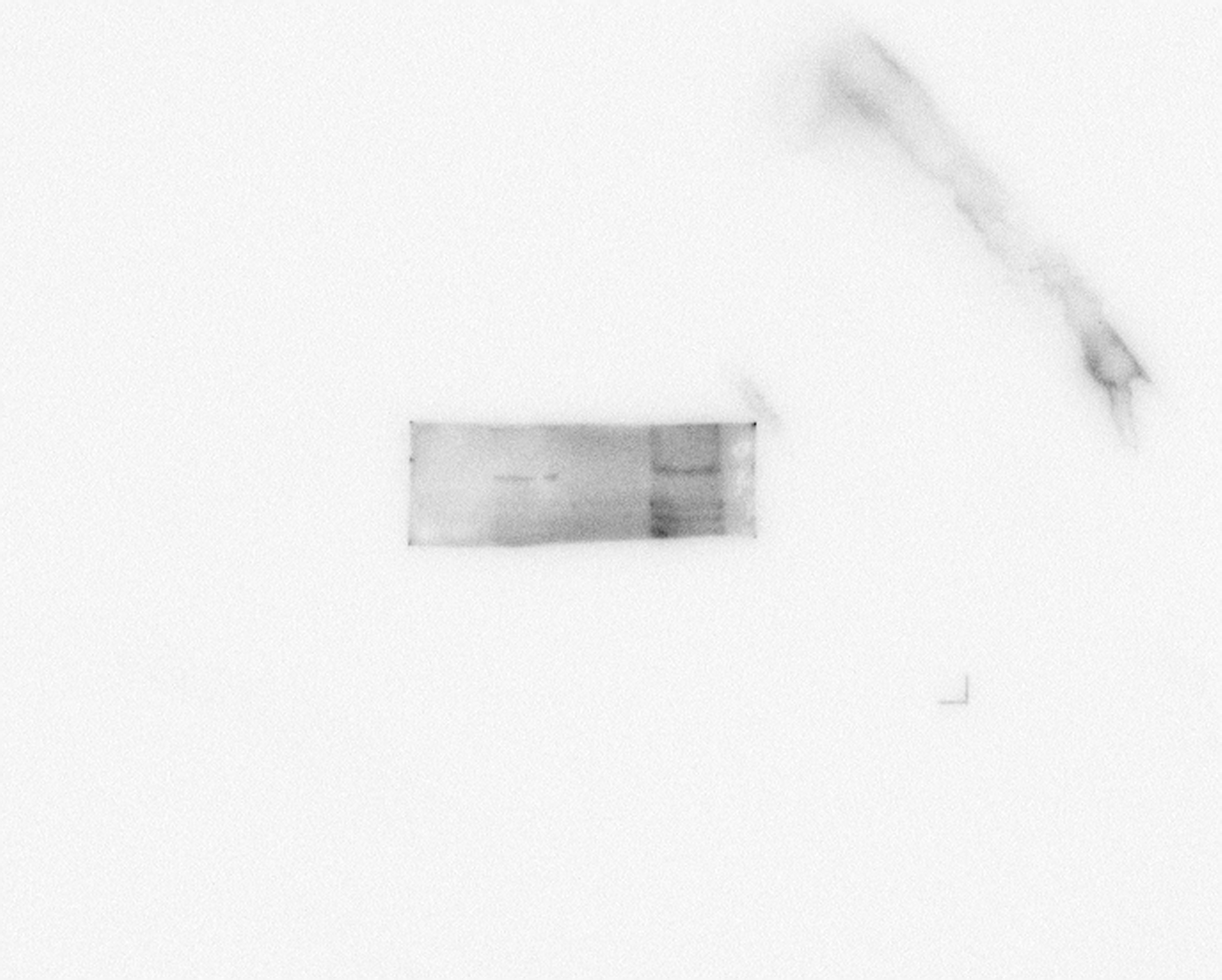

Supplement: Figure 3—source data 3. [file elife-73252-fig3-data3.zip › Figure 3-source data 3/Unlabeled immunoblots/TG-IP-ODF2-40s-Figure 3B.Tif]

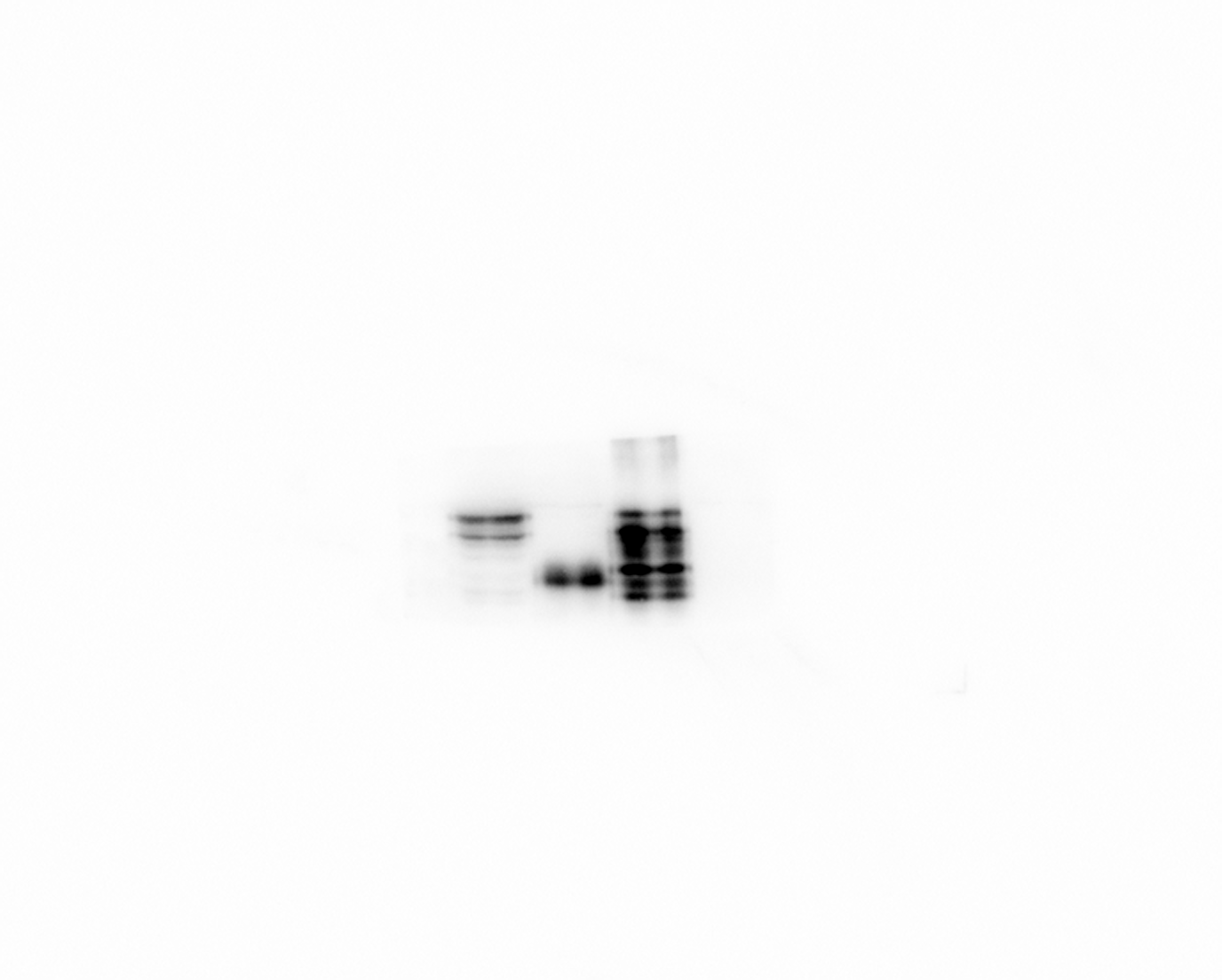

Supplement: Figure 3—source data 3. [file elife-73252-fig3-data3.zip › Figure 3-source data 3/Unlabeled immunoblots/TG-IP-TG-30s-Figure 3B.Tif]

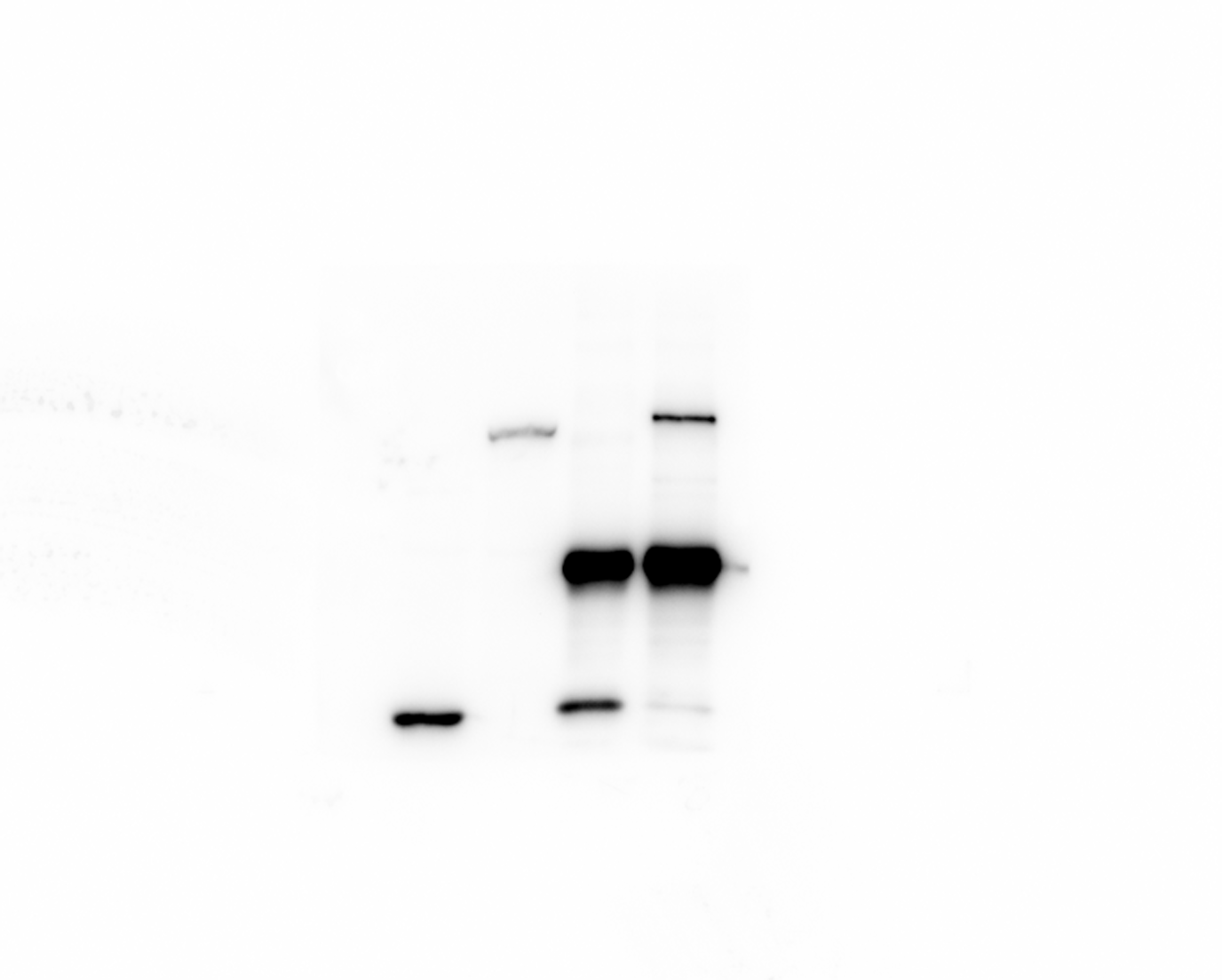

Supplement: Figure 3—figure supplement 1—source data 7. [file elife-73252-fig3-figsupp1-data7.zip › Figure 3-figure supplement 1-source data 7/Unlabled immunoblots/CCDC120-GFP-IP-GFP-Figure 3-figure supplement 1H.Tif]

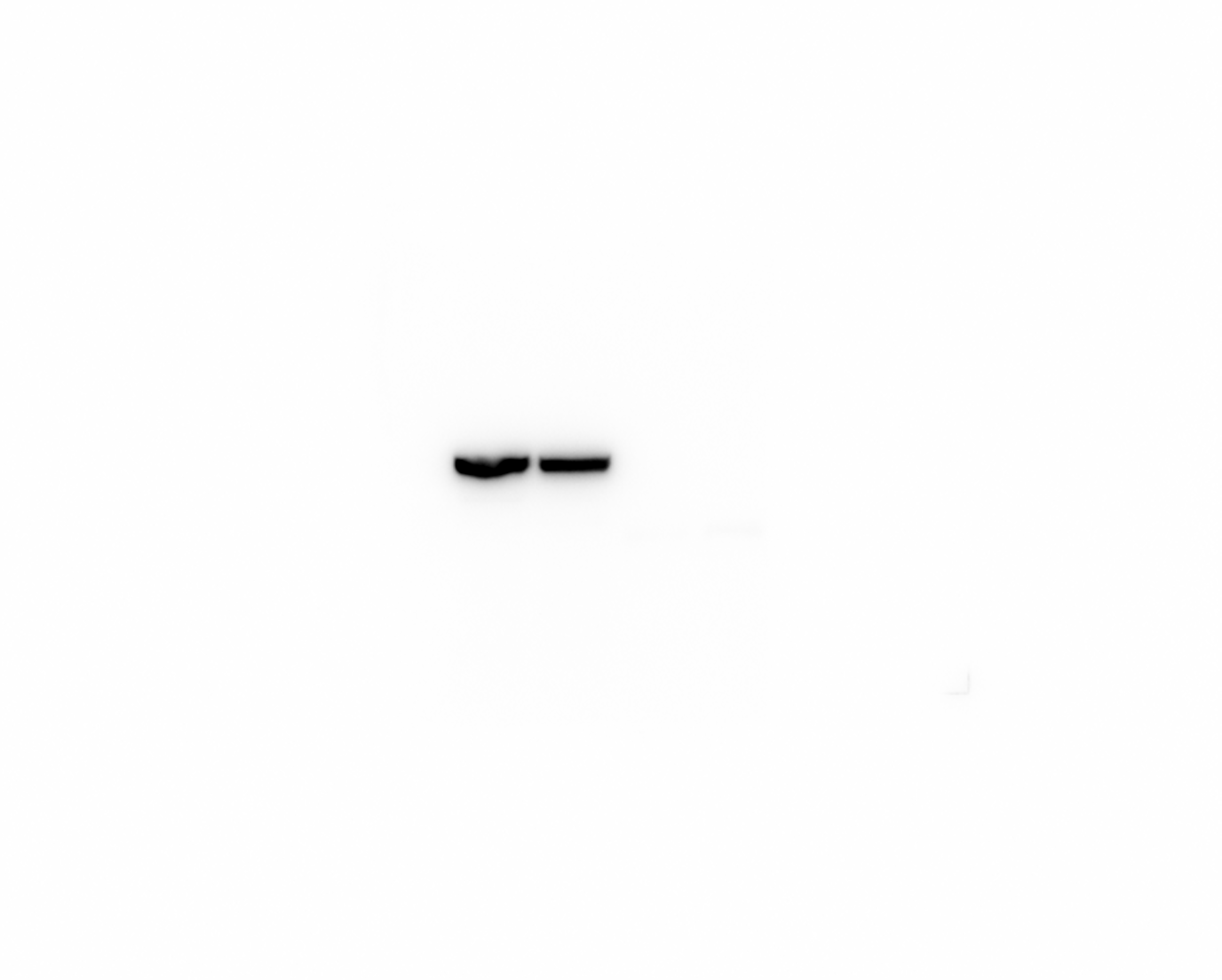

Supplement: Figure 3—figure supplement 1—source data 7. [file elife-73252-fig3-figsupp1-data7.zip › Figure 3-figure supplement 1-source data 7/Unlabled immunoblots/CCDC120-GFP-IP-HA-Figure 3-figure supplement 1H.Tif]

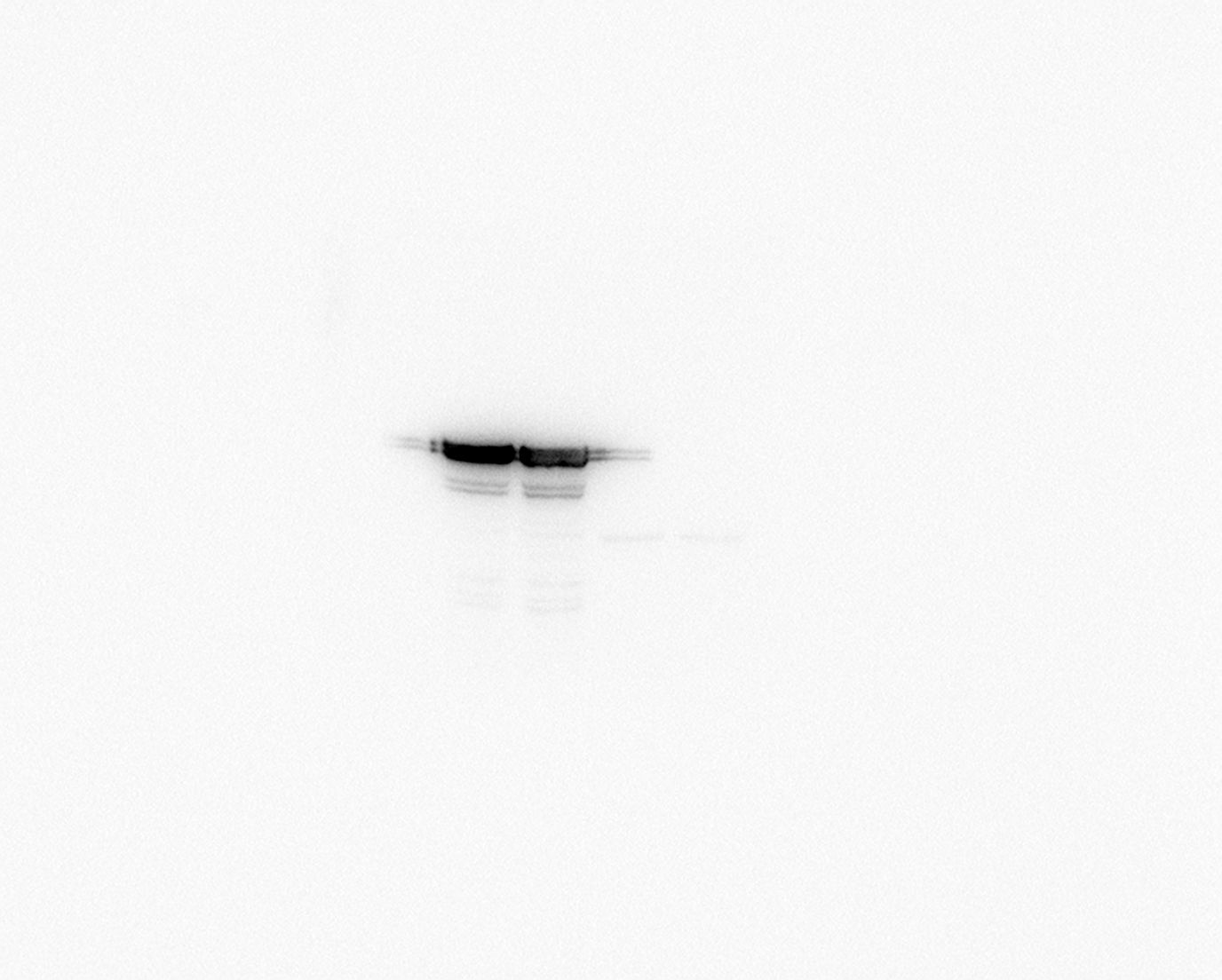

Supplement: Figure 3—figure supplement 1—source data 7. [file elife-73252-fig3-figsupp1-data7.zip › Figure 3-figure supplement 1-source data 7/Unlabled immunoblots/CCDC68-V5-Input-IP-HA-Figure 3-figure supplement 1G.Tif]

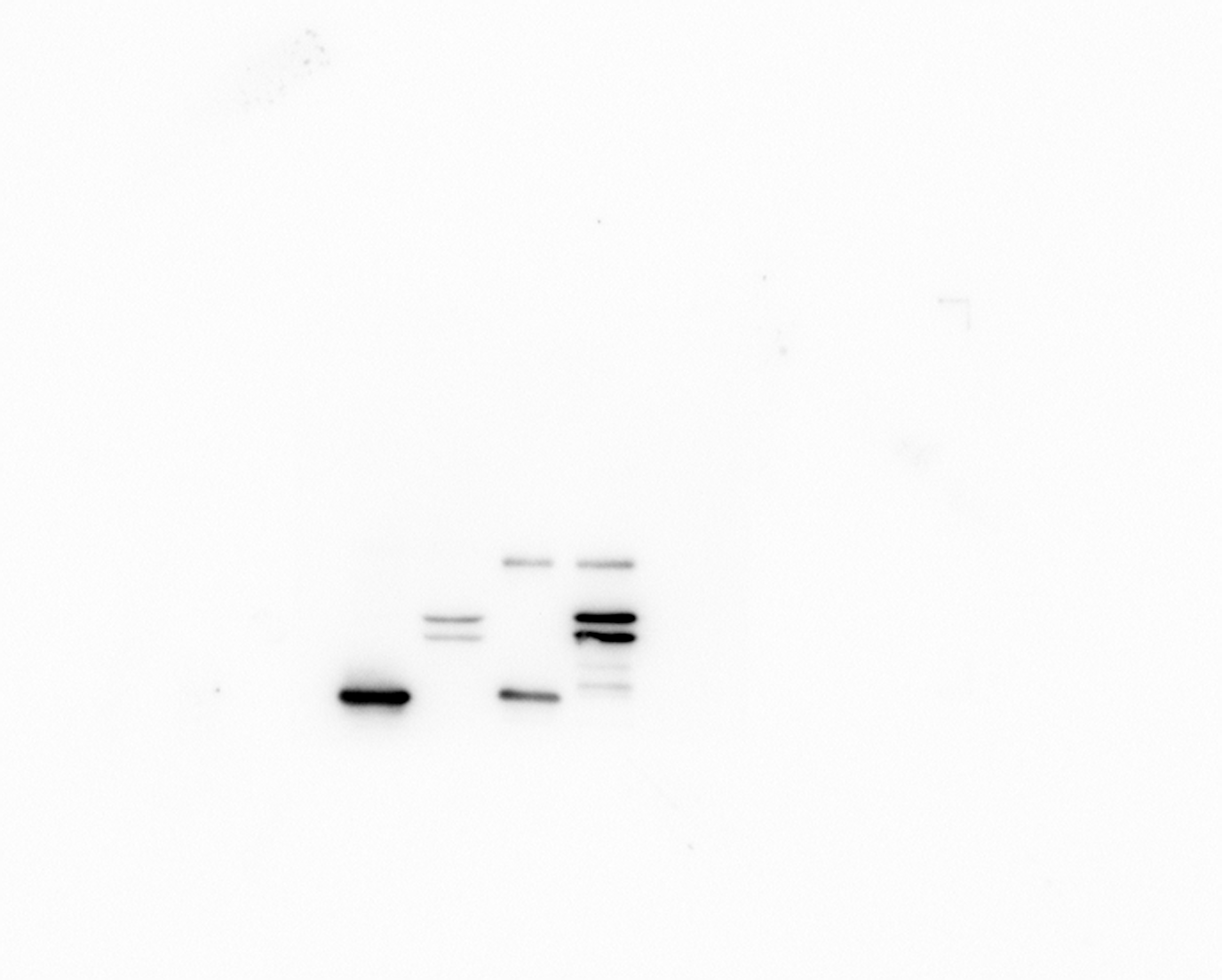

Supplement: Figure 3—figure supplement 1—source data 7. [file elife-73252-fig3-figsupp1-data7.zip › Figure 3-figure supplement 1-source data 7/Unlabled immunoblots/CCDC68-V5-IP-V5-Figure 3-Supplement 1G.Tif]

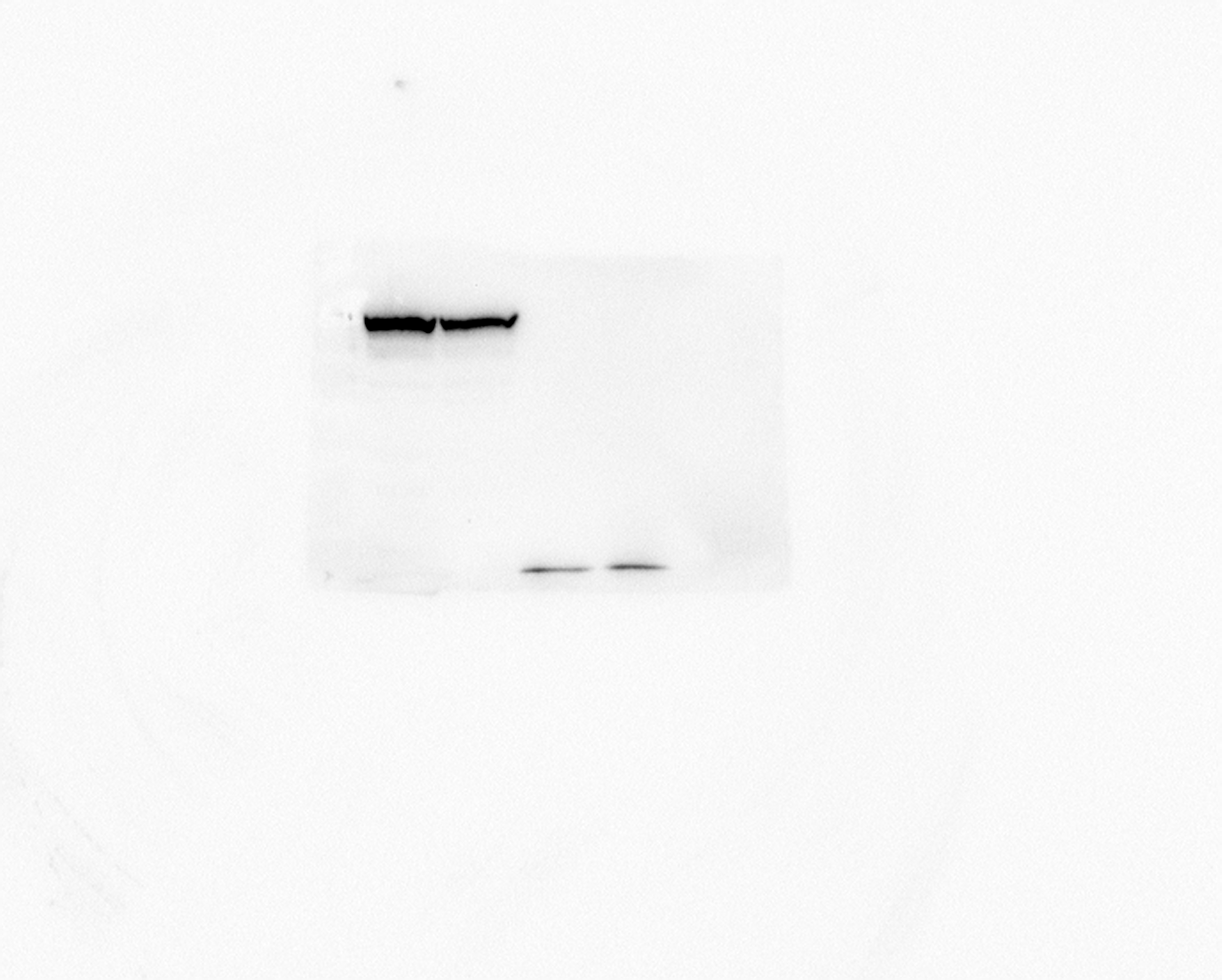

Supplement: Figure 3—figure supplement 1—source data 7. [file elife-73252-fig3-figsupp1-data7.zip › Figure 3-figure supplement 1-source data 7/Unlabled immunoblots/Flag-TG-IP-CCDC120-GFP-Figure 3-figure supplement 1J.Tif]

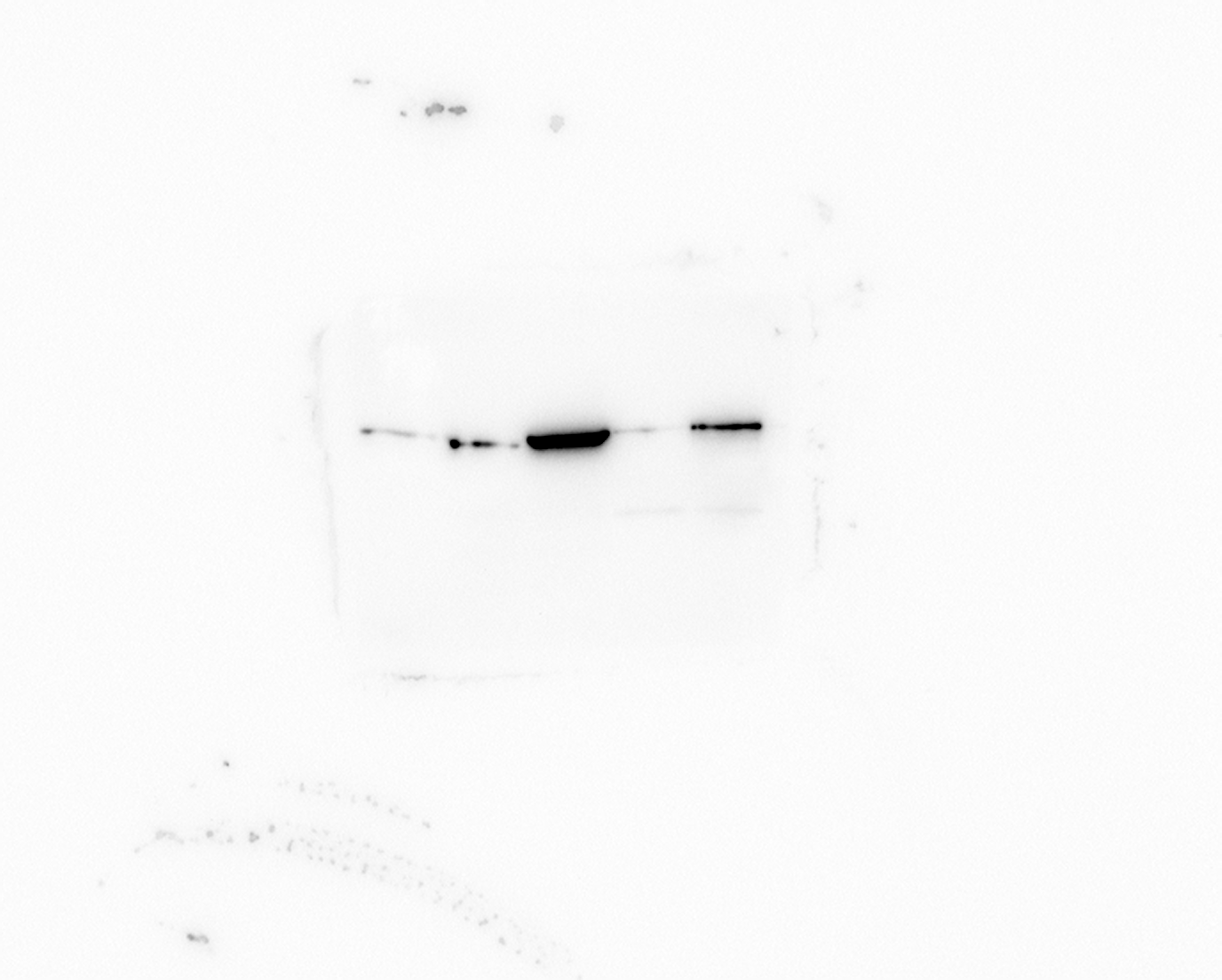

Supplement: Figure 3—figure supplement 1—source data 7. [file elife-73252-fig3-figsupp1-data7.zip › Figure 3-figure supplement 1-source data 7/Unlabled immunoblots/Flag-TG-IP-Flag3-Figure 3-figure supplement 1J.Tif]

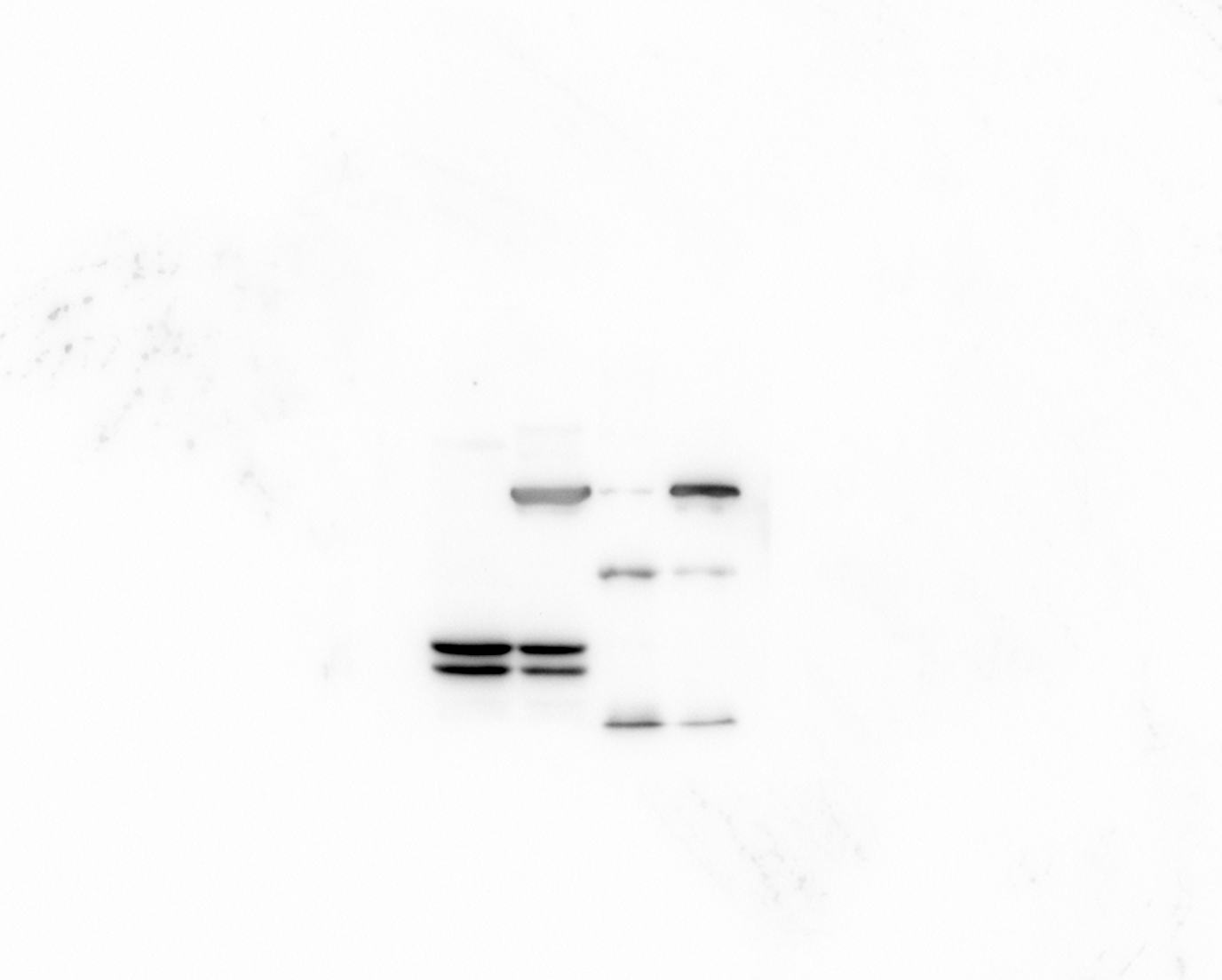

Supplement: Figure 3—figure supplement 1—source data 7. [file elife-73252-fig3-figsupp1-data7.zip › Figure 3-figure supplement 1-source data 7/Unlabled immunoblots/Input-IP-CCDCSIX-EIGHT2-Figure 3-figure supplement 1I.Tif]

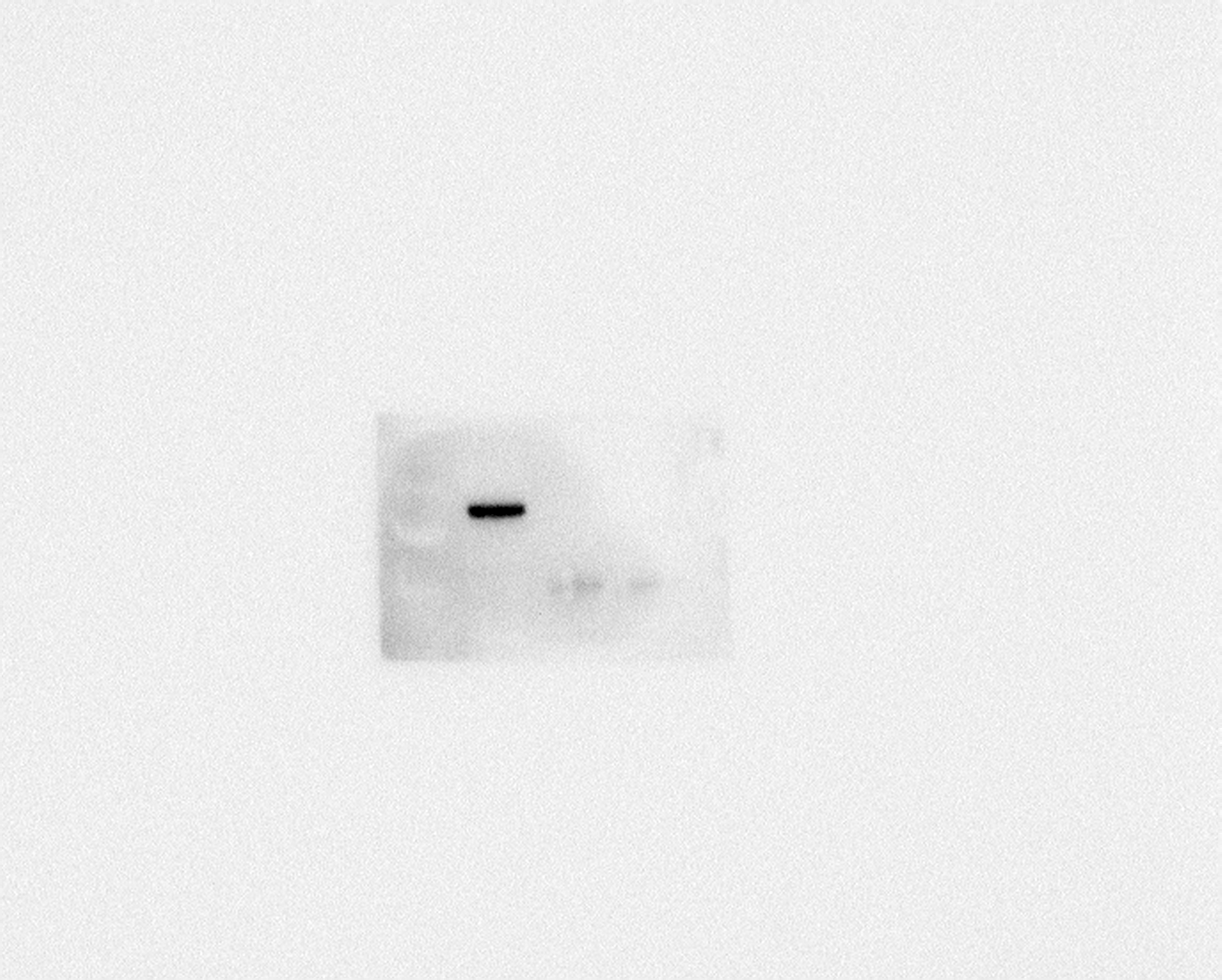

Supplement: Figure 3—figure supplement 1—source data 7. [file elife-73252-fig3-figsupp1-data7.zip › Figure 3-figure supplement 1-source data 7/Unlabled immunoblots/IP-TA-Figure 3-figure supplement 1k.Tif]

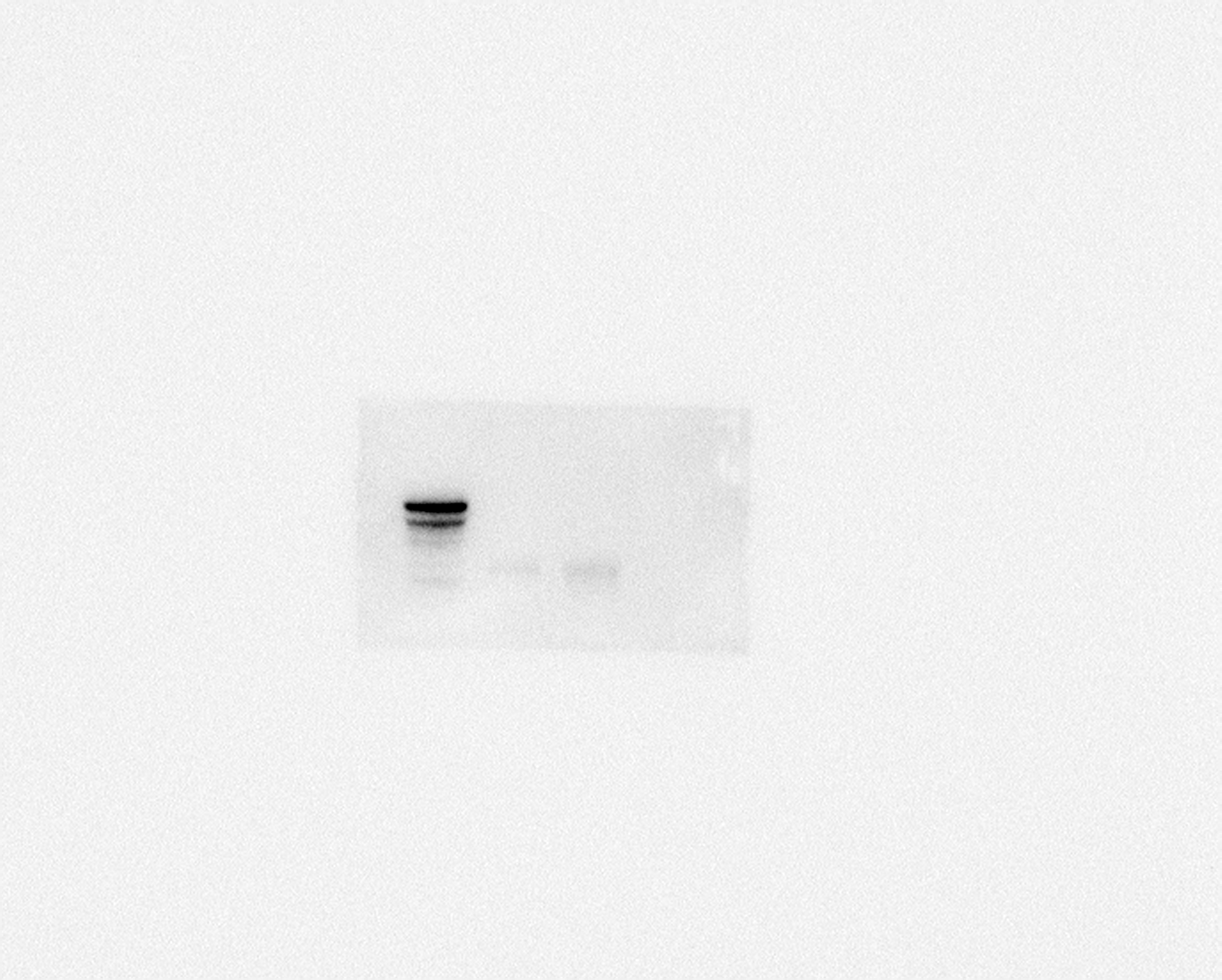

Supplement: Figure 3—figure supplement 1—source data 7. [file elife-73252-fig3-figsupp1-data7.zip › Figure 3-figure supplement 1-source data 7/Unlabled immunoblots/IP-TG-Figure 3-figure supplement 1K.Tif]

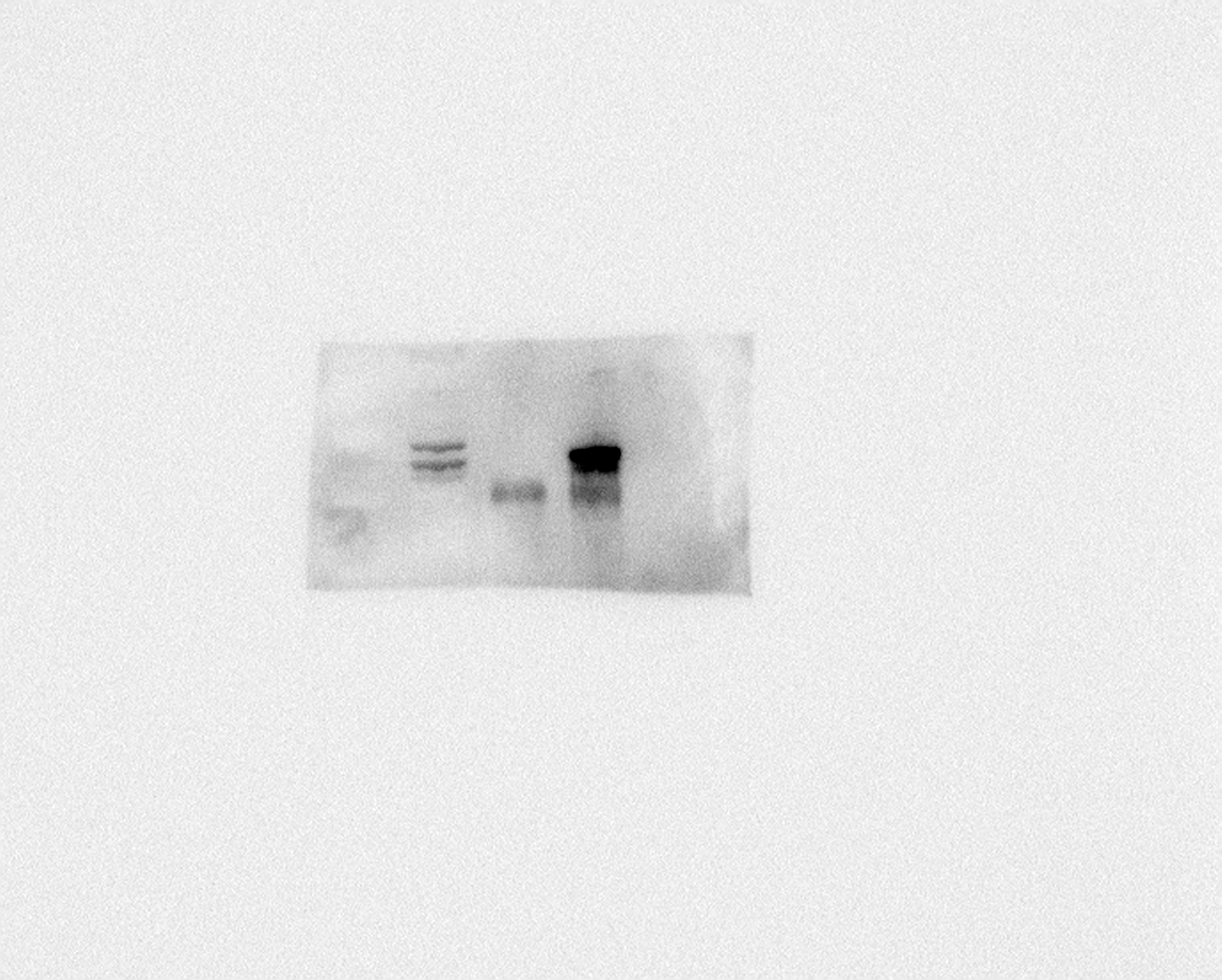

Supplement: Figure 3—figure supplement 1—source data 7. [file elife-73252-fig3-figsupp1-data7.zip › Figure 3-figure supplement 1-source data 7/Unlabled immunoblots/TCHP-2-Figure 3-figure supplement 1K.Tif]

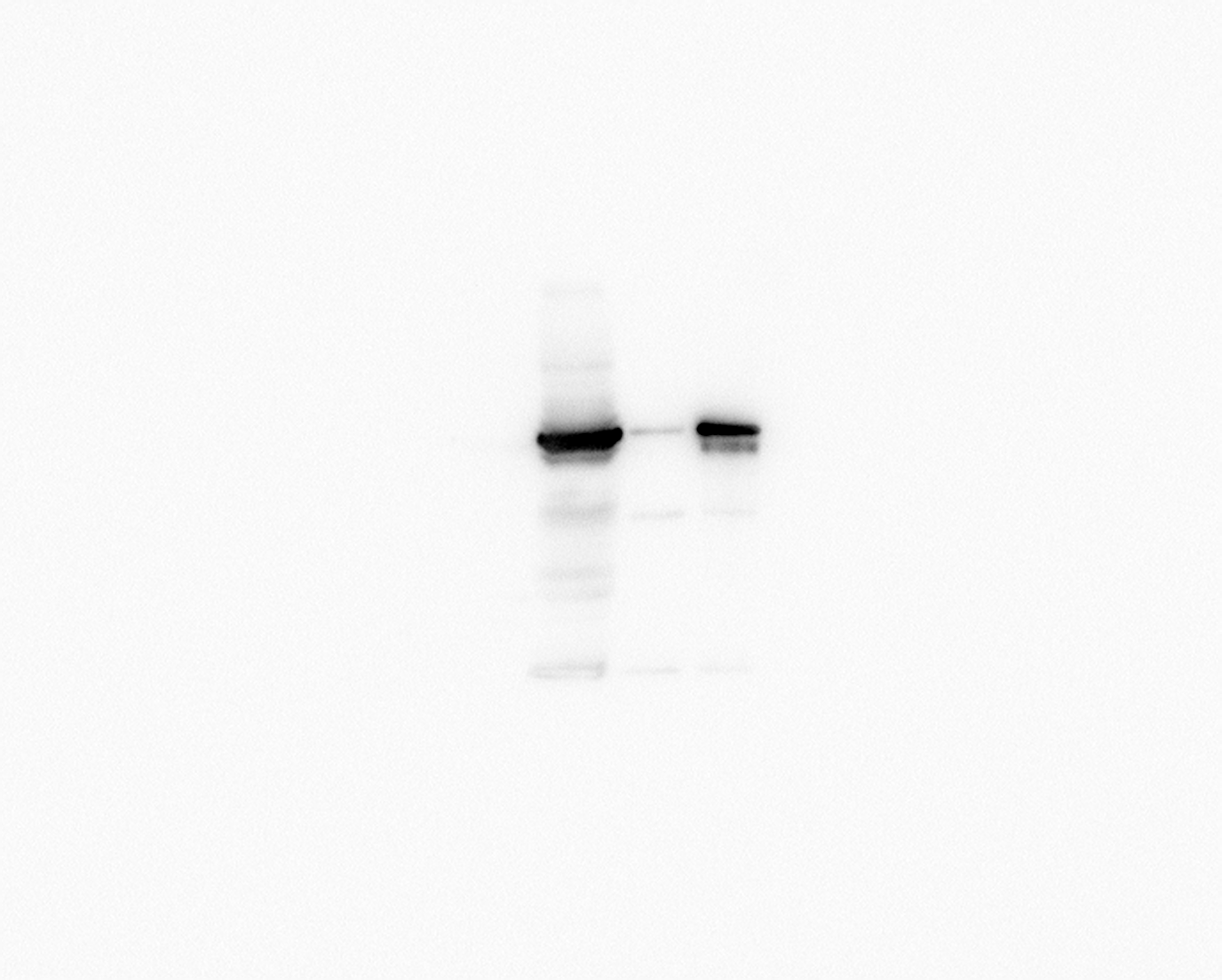

Supplement: Figure 3—figure supplement 1—source data 7. [file elife-73252-fig3-figsupp1-data7.zip › Figure 3-figure supplement 1-source data 7/Unlabled immunoblots/TXLNGInput-Flag-Figure 3-figure supplement 1I.Tif]

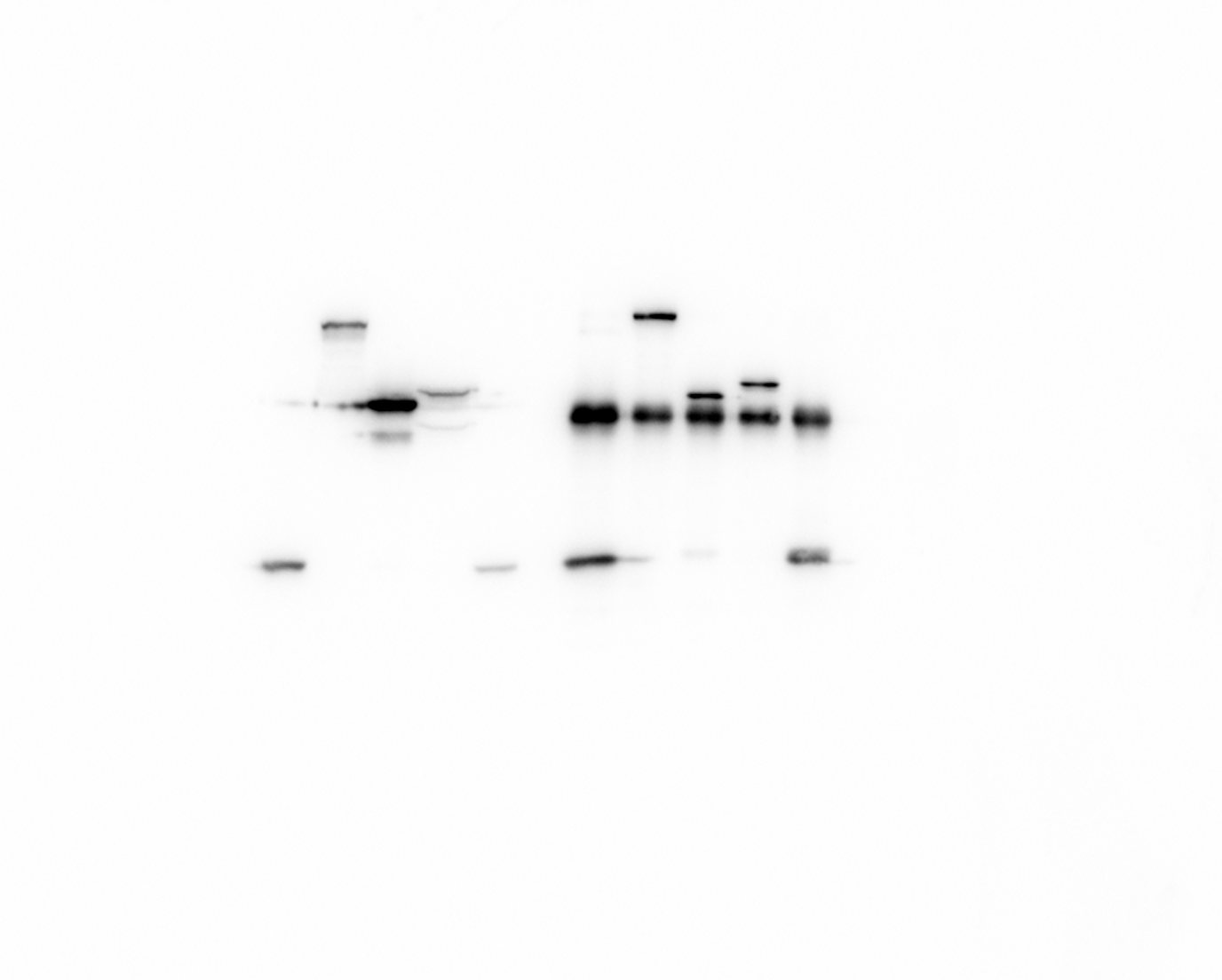

Supplement: Figure 4—source data 3. [file elife-73252-fig4-data3.zip › Figure 4-source data 3/unlabeled immublots for Figure 4/1-Input-TA-GFP-IP-2-Figure 4F.Tif]

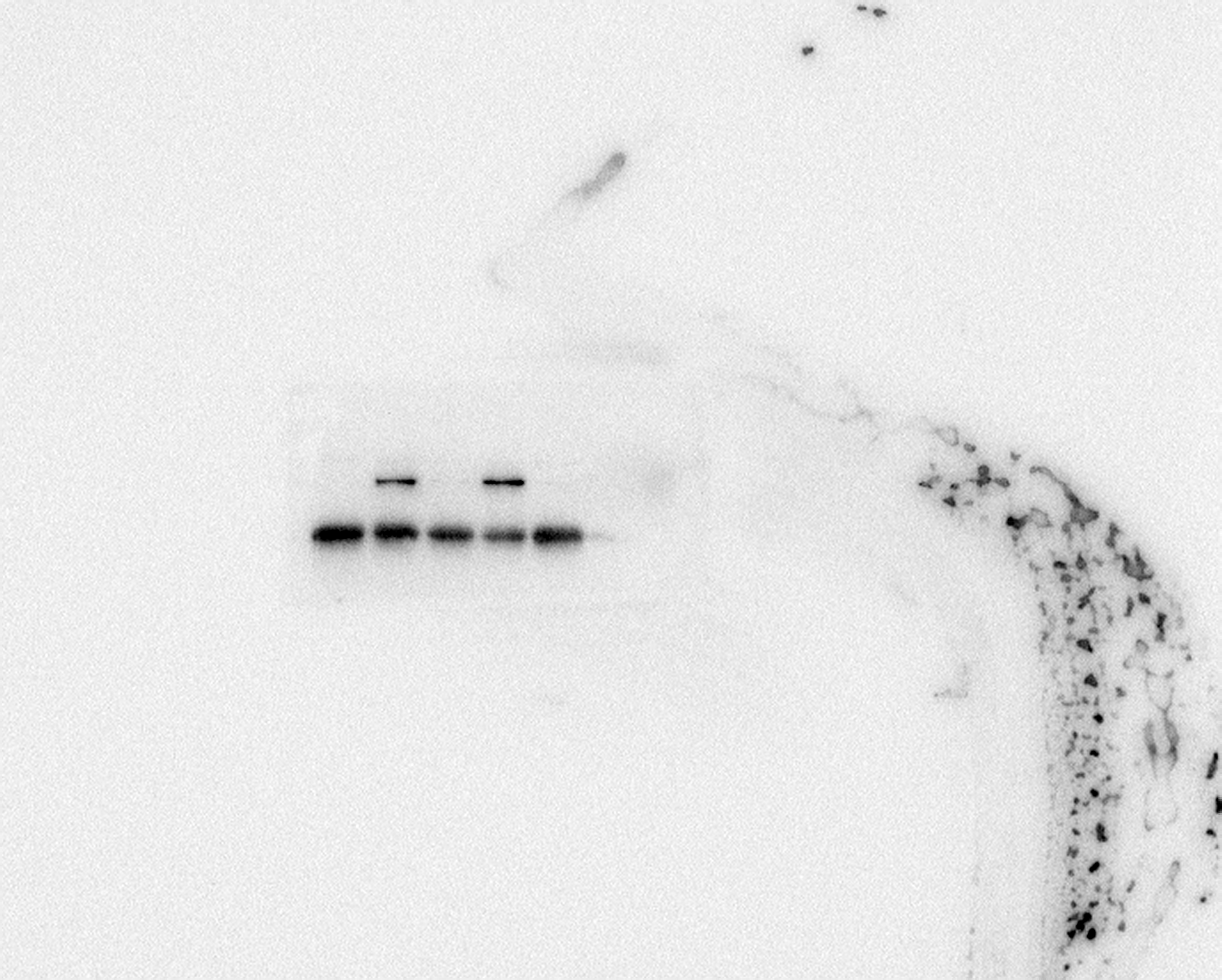

Supplement: Figure 4—source data 3. [file elife-73252-fig4-data3.zip › Figure 4-source data 3/unlabeled immublots for Figure 4/2-Input-Flag-TG-2-Figure 4F.Tif]

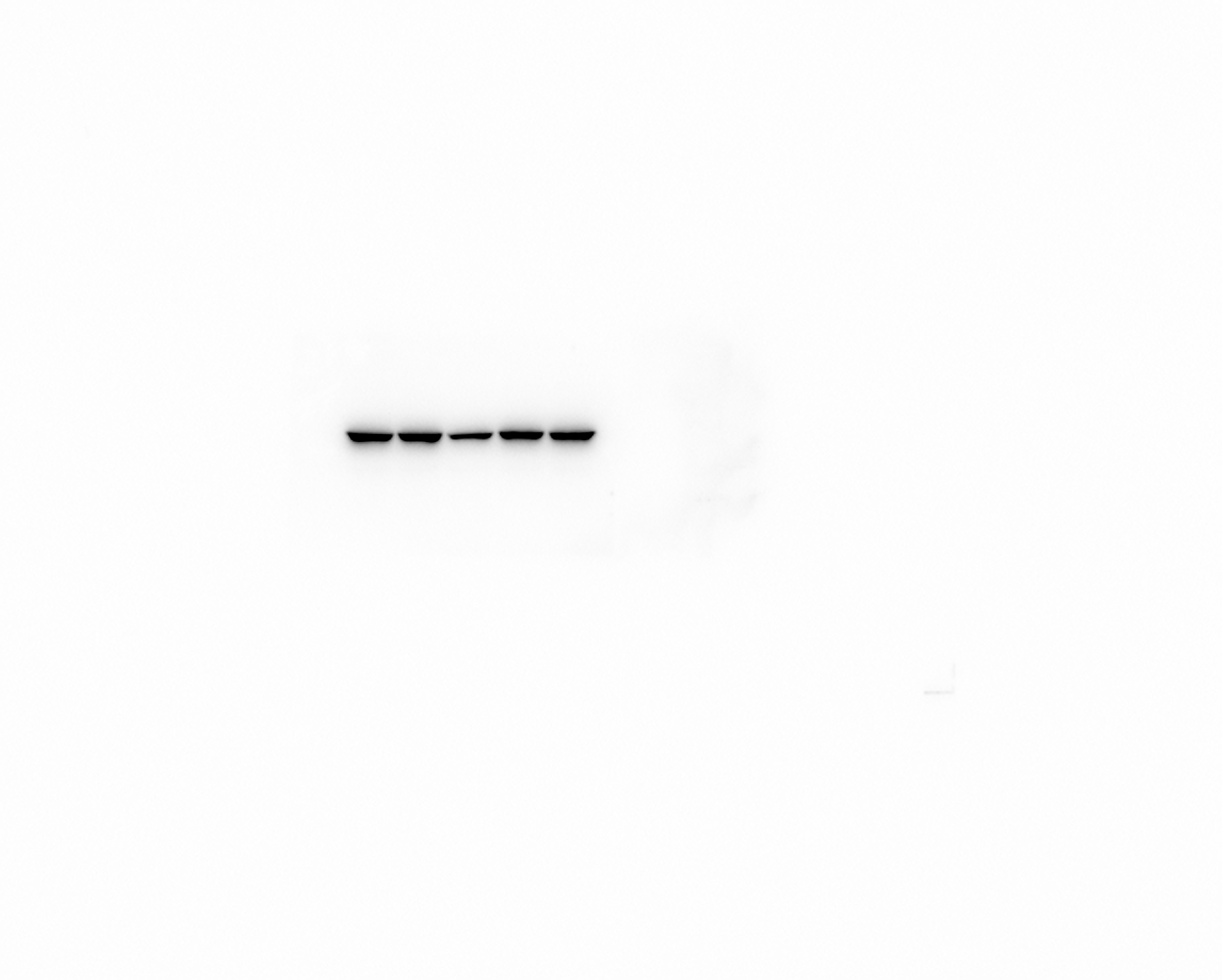

Supplement: Figure 4—source data 3. [file elife-73252-fig4-data3.zip › Figure 4-source data 3/unlabeled immublots for Figure 4/2-Input-Flag-TG-Figure 4F.Tif]

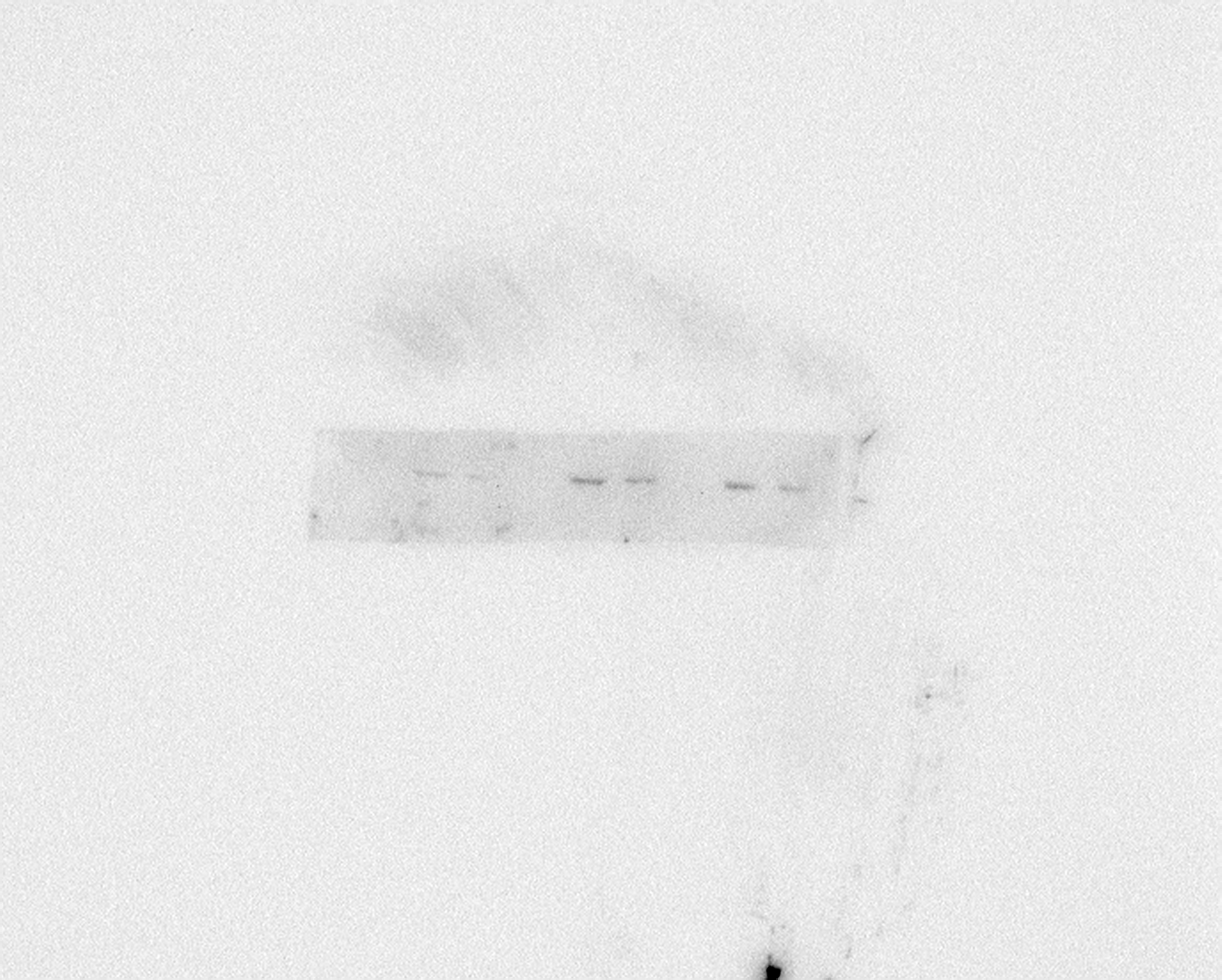

Supplement: Figure 4—source data 3. [file elife-73252-fig4-data3.zip › Figure 4-source data 3/unlabeled immublots for Figure 4/Actin-Figure 4B.Tif]

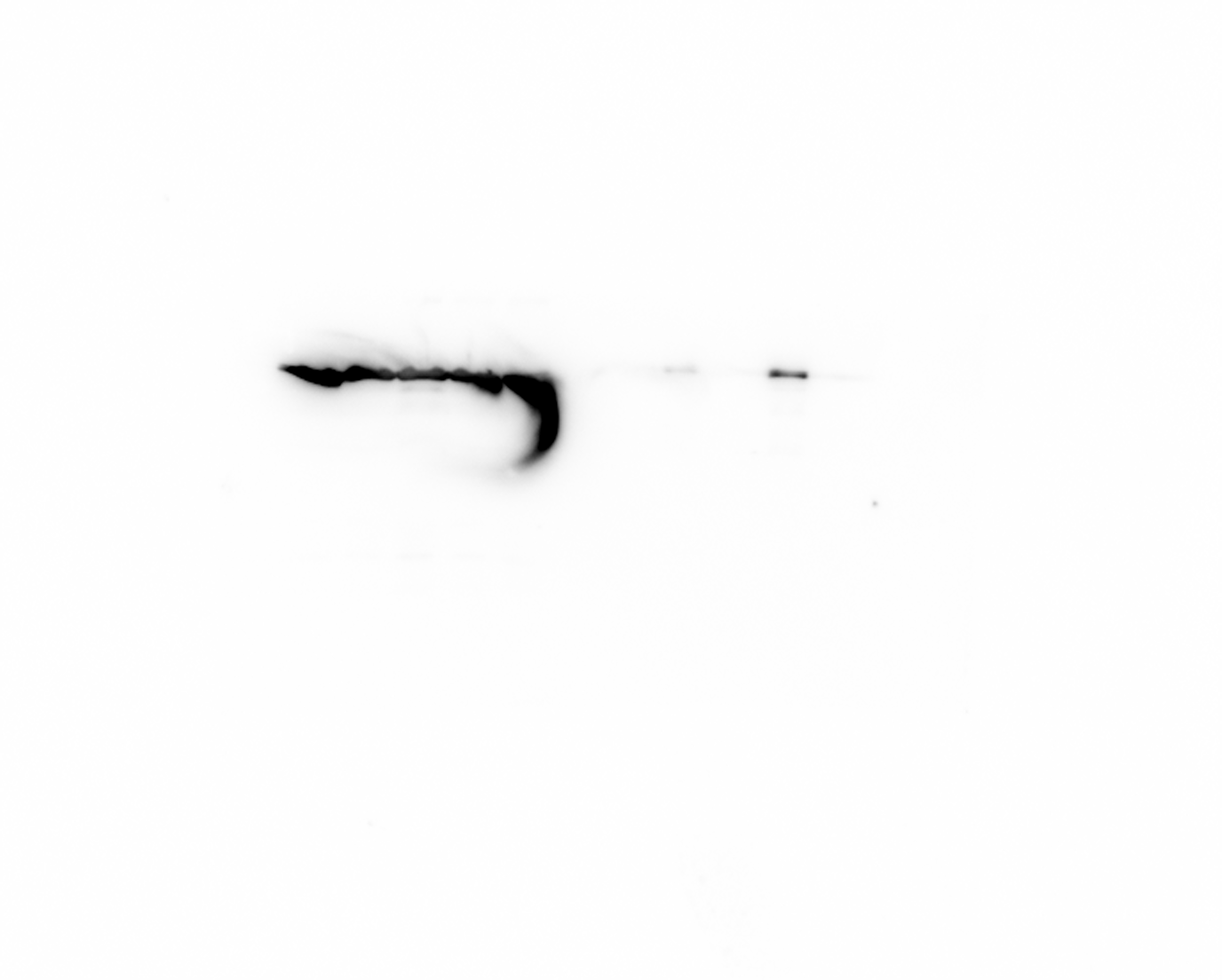

Supplement: Figure 4—source data 3. [file elife-73252-fig4-data3.zip › Figure 4-source data 3/unlabeled immublots for Figure 4/Flag-TXLNA-Input-IP-Figure 4H.Tif]

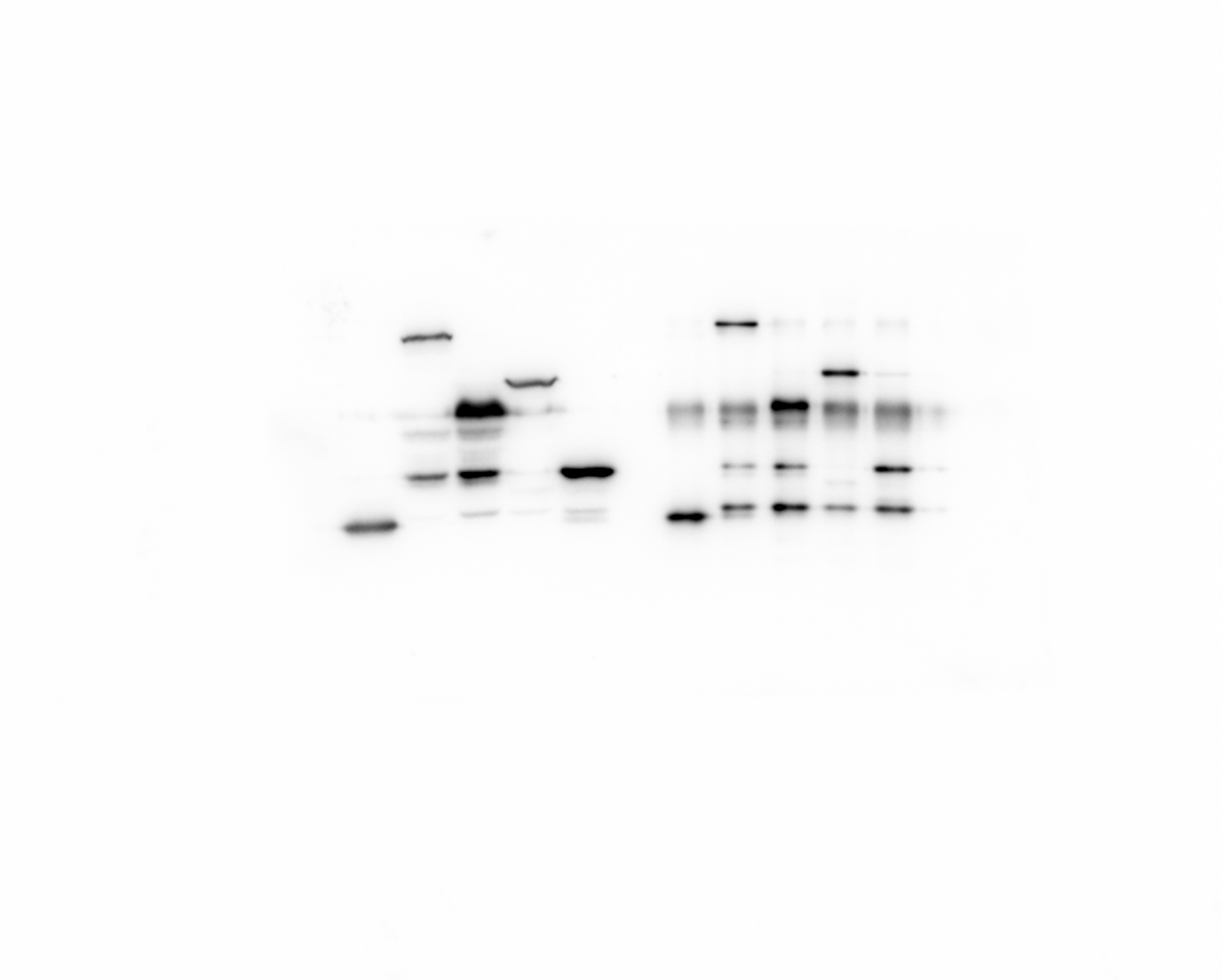

Supplement: Figure 4—source data 3. [file elife-73252-fig4-data3.zip › Figure 4-source data 3/unlabeled immublots for Figure 4/GFP-TXLNG-Input-IP-Figure 4H.Tif]

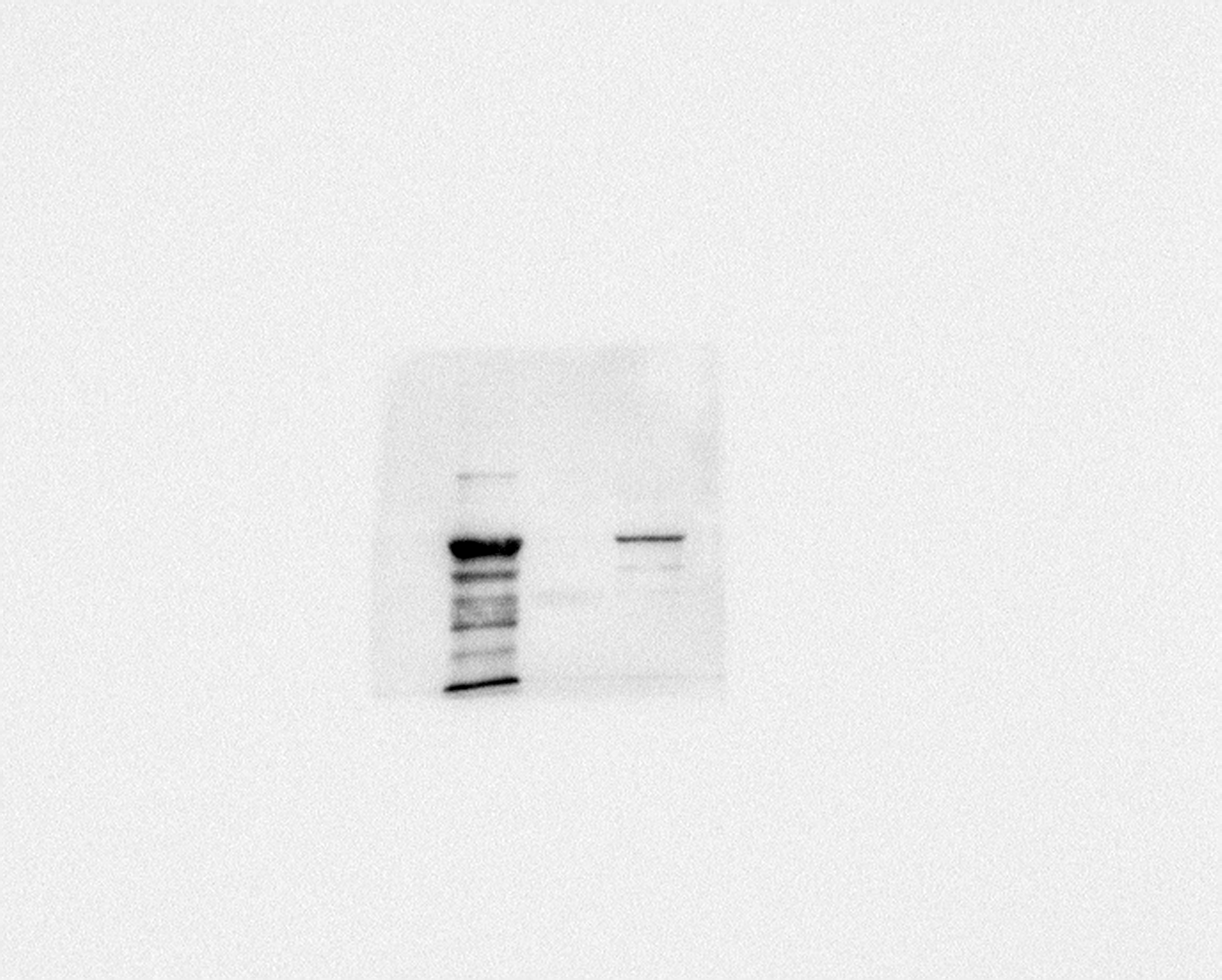

Supplement: Figure 4—source data 3. [file elife-73252-fig4-data3.zip › Figure 4-source data 3/unlabeled immublots for Figure 4/mbp-tgm-tam-gst2-Figure 4I.Tif]

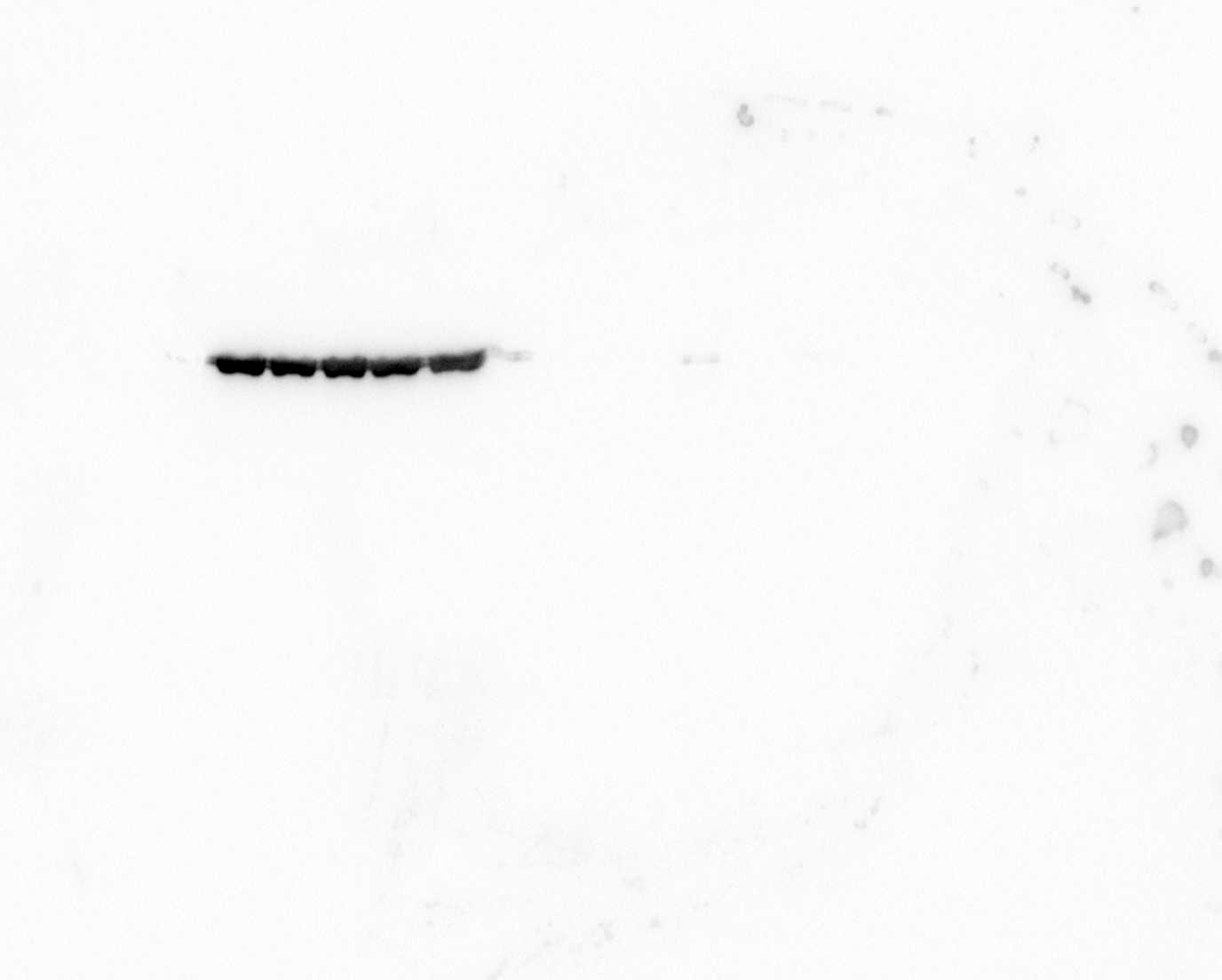

Supplement: Figure 4—source data 3. [file elife-73252-fig4-data3.zip › Figure 4-source data 3/unlabeled immublots for Figure 4/RE-Flag-TXLNA-Input-IP2-Figure 4H.Tif]

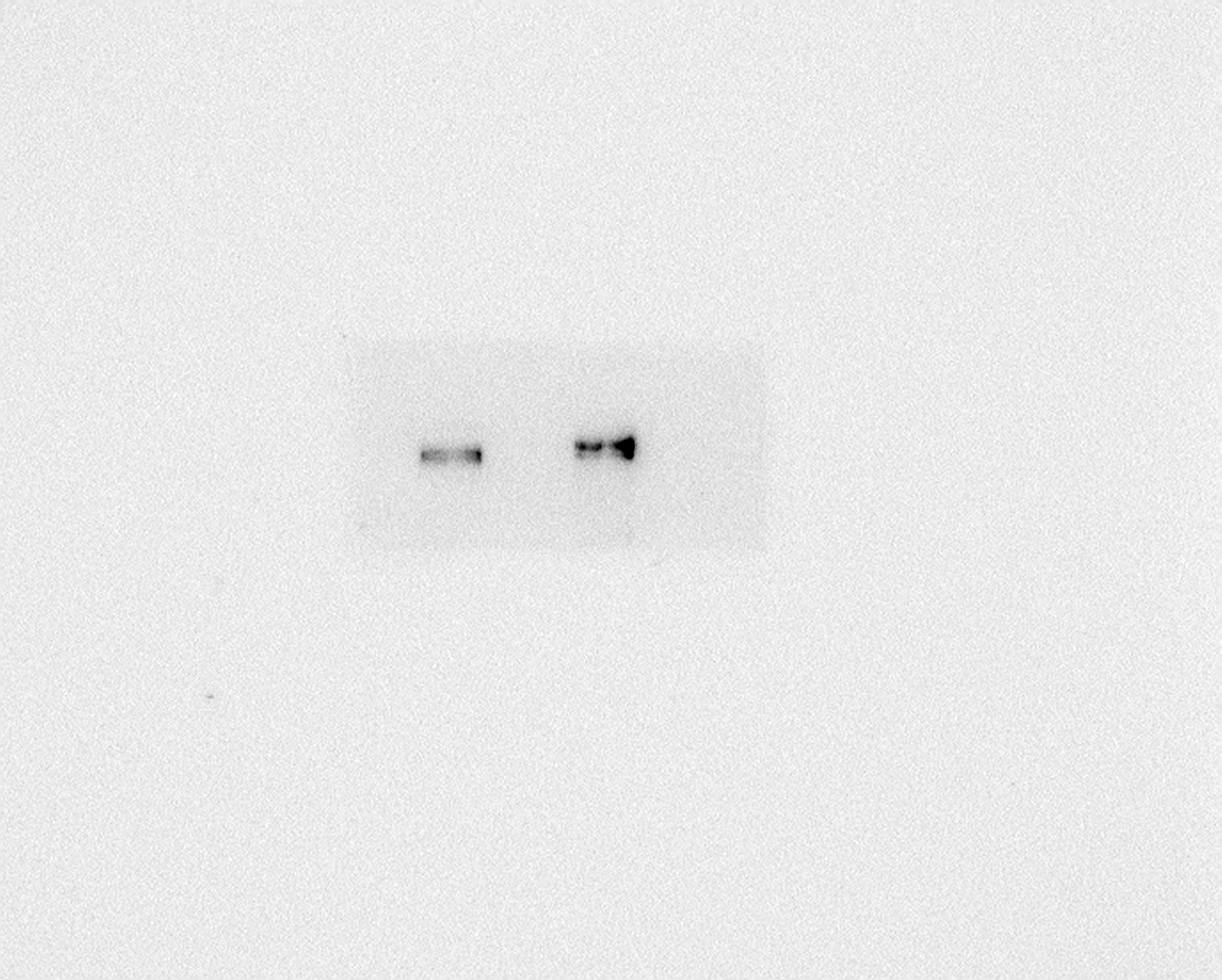

Supplement: Figure 4—source data 3. [file elife-73252-fig4-data3.zip › Figure 4-source data 3/unlabeled immublots for Figure 4/TA-IP-CEP170-2-Figure 4A.Tif]

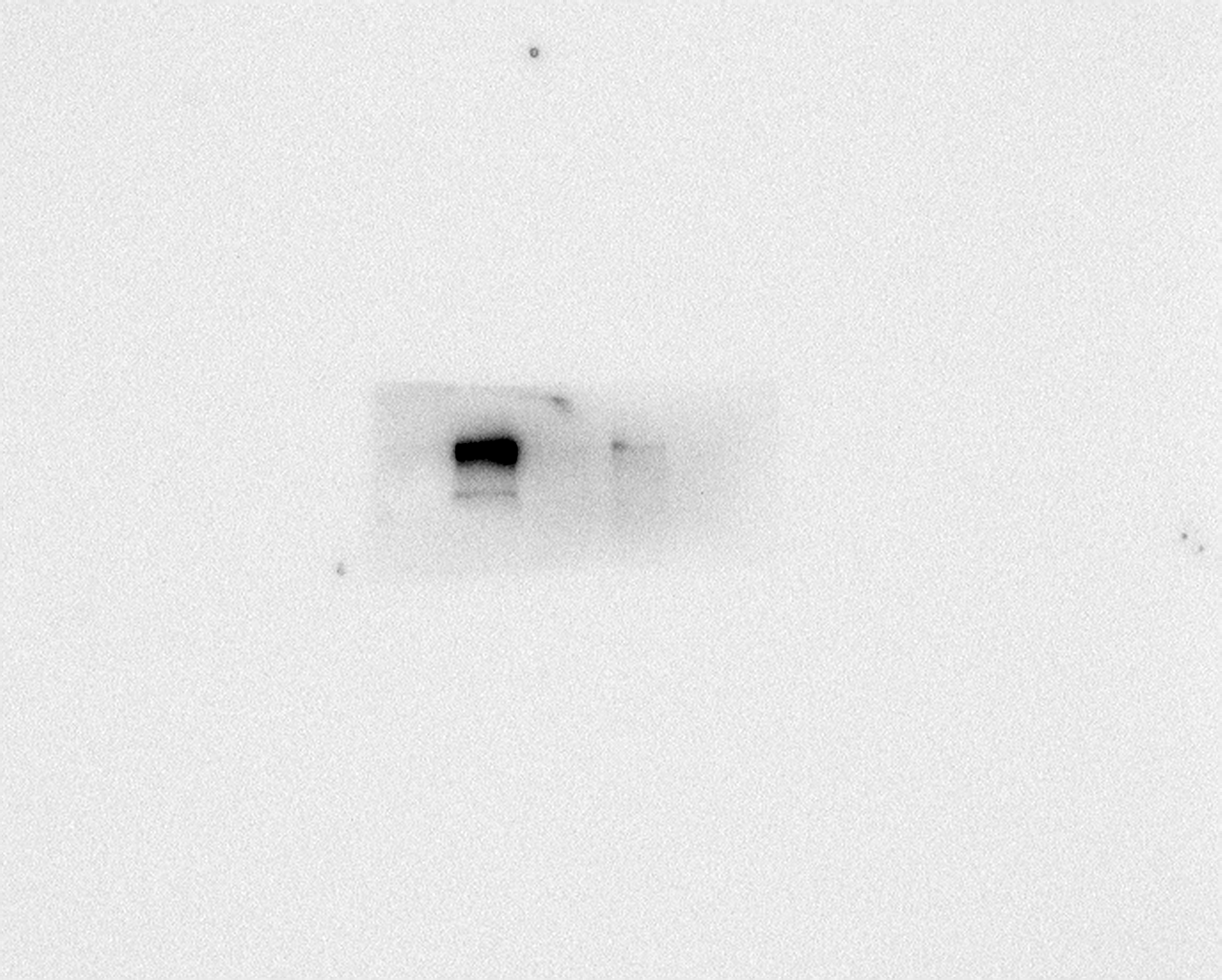

Supplement: Figure 4—source data 3. [file elife-73252-fig4-data3.zip › Figure 4-source data 3/unlabeled immublots for Figure 4/TA-IP-NiN1'-Figure 4A.Tif]

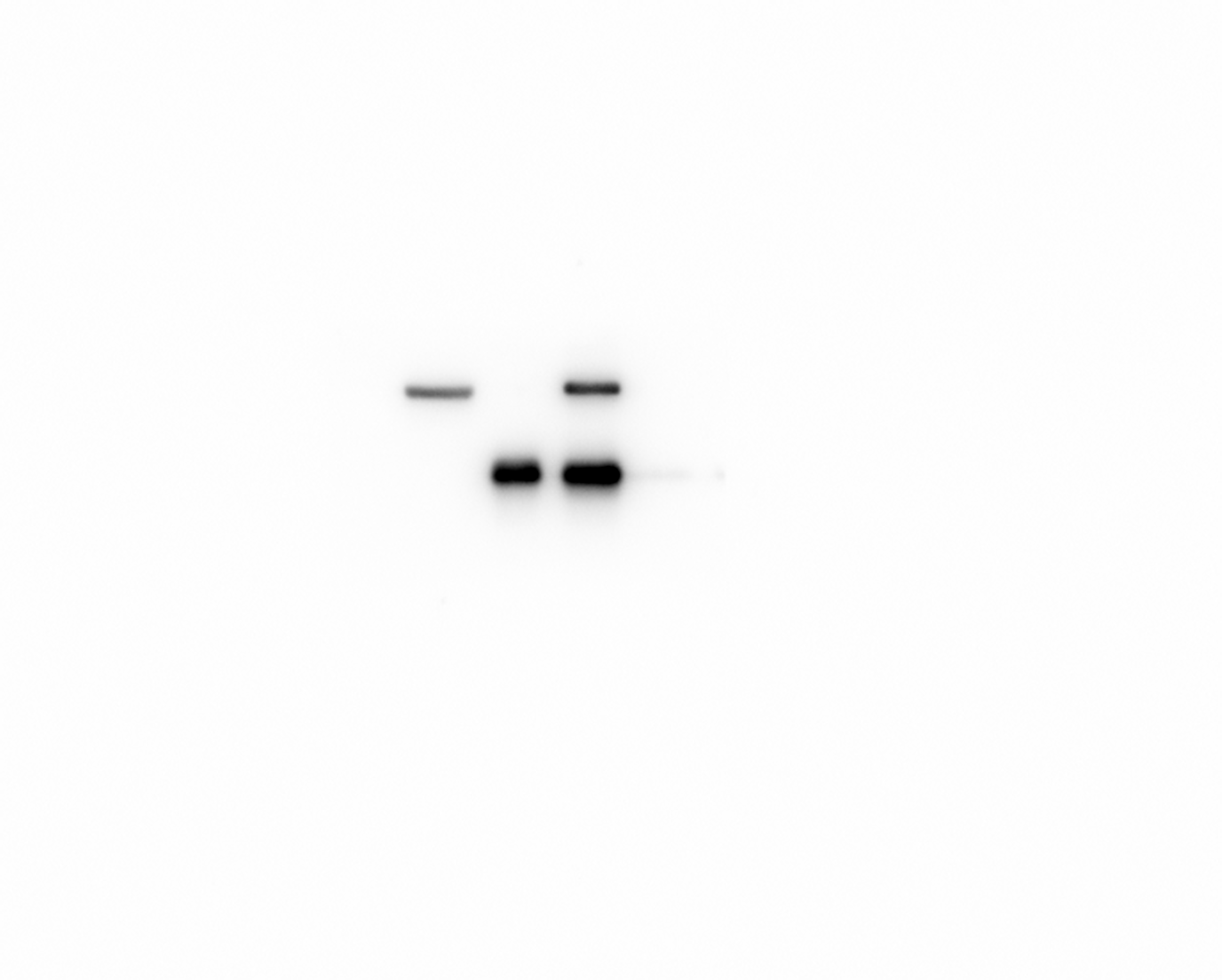

Supplement: Figure 4—source data 3. [file elife-73252-fig4-data3.zip › Figure 4-source data 3/unlabeled immublots for Figure 4/TA-IP-TA-Figure 4A.Tif]

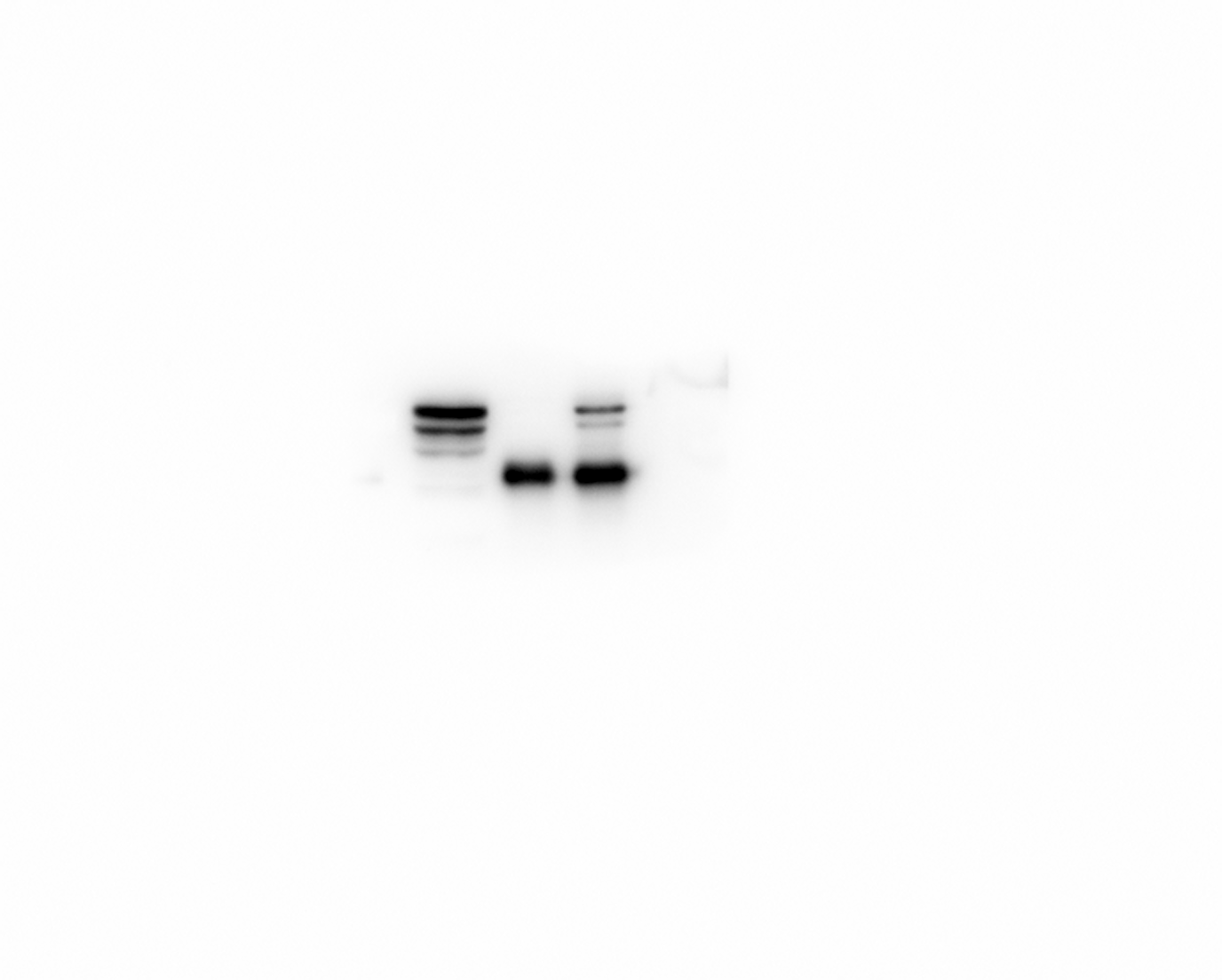

Supplement: Figure 4—source data 3. [file elife-73252-fig4-data3.zip › Figure 4-source data 3/unlabeled immublots for Figure 4/TA-IP-TG-Figure 4A.Tif]

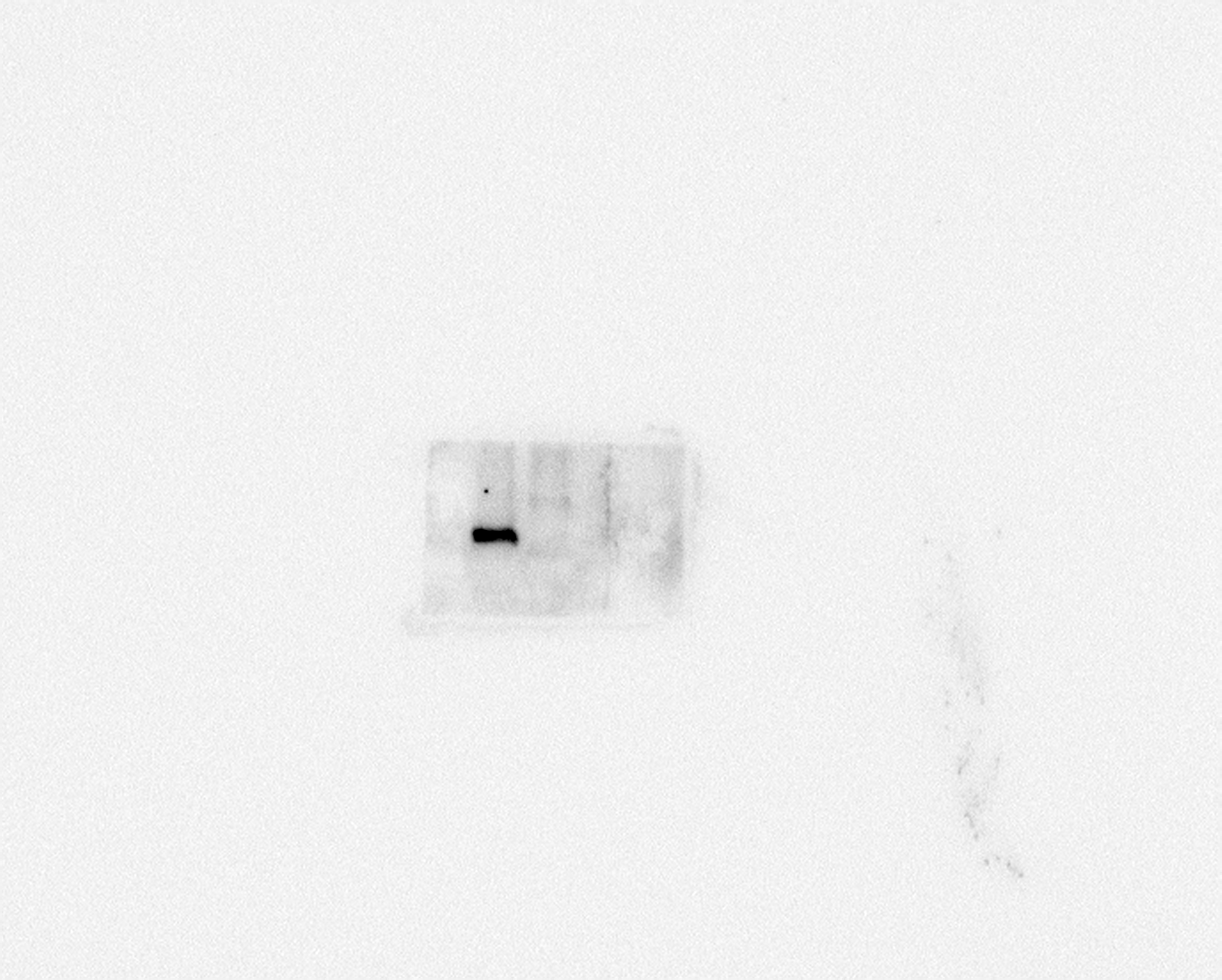

Supplement: Figure 4—source data 3. [file elife-73252-fig4-data3.zip › Figure 4-source data 3/unlabeled immublots for Figure 4/TA-KO-Figure 4B.Tif]

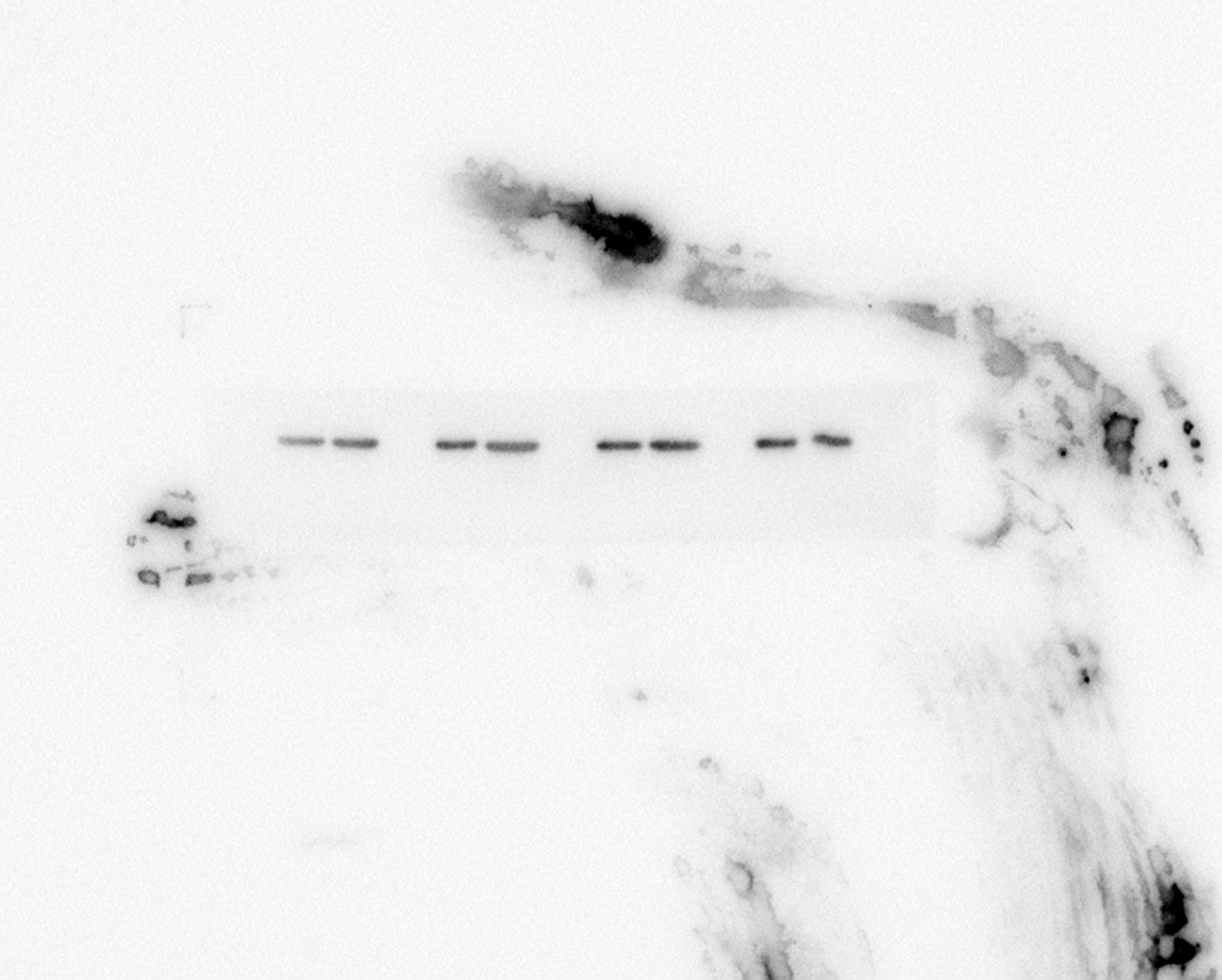

Supplement: Figure 4—source data 3. [file elife-73252-fig4-data3.zip › Figure 4-source data 3/unlabeled immublots for Figure 4/TG-KO-GAPDH 156 535 722 726-Figure 4C.Tif]

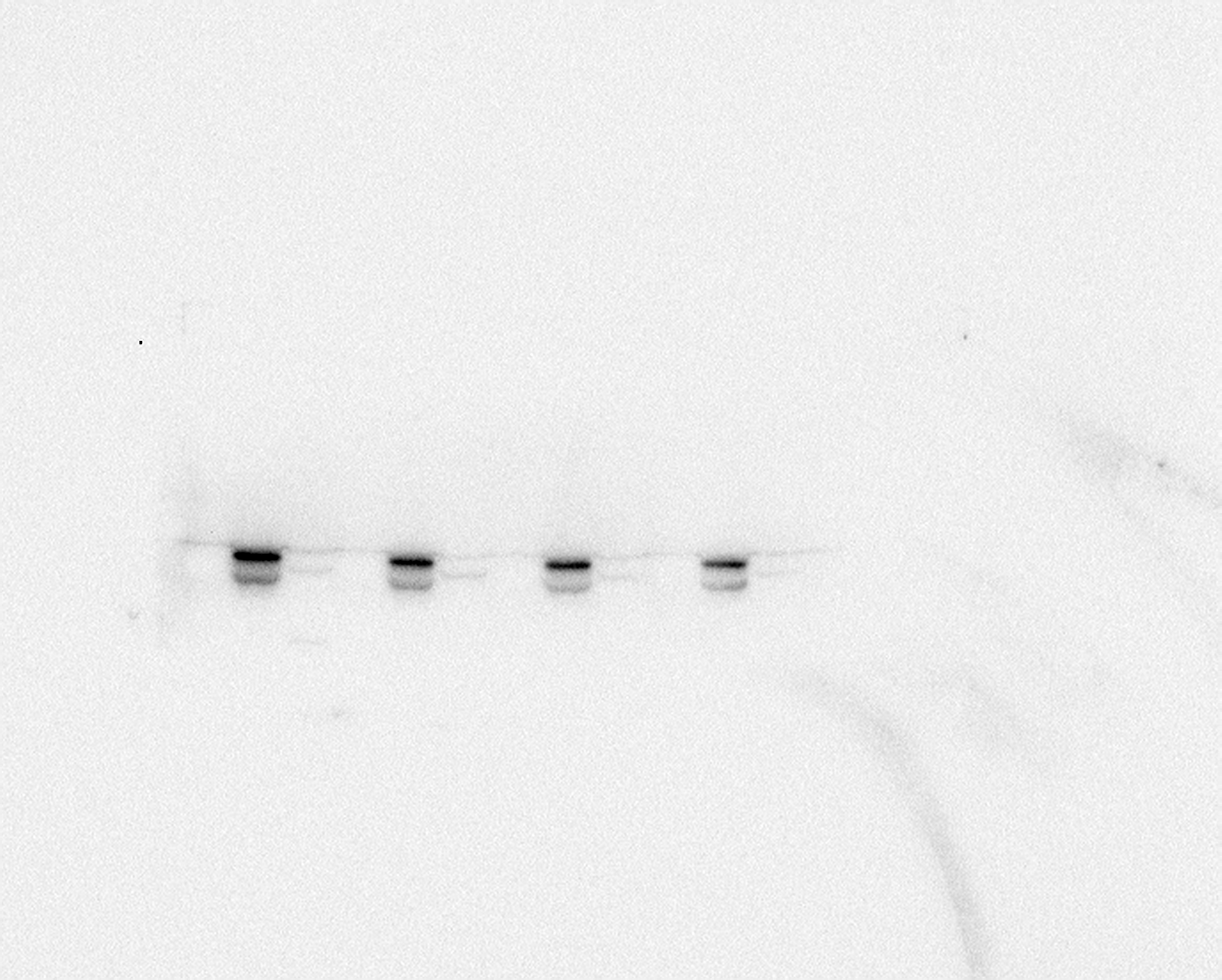

Supplement: Figure 4—source data 3. [file elife-73252-fig4-data3.zip › Figure 4-source data 3/unlabeled immublots for Figure 4/TG-KO156 535 722 726-3-Figure 4C.Tif]

**Figure 5C**

3 × FLAG-CEP170

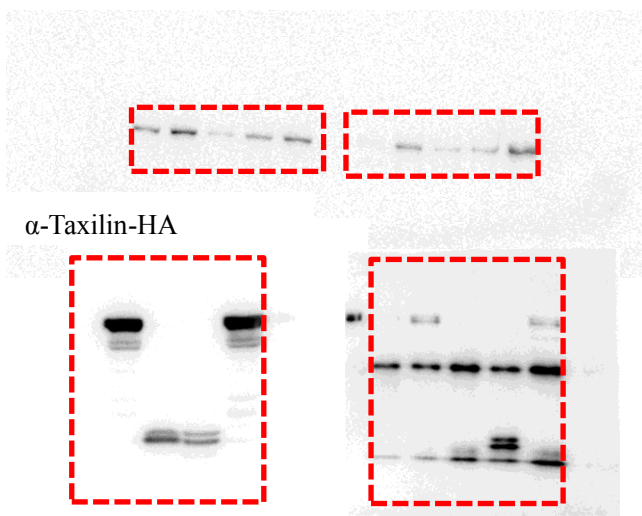

**Figure 5D**

3 × FLAG-CEP170

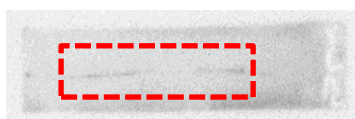

Supplement: Figure 5—source data 2. [file elife-73252-fig5-data2.zip › Figure 5-source data 2/Labeled immuoblots for Figure 5.pdf]

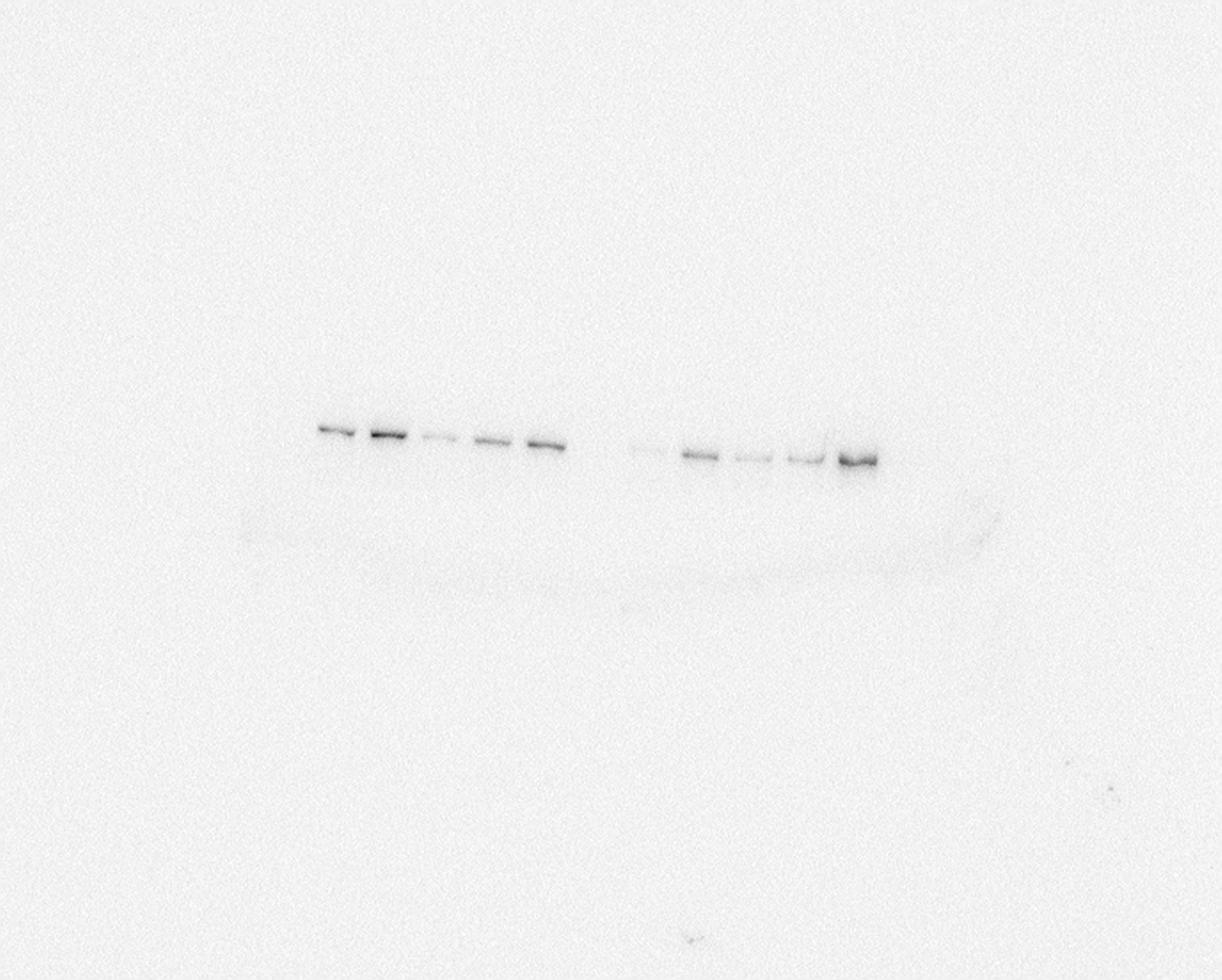

Supplement: Figure 5—source data 2. [file elife-73252-fig5-data2.zip › Figure 5-source data 2/unlabeled immunoblots for Figure 5/CEP170-IP-FLAG-Figure 5C.Tif]

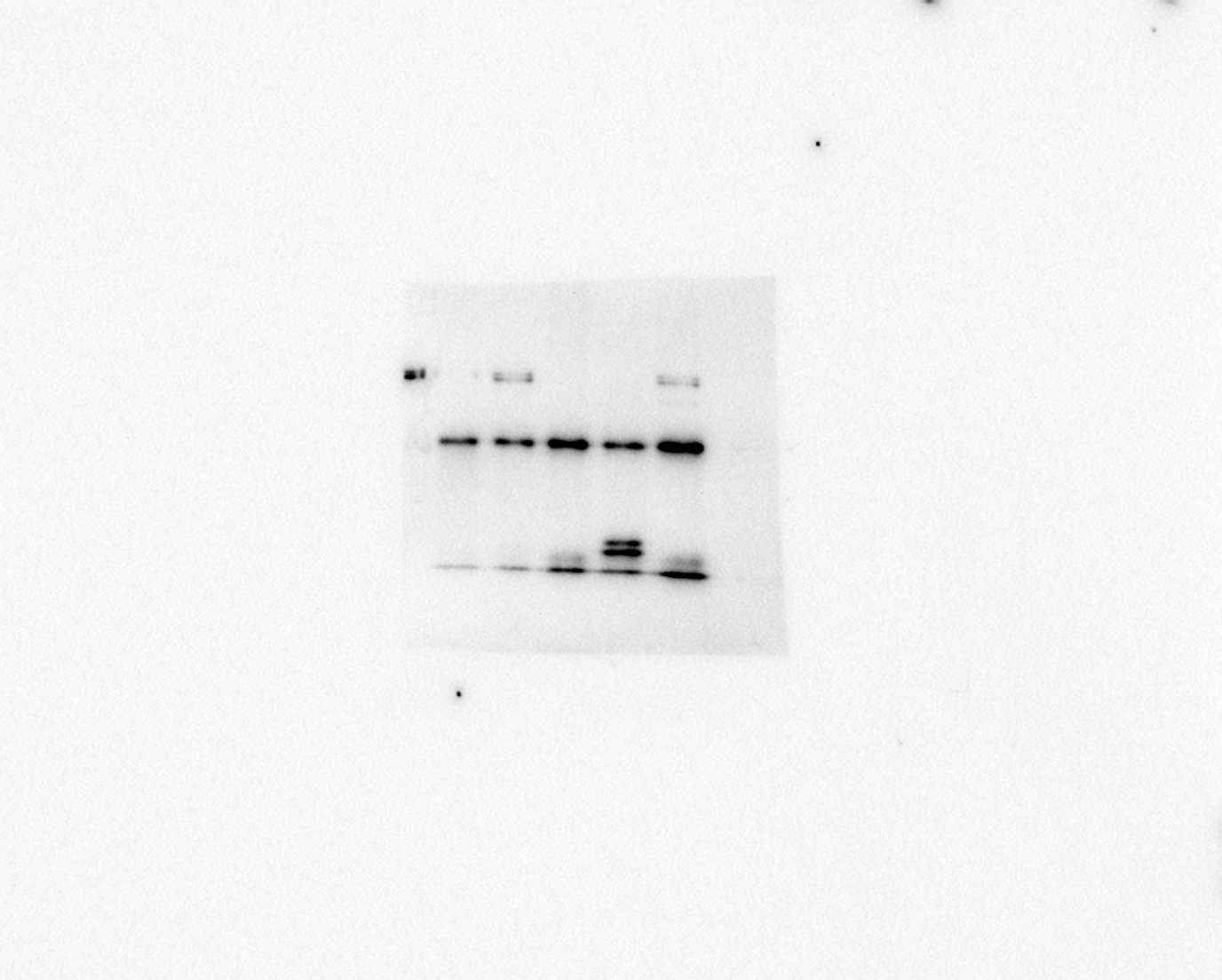

Supplement: Figure 5—source data 2. [file elife-73252-fig5-data2.zip › Figure 5-source data 2/unlabeled immunoblots for Figure 5/CEP170-Ip-TA-HA2-Figure 5C.Tif]

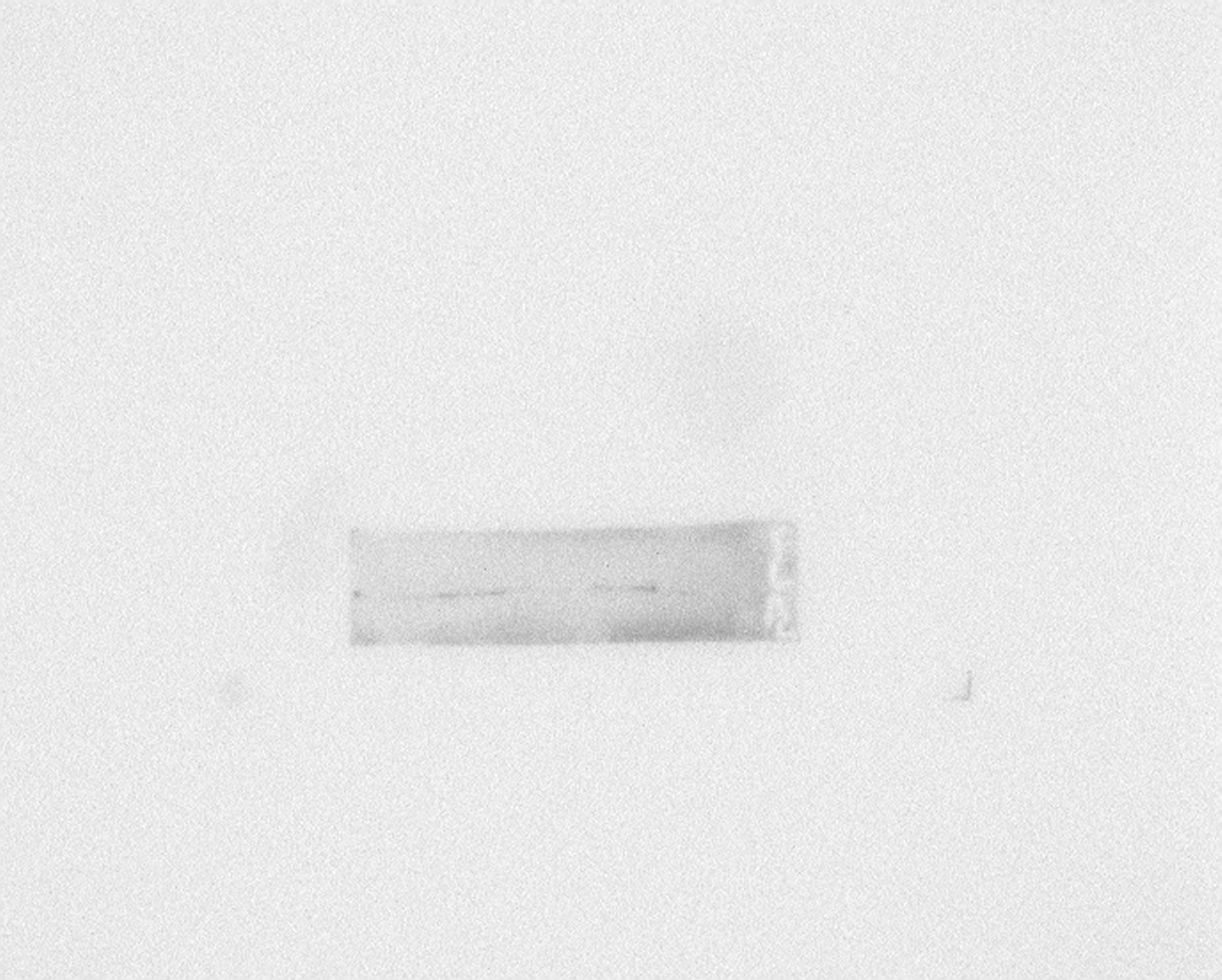

Supplement: Figure 5—source data 2. [file elife-73252-fig5-data2.zip › Figure 5-source data 2/unlabeled immunoblots for Figure 5/flag-170-2-Figure 5D.Tif]

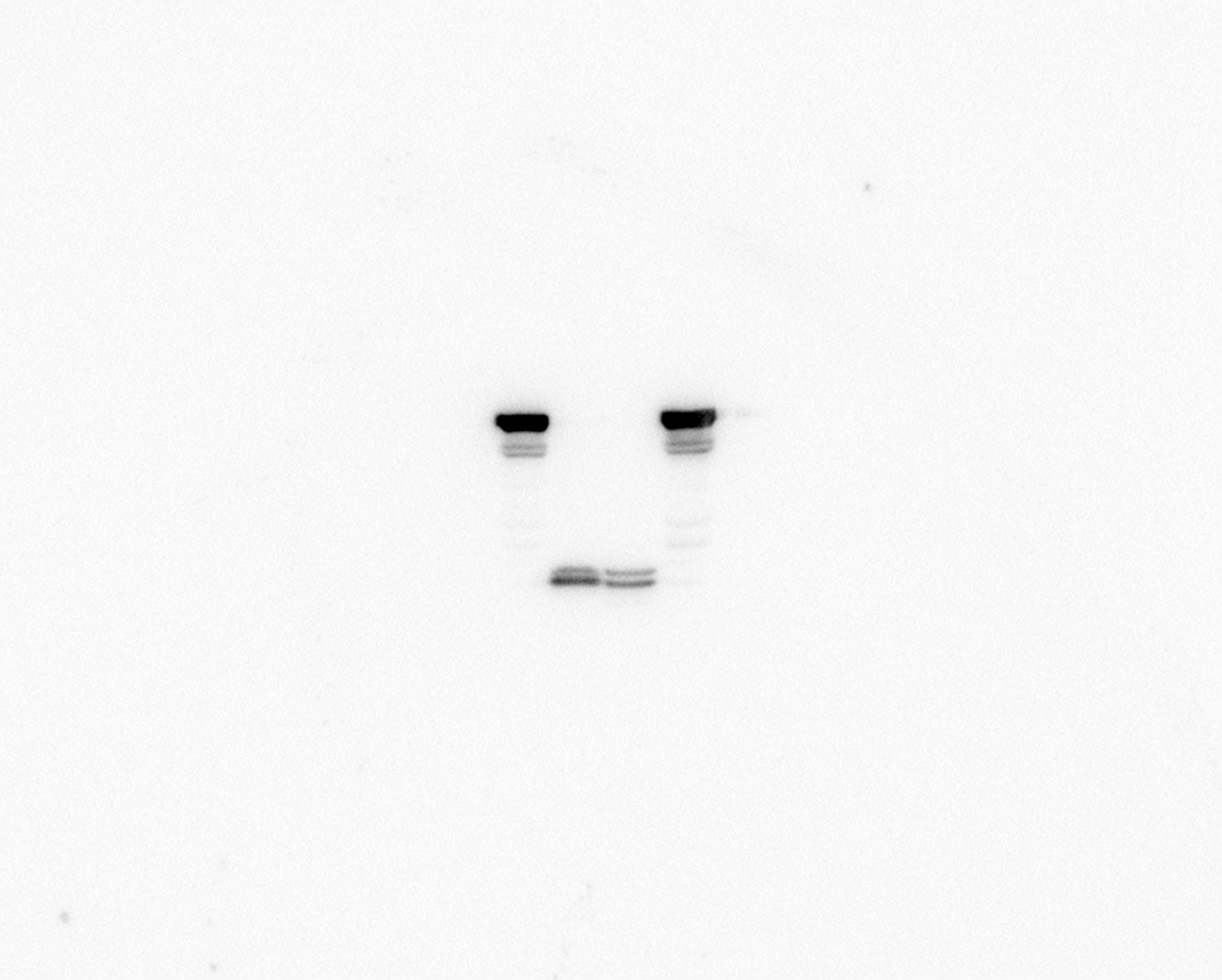

Supplement: Figure 5—source data 2. [file elife-73252-fig5-data2.zip › Figure 5-source data 2/unlabeled immunoblots for Figure 5/TXLNA-HA-Input2-Figure 5C.Tif]

**Figure 7A**

$\alpha$ -Taxilin

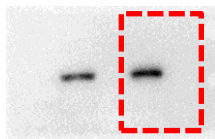

GAPDH

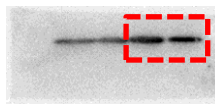

**Figure 7B**

$\gamma$ -Taxilin

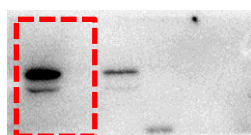

GAPDH

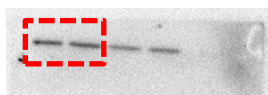

Supplement: Figure 7—source data 4. [file elife-73252-fig7-data4.zip › Figure 7-source data 4/Labeled immuoblots for Figure 7.pdf]

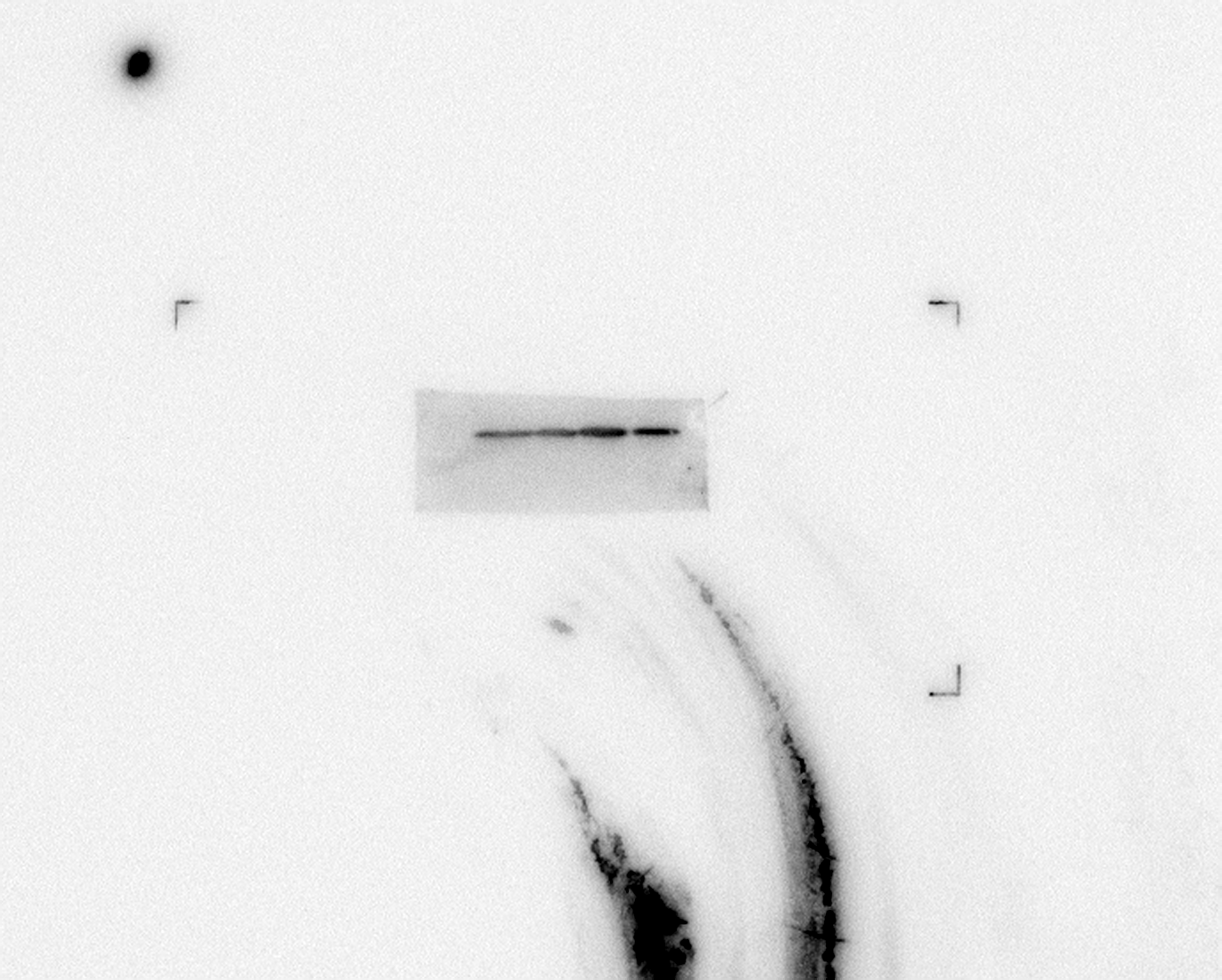

Supplement: Figure 7—source data 4. [file elife-73252-fig7-data4.zip › Figure 7-source data 4/unlabeled immublots for Figure 7/GAPDH for TAKO2-Figure 7A.Tif]

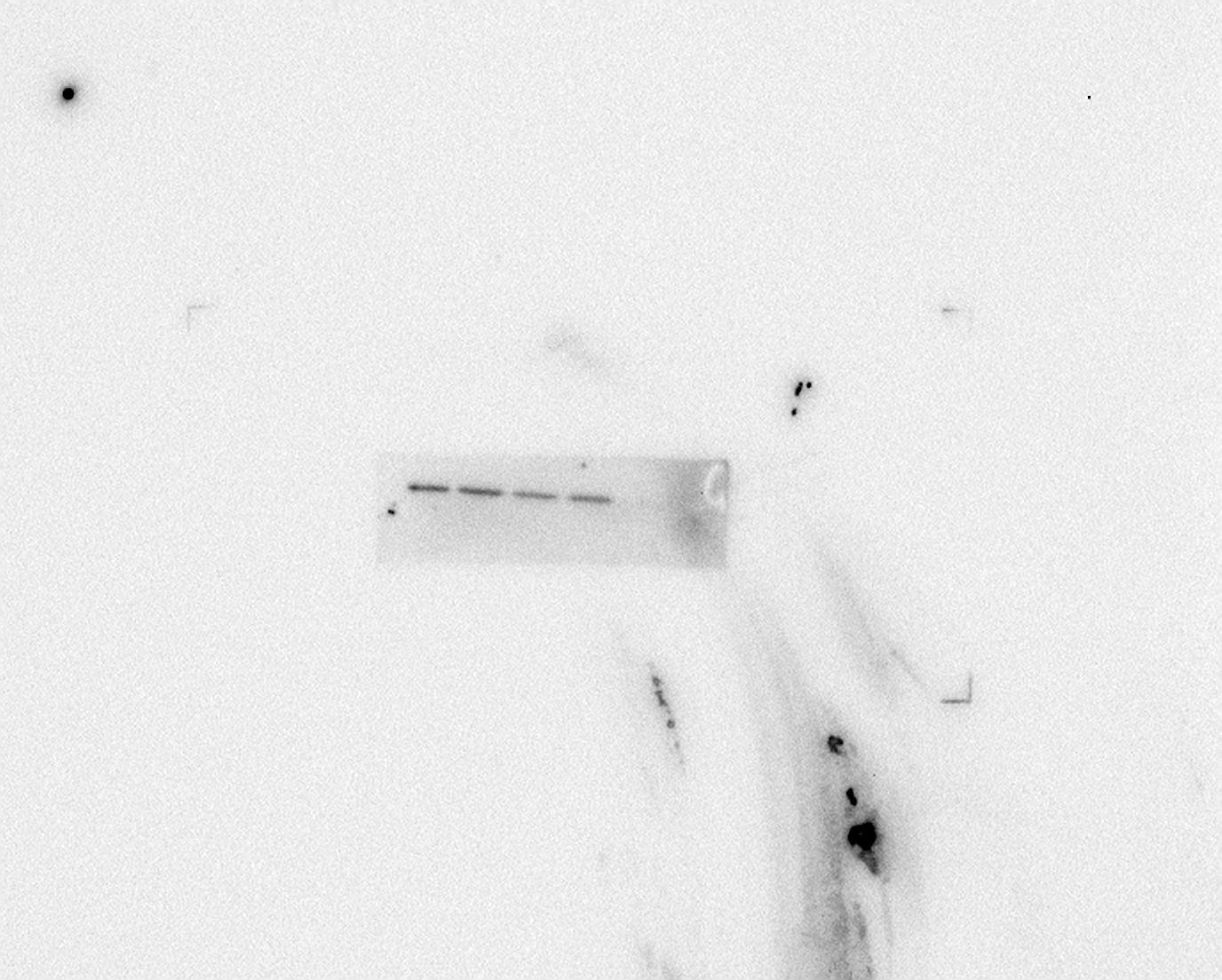

Supplement: Figure 7—source data 4. [file elife-73252-fig7-data4.zip › Figure 7-source data 4/unlabeled immublots for Figure 7/GAPDH for TGKO2-Figure 7B.Tif]

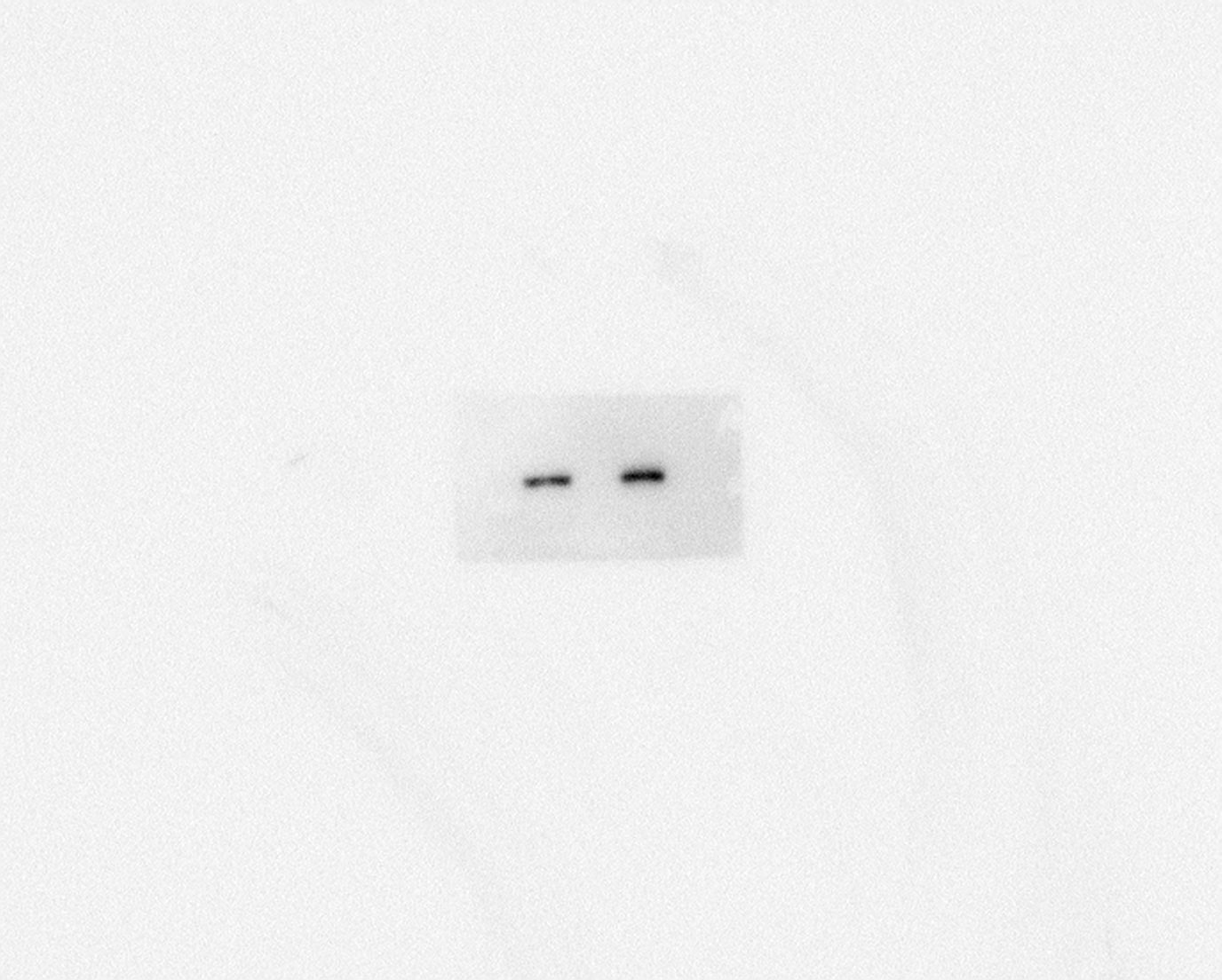

Supplement: Figure 7—source data 4. [file elife-73252-fig7-data4.zip › Figure 7-source data 4/unlabeled immublots for Figure 7/Hela-TA-KO6-16-FIgure 7A.Tif]

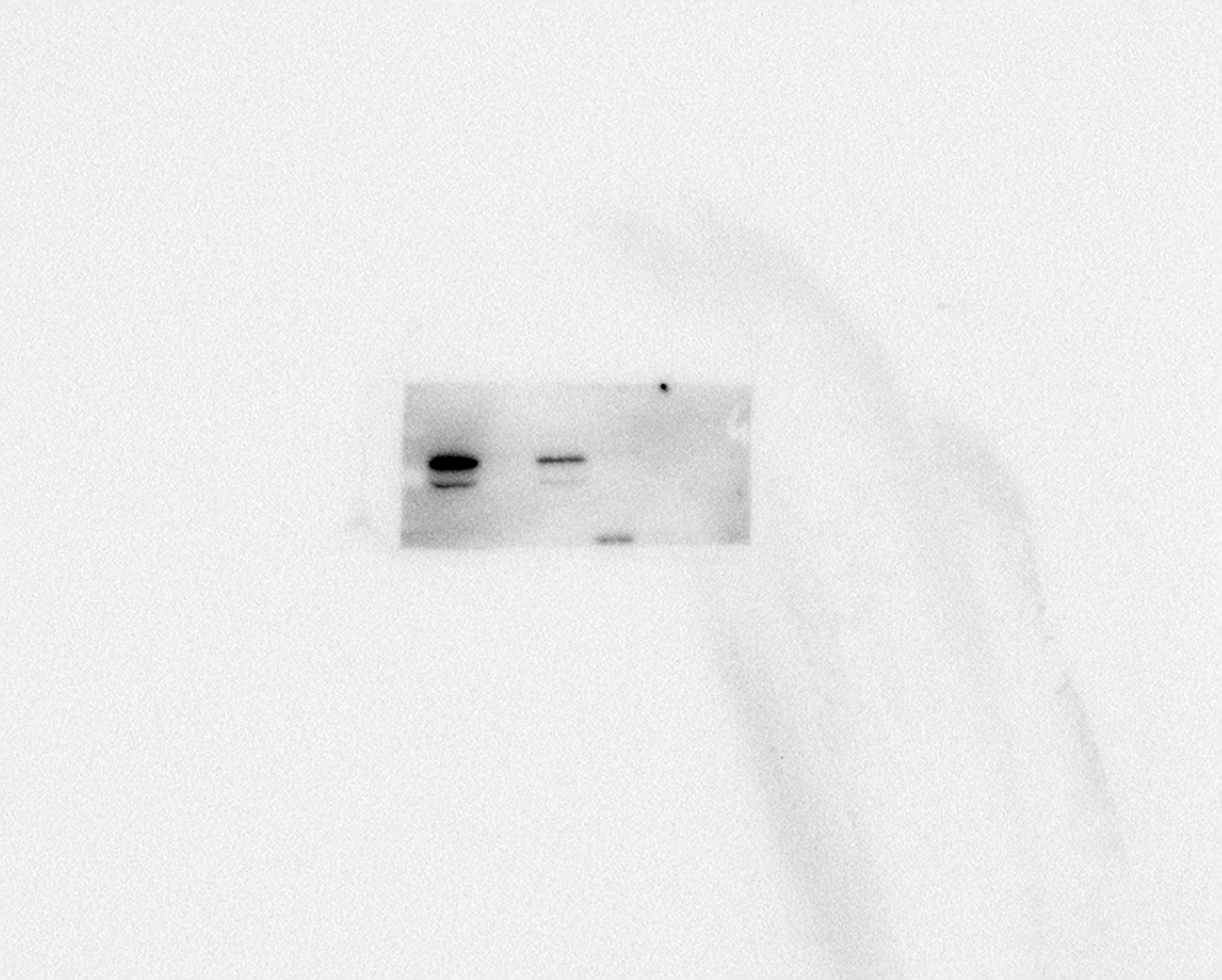

Supplement: Figure 7—source data 4. [file elife-73252-fig7-data4.zip › Figure 7-source data 4/unlabeled immublots for Figure 7/Hela-TG-KO9-16-Figure 7B.Tif]
