# Supplementary material for: α-/γ-Taxilin are required for centriolar subdistal appendage assembly and microtubule organization
Source: eLife. 2022 Feb 4;11:e73252. doi: 10.7554/eLife.73252 (PMC8816381; doi:10.7554/eLife.73252)

Figure 1-figure supplement 2B

α-Taxilin

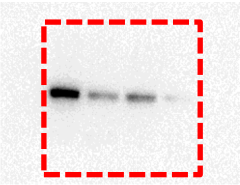

GAPDH

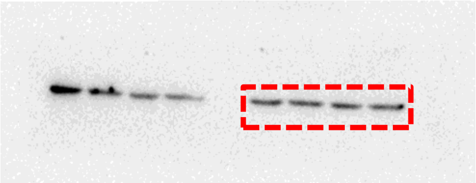

Figure 1-figure supplement 2C

γ-Taxilin

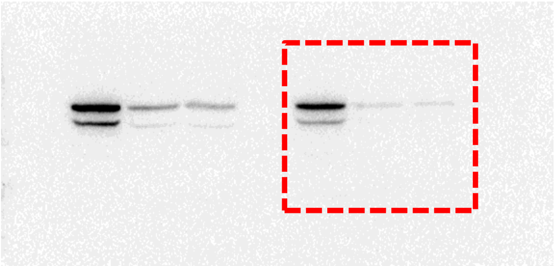

GAPDH

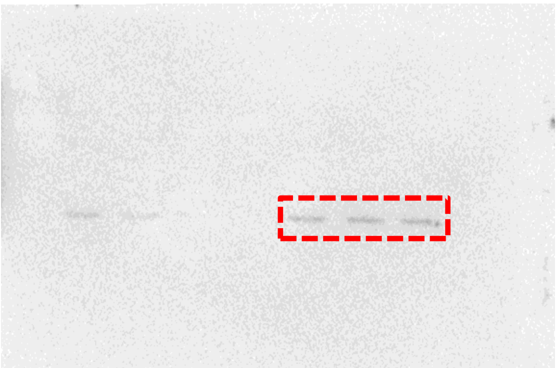

Figure 1-figure supplement 2E

HA

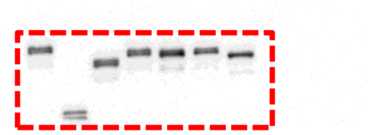

GAPDH

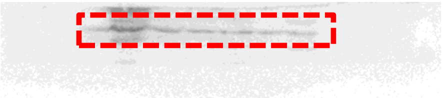

Figure 1-figure supplement 2F

GFP

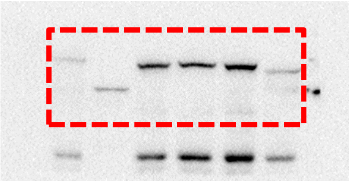

GAPDH

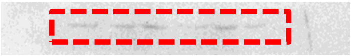

Supplement: Figure 1—figure supplement 2—source data 1. [file elife-73252-fig1-figsupp2-data1.zip › Figure 1-figure supplement 2-source data 1/Labeled immuoblots for Figure 1-figure supplement 2.pdf]
