# Supplementary material for: α-/γ-Taxilin are required for centriolar subdistal appendage assembly and microtubule organization
Source: eLife. 2022 Feb 4;11:e73252. doi: 10.7554/eLife.73252 (PMC8816381; doi:10.7554/eLife.73252)
Supplement: Figure 1—figure supplement 2—source data 3. [file elife-73252-fig1-figsupp2-data3.docx]

**Figure 1-figure supplement 2—source data 3.** Data of normalized γ-taxilin fluorescence intensity at centrosome of control- and γ-taxilin-siRNA treated RPE-1 cells (Data provided as Mean ± SEM).

|  | Control siRNA | γ-Taxilin siRNA#1 | γ-Taxilin siRNA#2 |
| --- | --- | --- | --- |
| Normalized γ-taxilin fluorescence intensity | 1.00±0.02 | 0.72±0.02 | 0.70±0.02 |
| n | 115 | 138 | 142 |
| *P*-value |  | <0.001 | <0.001 |
