# Supplementary material for: α-/γ-Taxilin are required for centriolar subdistal appendage assembly and microtubule organization
Source: eLife. 2022 Feb 4;11:e73252. doi: 10.7554/eLife.73252 (PMC8816381; doi:10.7554/eLife.73252)
Supplement: Figure 2—source data 1. [file elife-73252-fig2-data1.docx]

**Figure 2-source data 1.** The diameter of subdistal appendage proteins, including α-taxilin and γ-taxilin

| Protein | Diameter distance  (nm) | SD  (nm) | n |
| --- | --- | --- | --- |
| ODF2 | 320.59 | 15.26 | 14 |
| 3×FLAG-CCDC68 | 362.45 | 21.42 | 10 |
| CCDC120-3×FLAG | 375.90 | 19.64 | 9 |
| 3×FLAG-γ-taxilin | 483.54 | 19.64 | 12 |
| α-Taxilin | 547.47 | 27.44 | 7 |
| Ninein | 575.19 | 21.79 | 7 |
| CEP170 | 582.74 | 21.73 | 8 |
