# Supplementary material for: α-/γ-Taxilin are required for centriolar subdistal appendage assembly and microtubule organization
Source: eLife. 2022 Feb 4;11:e73252. doi: 10.7554/eLife.73252 (PMC8816381; doi:10.7554/eLife.73252)
Supplement: Figure 2—source data 2. [file elife-73252-fig2-data2.docx]

**Figure 2-source data 2.** The longitudinal positions of subdistal appendage (SDA) proteins, including α-taxilin and γ-taxilin

| Protein | Longitudinal distance  (nm) | SD  (nm) | n |
| --- | --- | --- | --- |
| ODF2 (lower level)  ODF2 (upper level) | 360.92  569.61 | 38.51  50.01 | 14  14 |
| 3×FLAG-CCDC68 | 472.64 | 24.83 | 13 |
| CCDC120-3×FLAG (lower level)  CCDC120-3×FLAG (upper level) | 458.01  635.53 | 33.97  33.78 | 11  11 |
| 3×FLAG-γ-Taxilin  α-taxilin | 570.29  453.31 | 33.53  38.26 | 12  16 |
| Ninein | 507.72 | 19.92 | 14 |
| CEP170 | 541.75 | 23.18 | 13 |
