# Supplementary material for: α-/γ-Taxilin are required for centriolar subdistal appendage assembly and microtubule organization
Source: eLife. 2022 Feb 4;11:e73252. doi: 10.7554/eLife.73252 (PMC8816381; doi:10.7554/eLife.73252)
Supplement: Figure 3—source data 1. [file elife-73252-fig3-data1.docx]

**Figure 3-source data 1. Data of normalized α-taxilin band intensity in control- and ODF2-siRNA treated RPE-1 cells (Data provided as Mean** ± **SEM)**

|  | Control siRNA | ODF2 siRNA |
| --- | --- | --- |
| Normalized α-taxilin band intensity | 1.00±0.13 | 0.96±0.10 |
| n | 6 | 6 |
| *P*-value |  | 0.56 |
