# Supplementary material for: α-/γ-Taxilin are required for centriolar subdistal appendage assembly and microtubule organization
Source: eLife. 2022 Feb 4;11:e73252. doi: 10.7554/eLife.73252 (PMC8816381; doi:10.7554/eLife.73252)
Supplement: Figure 3—source data 2. [file elife-73252-fig3-data2.docx]

**Figure 3-source data 2. Data of normalized γ-taxilin band intensity in control- and ODF2-siRNA treated RPE-1 cells (Data provided as Mean** ± **SEM)**

|  | Control siRNA | ODF2 siRNA |
| --- | --- | --- |
| Normalized **γ**-taxilin band intensity | 1.00±0.08 | 0.97±0.09 |
| n | 6 | 6 |
| *P*-value |  | 0.81 |
