# Supplementary material for: α-/γ-Taxilin are required for centriolar subdistal appendage assembly and microtubule organization
Source: eLife. 2022 Feb 4;11:e73252. doi: 10.7554/eLife.73252 (PMC8816381; doi:10.7554/eLife.73252)

Figure 3A

ODF2

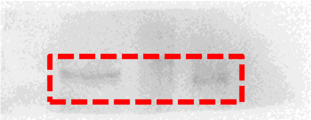

α-Taxilin

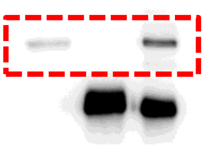

Figure 3B

ODF2

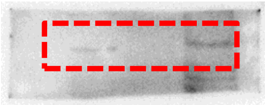

γ-Taxilin

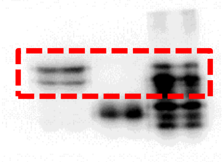

Figure 3E

α-Taxilin

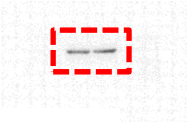

γ-Taxilin

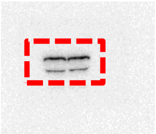

ODF2

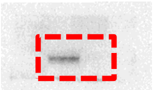

GAPDH

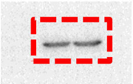

Supplement: Figure 3—source data 3. [file elife-73252-fig3-data3.zip › Figure 3-source data 3/Labeled immuoblots for Figure 3.pdf]
