# Supplementary material for: α-/γ-Taxilin are required for centriolar subdistal appendage assembly and microtubule organization
Source: eLife. 2022 Feb 4;11:e73252. doi: 10.7554/eLife.73252 (PMC8816381; doi:10.7554/eLife.73252)
Supplement: Figure 3—figure supplement 1—source data 3. [file elife-73252-fig3-figsupp1-data3.docx]

**Figure 3-figure supplement 1—source data 3.** Data of normalized α-taxilin fluorescence intensity at the centrosome of control- and CCDC68-siRNA treated RPE-1 cells (Data provided as Mean ± SEM).

|  | Control siRNA | CCDC68 siRNA |
| --- | --- | --- |
| Normalized α-taxilin fluorescence intensity | 1.00±0.02 | 0.96±0.02 |
| n | 56 | 62 |
| *P*-value |  | 0.1622 |
