# Supplementary material for: α-/γ-Taxilin are required for centriolar subdistal appendage assembly and microtubule organization
Source: eLife. 2022 Feb 4;11:e73252. doi: 10.7554/eLife.73252 (PMC8816381; doi:10.7554/eLife.73252)
Supplement: Figure 3—figure supplement 1—source data 4. [file elife-73252-fig3-figsupp1-data4.docx]

**Figure 3-figure supplement 1—source data 4.** Data of normalized γ-taxilin fluorescence intensity at the centrosome of control- and CCDC68-siRNA treated RPE-1 cells (Data provided as Mean ± SEM).

|  | Control siRNA | CCDC68 siRNA |
| --- | --- | --- |
| Normalized γ-taxilin fluorescence intensity | 1.00±0.03 | 1.03±0.02 |
| n | 64 | 73 |
| *P*-value |  | 0.31 |
