# Supplementary material for: α-/γ-Taxilin are required for centriolar subdistal appendage assembly and microtubule organization
Source: eLife. 2022 Feb 4;11:e73252. doi: 10.7554/eLife.73252 (PMC8816381; doi:10.7554/eLife.73252)
Supplement: Figure 3—figure supplement 1—source data 5. [file elife-73252-fig3-figsupp1-data5.docx]

**Figure 3-figure supplement 1—source data 5.** Data of normalized α-taxilin fluorescence intensity at the centrosome of control- and CCDC120-siRNA treated RPE-1 cells (Data provided as Mean ± SEM).

|  | Control siRNA | CCDC120 siRNA |
| --- | --- | --- |
| Normalized α-taxilin fluorescence intensity | 1.00±0.03 | 0.98±0.03 |
| n | 50 | 53 |
| *P*-value |  | 0.5719 |
