# Supplementary material for: α-/γ-Taxilin are required for centriolar subdistal appendage assembly and microtubule organization
Source: eLife. 2022 Feb 4;11:e73252. doi: 10.7554/eLife.73252 (PMC8816381; doi:10.7554/eLife.73252)
Supplement: Figure 3—figure supplement 1—source data 6. [file elife-73252-fig3-figsupp1-data6.docx]

**Figure 3-figure supplement 1—source data 6.** Data of normalized γ-taxilin fluorescence intensity at the centrosome of control- and CCDC120-siRNA treated RPE-1 cells (Data provided as Mean ± SEM).

|  | Control siRNA | CCDC120siRNA |
| --- | --- | --- |
| Normalized γ-taxilin fluorescence intensity | 1.00±0.02 | 1.00±0.01 |
| n | 93 | 96 |
| *P*-value |  | 0.9085 |
