# Supplementary material for: α-/γ-Taxilin are required for centriolar subdistal appendage assembly and microtubule organization
Source: eLife. 2022 Feb 4;11:e73252. doi: 10.7554/eLife.73252 (PMC8816381; doi:10.7554/eLife.73252)

Figure 3-Figure supplement 1G

α-Taxilin-HA

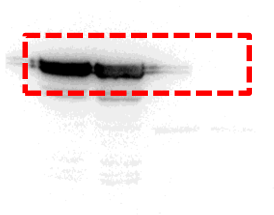

CCDC68-V5

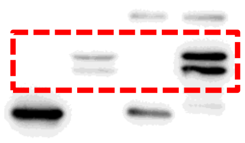

Figure 3-Figure supplement 1H

α-Taxilin-HA

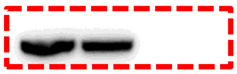

CCDC120-GFP

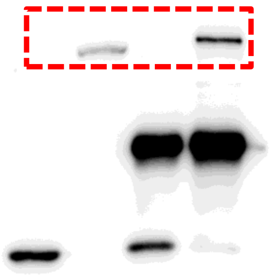

Figure 3-Figure supplement 1I

CCDC68-V5

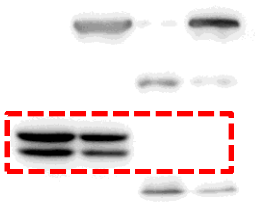

3×FLAG-γ-Taxilin

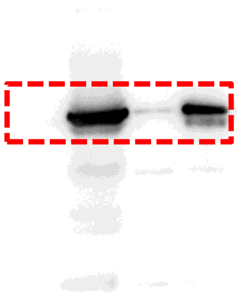

Figure 3-Figure supplement 1J

CCDC120-GFP

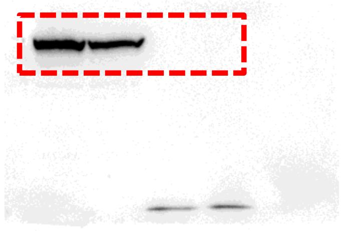

3×FLAG-γ-Taxilin

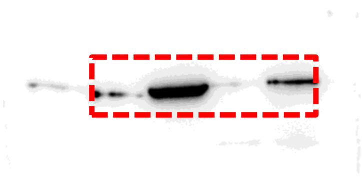

Figure 3-Figure supplement 1K

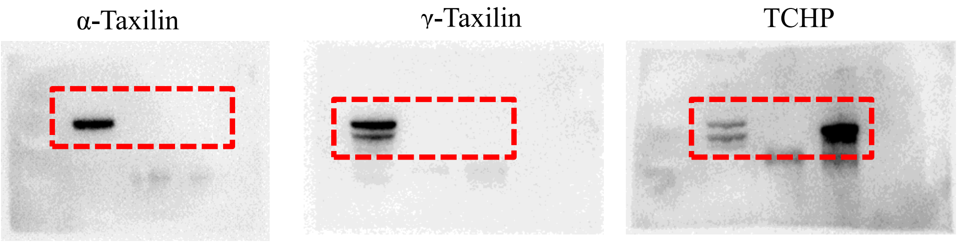

Supplement: Figure 3—figure supplement 1—source data 7. [file elife-73252-fig3-figsupp1-data7.zip › Figure 3-figure supplement 1-source data 7/Labeled immuoblots for Figure 3-figure supplement 7.pdf]
