# Supplementary material for: α-/γ-Taxilin are required for centriolar subdistal appendage assembly and microtubule organization
Source: eLife. 2022 Feb 4;11:e73252. doi: 10.7554/eLife.73252 (PMC8816381; doi:10.7554/eLife.73252)
Supplement: Figure 4—source data 1. [file elife-73252-fig4-data1.docx]

**Figure 4-source data 1. Centrosomal α-taxilin fluorescence intensities in wild-type (WT) and γ-taxilin knockout (KO) RPE-1 cells (Data provided as Mean** ± **SEM)**

|  | WT | γ-Taxilin KO |
| --- | --- | --- |
| Normalized α-taxilin fluorescence intensity | 1.00±0.02 | 0.42±0.01 |
| n | 120 | 126 |
| *P*-value |  | ＜0.0001 |
