# Supplementary material for: α-/γ-Taxilin are required for centriolar subdistal appendage assembly and microtubule organization
Source: eLife. 2022 Feb 4;11:e73252. doi: 10.7554/eLife.73252 (PMC8816381; doi:10.7554/eLife.73252)
Supplement: Figure 4—source data 2. [file elife-73252-fig4-data2.docx]

**Figure 4-source data 2. Centrosomal γ-taxilin fluorescence intensities in wild-type (WT) and α-Taxilin knockout (KO) RPE-1 cells (Data provided as Mean** ± **SEM)**

|  | WT | α-Taxilin KO |
| --- | --- | --- |
| Normalized γ-taxilin fluorescence intensity | 1.00±0.02 | 0.99±0.03 |
| n | 86 | 93 |
| *P*-value |  | 0.7917 |
