# Supplementary material for: α-/γ-Taxilin are required for centriolar subdistal appendage assembly and microtubule organization
Source: eLife. 2022 Feb 4;11:e73252. doi: 10.7554/eLife.73252 (PMC8816381; doi:10.7554/eLife.73252)

Figure 4A

α-Taxilin

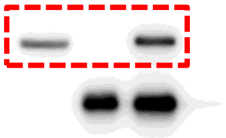

γ-Taxilin

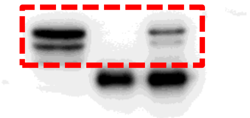

CEP170

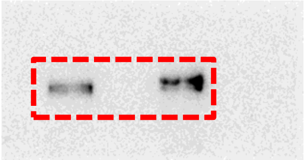

Ninein

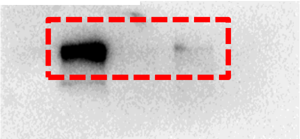

Figure 4B

α-Taxilin

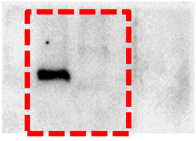

β-Actin

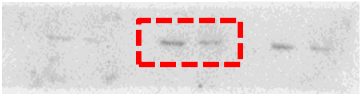

Figure 4C

γ-Taxilin

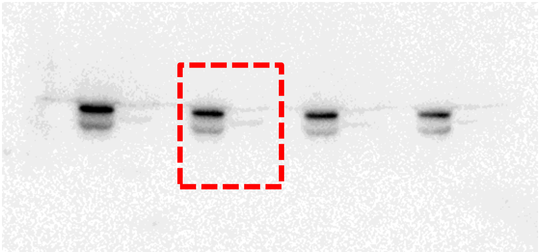

GAPDH

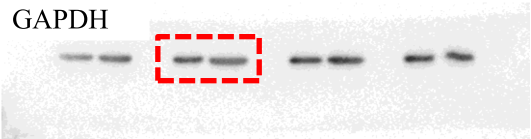

Figure 4F

3×FLAG-γ-Taxilin

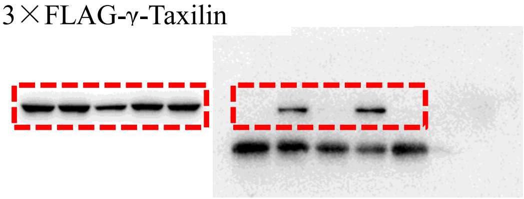

α-Taxilin-GFP

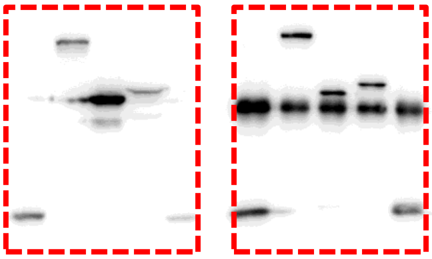

Figure 4I

GST-α-Taxilin-M

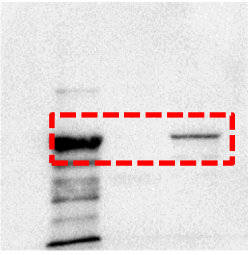

Figure 4H

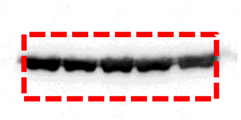

3×FLAG-α-Taxilin-IP

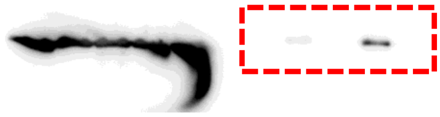

GFP-γ-Taxilin

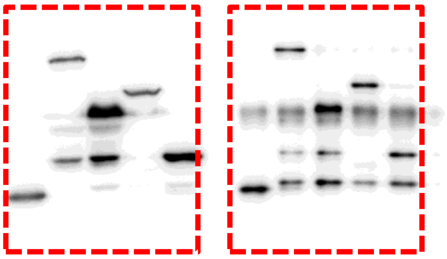

Supplement: Figure 4—source data 3. [file elife-73252-fig4-data3.zip › Figure 4-source data 3/Labeled immuoblots for Figure 4.pdf]
