# Supplementary material for: α-/γ-Taxilin are required for centriolar subdistal appendage assembly and microtubule organization
Source: eLife. 2022 Feb 4;11:e73252. doi: 10.7554/eLife.73252 (PMC8816381; doi:10.7554/eLife.73252)
Supplement: Figure 5—source data 1. [file elife-73252-fig5-data1.docx]

**Figure 5-source data 1. Data of centrosomal CEP170 fluorescence intensity in control and α-taxilin siRNA treated RPE-1 cells, and rescued by overexpressed full-length α-taxilin or the α-taxilin M2 deletion mutant (Data provided as Mean ± SEM)**

|  | Control siRNA | α-Taxilin siRNA#1 | α-Taxilin siRNA#1  +Res-α-taxilin-GFP | α-Taxilin siRNA#1  +Res-α-taxilin-△M2-GFP |
| --- | --- | --- | --- | --- |
| Normalized CEP170 fluorescence intensity | 1.00±0.02 | 0.78±0.02 | 1.08±0.02 | 0.77±0.02 |
| n | 101 | 102 | 105 | 111 |
