# Supplementary material for: α-/γ-Taxilin are required for centriolar subdistal appendage assembly and microtubule organization
Source: eLife. 2022 Feb 4;11:e73252. doi: 10.7554/eLife.73252 (PMC8816381; doi:10.7554/eLife.73252)
Supplement: Figure 6—source data 1. [file elife-73252-fig6-data1.docx]

**Figure 6-source data 1. Data of normalized centrosomal α-tubulin fluorescence intensity in wildtype (WT), *α-taxilin* knockout (KO) RPE-1 cells, and cells rescued by overexpressed 3×FLAG-α-taxilin or 3×FLAG-α-taxilin△M2 (Data provided as Mean** ± **SEM)**

|  | WT (n) | α-Taxilin KO (n) | α-TaxilinKO  +3×FLAG-α-taxilin (n) | α-TaxilinKO  +3×FLAG-α-taxilin**△**M2 (n) |
| --- | --- | --- | --- | --- |
| 0 min | 1.00±0.04 (44) | 0.98±0.05 (43) | 0.98±0.04 (43) | 0.90±0.04 (43) |
| 5 min | 1.00±0.03 (59) | 0.37±0.02 (46) | 0.76±0.02 (50) | 0.34±0.01 (42) |
| 10 min | 1.00±0.04 (59) | 0.62±0.03 (58) | 0.73±0.03 (48) | 0.56±0.02 (36) |
