# Supplementary material for: α-/γ-Taxilin are required for centriolar subdistal appendage assembly and microtubule organization
Source: eLife. 2022 Feb 4;11:e73252. doi: 10.7554/eLife.73252 (PMC8816381; doi:10.7554/eLife.73252)
Supplement: Figure 6—source data 2. [file elife-73252-fig6-data2.docx]

**Figure 6-source data 2. Data of normalized centrosomal α-tubulin fluorescence intensity in wildtype (WT), *γ-Taxilin* knockout (KO) RPE-1 cells, and rescued by overexpression of 3×FLAG-γ-taxilin or 3×FLAG-γ-taxilin△M2 (Data provided as Mean** ± **SEM)**

|  | WT (n) | γ-Taxilin KO (n) | γ-Taxilin KO  +3×FLAG-γ-taxilin (n) | γ-Taxilin KO  +3×FLAG-γ-taxilin**△**M2 (n) |
| --- | --- | --- | --- | --- |
| 0 min | 1.00±0.03 (63) | 0.94±0.03 (52) | 0.96±0.03 (45) | 0.92±0.02 (62) |
| 5 min | 1.00±0.02 (55) | 0.30±0.01 (55) | 0.55±0.02 (42) | 0.30±0.01 (59) |
| 10 min | 1.00±0.03 (52) | 0.46±0.02 (53) | 0.67±0.02 (47) | 0.24±0.02 (43) |
