# Supplementary material for: α-/γ-Taxilin are required for centriolar subdistal appendage assembly and microtubule organization
Source: eLife. 2022 Feb 4;11:e73252. doi: 10.7554/eLife.73252 (PMC8816381; doi:10.7554/eLife.73252)
Supplement: Figure 6—figure supplement 1—source data 1. [file elife-73252-fig6-figsupp1-data1.docx]

**Figure 6-figure supplement 1—source data 1.** Data of normalized areas of SDAs in wild-type (WT), *α-taxilin* and *γ-taxilin* KO RPE-1 cells (Data provided as Mean ± SEM).

|  | WT | *α-Taxilin* KO | *γ-Taxilin* KO |
| --- | --- | --- | --- |
| Normalized SDA area | 1.00±0.09 | 0.68±0.04 | 0.33±0.04 |
| n | 8 | 8 | 8 |
| *P*-value |  | <0.001 | <0.001 |
