# Supplementary material for: α-/γ-Taxilin are required for centriolar subdistal appendage assembly and microtubule organization
Source: eLife. 2022 Feb 4;11:e73252. doi: 10.7554/eLife.73252 (PMC8816381; doi:10.7554/eLife.73252)
Supplement: Figure 6—figure supplement 1—source data 2. [file elife-73252-fig6-figsupp1-data2.docx]

**Figure 6-figure supplement 1—source data 2. Data of normalized centrosomal α-tubulin fluorescence intensity in control-, α-taxilin-, or γ-taxilin-siRNA treated RPE-1 cells (Data provided as Mean** ± **SEM)**

|  | WT (n) | α-Taxilin siRNA#1 (n) | α-Taxilin siRNA#2 (n) | γ-Taxilin siRNA#1 (n) | γ-Taxilin siRNA#2 (n) |
| --- | --- | --- | --- | --- | --- |
| 0 min | 1.00±0.03 (151) | 1.01±0.03 (136) | 1.02±0.02 (155) | 1.08±0.03 113) | 1.00±0.02 154) |
| 5 min | 1.00±0.03 (136) | 0.59±0.02 (123) | 0.68±0.02 (120) | 0.67±0.03 (103) | 0.75±0.02 (121) |
| 10 min | 1.00±0.03 (105) | 0.50±0.02 (109) | 0.44±0.01 (153) | 0.48±0.01 (130) | 0.41±0.01 (137) |
