# Supplementary material for: α-/γ-Taxilin are required for centriolar subdistal appendage assembly and microtubule organization
Source: eLife. 2022 Feb 4;11:e73252. doi: 10.7554/eLife.73252 (PMC8816381; doi:10.7554/eLife.73252)
Supplement: Figure 6—figure supplement 1—source data 3. [file elife-73252-fig6-figsupp1-data3.docx]

**Figure 6-figure supplement 1—source data 3. Data of normalized centrosomal γ-tubulin fluorescence intensity in control-, α-taxilin-, or γ-taxilin-siRNA treated RPE-1 cells (Data provided as Mean** ± **SEM)**

|  | WT | α-Taxilin siRNA#1 | α-Taxilin siRNA#2 (n) | γ-Taxilin siRNA#1 (n) | γ-Taxilin siRNA#2 (n) |
| --- | --- | --- | --- | --- | --- |
| Normalized γ-tubulin fluorescence intensity | 1.00±0.02 | 1.04±0.02 | 1.05±0.02 | 1.06±0.03 | 1.00±0.02 |
| n | 119 | 128 | 106 | 117 | 97 |
| *P*-value |  | >0.05 | >0.05 | >0.05 | >0.05 |
