# Supplementary material for: α-/γ-Taxilin are required for centriolar subdistal appendage assembly and microtubule organization
Source: eLife. 2022 Feb 4;11:e73252. doi: 10.7554/eLife.73252 (PMC8816381; doi:10.7554/eLife.73252)
Supplement: Figure 7—source data 1. [file elife-73252-fig7-data1.docx]

**Figure 7-source data 1. Spindle angles of wild-type (WT), *α-taxilin* knockout (KO) HeLa cells, and cells overexpressed with indicated α-taxilin full-length and deletion mutants (Data provided as Mean ± SEM). (Data provided as Mean** ± **SEM)**

|  | WT | α-Taxilin KO | α-Taxilin KO  +3×FLAG-α-taxilin | α-Taxilin KO  +3×FLAG-α-taxilin**△**M2 | α-Taxilin KO  +3×FLAG-α-taxilin**△**M3 |
| --- | --- | --- | --- | --- | --- |
| Spindle angel (degree) | 5.13±0.43 | 11.92±0.95 | 5.54±0.46 | 8.03±0.83 | 4.92±0.58 |
| n | 61 | 60 | 60 | 61 | 60 |
