# Supplementary material for: α-/γ-Taxilin are required for centriolar subdistal appendage assembly and microtubule organization
Source: eLife. 2022 Feb 4;11:e73252. doi: 10.7554/eLife.73252 (PMC8816381; doi:10.7554/eLife.73252)
Supplement: Figure 7—source data 2. [file elife-73252-fig7-data2.docx]

**Figure 7-source data 2. Spindle angles of wild-type (WT), *γ-taxilin* knockout (KO) HeLa cells, and cells overexpressed with indicated γ-taxilin full-length and deletion mutants (Data provided as Mean** ± **SEM)**

|  | WT | γ-Taxilin KO | **γ**-Taxilin KO  +3×FLAG-**γ**-taxilin | **γ**-Taxilin KO  +3×FLAG-**γ**-taxilin**△**M1 | **γ**-Taxilin KO  +3×FLAG-**γ**-taxilin**△**M2 |
| --- | --- | --- | --- | --- | --- |
| Spindle angel (degree) | 4.94±0.46 | 10.94±0.93 | 4.85±0.44 | 4.35±0.40 | 7.89±0.82 |
| n | 60 | 60 | 60 | 60 | 61 |
