# Supplementary material for: α-/γ-Taxilin are required for centriolar subdistal appendage assembly and microtubule organization
Source: eLife. 2022 Feb 4;11:e73252. doi: 10.7554/eLife.73252 (PMC8816381; doi:10.7554/eLife.73252)
Supplement: Figure 7—source data 3. [file elife-73252-fig7-data3.docx]

**Figure 7-Source data 3. Astral microtubule length in wild-type (WT), *α-taxilin* and *γ-taxilin* knockout (KO) HeLa cells (Data provided as Mean** ± **SEM)**

|  | WT | α-Taxilin KO | γ-Taxilin KO |
| --- | --- | --- | --- |
| Astral microtubule length (nm) | 3.09±0.07 | 2.21±0.07 | 2.20±0.07 |
| n | 60 | 60 | 60 |
| *P*-value |  | <0.001 | <0.001 |
