# Supplementary material for: α-/γ-Taxilin are required for centriolar subdistal appendage assembly and microtubule organization
Source: eLife. 2022 Feb 4;11:e73252. doi: 10.7554/eLife.73252 (PMC8816381; doi:10.7554/eLife.73252)
Supplement: Figure 7—figure supplement 1—source data 2. [file elife-73252-fig7-figsupp1-data2.docx]

**Figure 7-figure supplement 1—source data 2.** Percentage of cells with centrosome separated over 2 µm in WT and *γ-taxilin* KO HeLa cells.

|  | WT | *γ-Taxilin* KO |
| --- | --- | --- |
| Percentage of cells with centrosome separated over 2 µm | 26.12%±1.29% | 67.01%±1.74% |
| n | 3 | 3 |
| *P*-value |  | <0.0001 |
